# Transducing chemical energy through catalysis by an artificial molecular motor

**DOI:** 10.1038/s41586-024-08288-x

**Published:** 2025-01-15

**Authors:** Peng-Lai Wang, Stefan Borsley, Martin J. Power, Alessandro Cavasso, Nicolas Giuseppone, David A. Leigh

**Affiliations:** 1 Department of Chemistry, University of Manchester, Manchester, UK; 2 School of Chemistry and Molecular Engineering, East China Normal University, Shanghai, China; 3 SAMS Research Group, Université de Strasbourg and Institut Charles Sadron, Strasbourg, France; 4 Institut Universitaire de France (IUF), Paris, France

**Keywords:** Supramolecular chemistry, Self-assembly

## Abstract

Cells display a range of mechanical activities generated by motor proteins powered through catalysis^1^. This raises the fundamental question of how the acceleration of a chemical reaction can enable the energy released from that reaction to be transduced (and, consequently, work to be done) by a molecular catalyst^2–7^. Here we demonstrate the molecular-level transduction of chemical energy to mechanical force^8^ in the form of the powered contraction and powered re-expansion of a cross-linked polymer gel driven by the directional rotation of artificial catalysis-driven^9^ molecular motors. Continuous 360° rotation of the rotor about the stator of the catalysis-driven motor-molecules incorporated in the polymeric framework of the gel twists the polymer chains of the cross-linked network around one another. This progressively increases writhe and tightens entanglements, causing a macroscopic contraction of the gel to approximately 70% of its original volume. The subsequent addition of the opposite enantiomer fuelling system powers the rotation of the motor-molecules in the reverse direction, unwinding the entanglements and causing the gel to re-expand. Continued powered twisting of the strands in the new direction causes the gel to re-contract. In addition to actuation, motor-molecule rotation in the gel produces other chemical and physical outcomes, including changes in the Young modulus and storage modulus—the latter is proportional to the increase in strand crossings resulting from motor rotation. The experimental demonstration of work against a load by a synthetic organocatalyst, and its mechanism of energy transduction^6^, informs both the debate^3,5,7^ surrounding the mechanism of force generation by biological motors and the design principles^6,10–14^ for artificial molecular nanotechnology.

## Abstract (web summary)

A cross-linked polymer gel driven by artificial molecular motors transforms chemical energy into mechanical force, achieving powered contraction and re-expansion, demonstrating a considerable advance in understanding energy transduction mechanisms and informing nanotechnology design principles.

## Main

Almost all biomolecular motors are catalysts^1^. They transduce energy from the fuel-to-waste reaction they catalyse—generally adenosine triphosphate plus water to adenosine diphosphate plus inorganic phosphate—to power the diverse array of tasks required by the cell, including transport, synthesis and force generation. Motor proteins have become exquisitely complex through evolution, making it difficult to provide fundamental answers to how the action of catalysis causes the energy from an accelerated reaction to be transduced^1^. Although a power stroke (a large-amplitude viscoelastic conformational change^2–5,7^) occurs during the mechanism of myosin (the force-generating motor protein in the muscle^8^), it is contested^3,5^ whether a power stroke is even necessary for force generation by molecular machines.

Artificial molecular motors^15–22^ and pumps^23–29^ can provide insights regarding the mechanisms of powered molecular-level motion^6,7,10,11,14^. Molecular machines have been interfaced with other components to perform tasks^24,27,30–32^, including the use of light-driven rotary motors to drive the contraction of gels^30–32^. However, performing work using artificial catalysis-driven molecular motors—the synthetic analogues of motor proteins—has remained elusive.

**Fig. 1:**
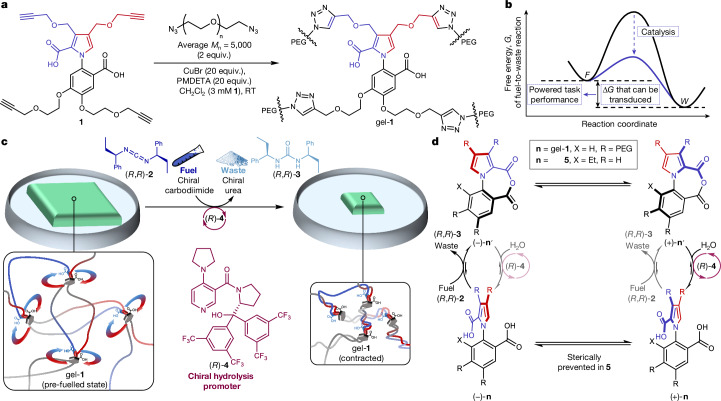
Contraction of a polymer gel with a covalently embedded, chemically fuelled molecular motor. **a**, Chemical structure of motor **1** and its incorporation into a cross-linked gel (gel-**1**) with motor units at the reticulation nodes through copper(i)-catalysed azide–alkyne cycloaddition (CuAAC) with azide-terminated PEG chains. RT, room temperature. **b**, Transduction of chemical energy by the catalysis of a fuel (*F*)-to-waste (*W*) reaction by a molecular ratchet. The energy available for transduction depends on the difference in the chemical potential between the fuel and waste and the kinetic asymmetry of the chemical engine cycle^6^. **c**, Treatment of gel-**1** with chiral fuel (*R*,*R*)-**2** and chiral hydrolysis promoter (*R*)-**4** leads to the directional rotation of the motor components through the catalysis^9^ of carbodiimide-to-urea hydration by the motor. This winds the polymer chains around each other, increasing writhe and creating new physical entanglements, resulting in gel contraction. The (*R*,*R*)-**2** and (*R*)-**4** fuelling system causes biased clockwise rotation of the pyrrole rotor about the phenyl stator in gel-**1**, increasing writhe in a (+)-helical sense in the polymer strands. **d**, Mechanism of continuous directionally biased 360° rotation of the motor units in gel-**1** and of the deracemization of motor model (±)-**5** with (*R*,*R*)-**2** and (*R*)-**4** under the fuelling conditions used for the gel experiments (dioxane-*d*_8_/D_2_O (8:5 v/v)).

A biaryl molecular rotary motor that operates through the organocatalysis of a fuel-to-waste reaction was recently reported^9^. The motor molecule catalyses a carbodiimide-to-urea fuel-to-waste reaction^33–35^, transiently forming an anhydride in which the motor accesses a different set of conformational dynamics to those available in the diacid state. The use of a chiral carbodiimide and a chiral hydrolysis promoter introduces kinetic asymmetry in the chemomechanical cycle^6,10,11,14,35^, resulting in the continuous directionally biased 360° rotation of the rotor about the stator. We considered if such a structurally simple catalysis-driven rotary motor could be incorporated into a soft-matter matrix and used to perform mechanical work through the transduction of chemical energy (Fig. 1b) by motor-molecule catalysis (Fig. 1c).

Motor **1** (Fig. 1a) is an analogue of the previously reported rotary motor^9^, derivatized with terminal alkynes extending from the 4- and 5-positions of the stator and 3- and 4-positions of the rotor to enable the attachment of the motor to azide-terminated polymers through copper-mediated azide–alkyne click cycloaddition^36^ (Supplementary Information section 2). Fuelling **1** with diisopropylcarbodiimide (DIC)^13,26,29,33,34^ in the presence of 4-dimethylaminopyridine (DMAP) resulted in transient anhydride formation (Supplementary Information section 4.1), indicating that the key acid-to-anhydride-to-acid chemical transformations in the chemomechanical cycle^9,26,29^ of the motor are unaffected by the additional functionalization.

## Motor-molecule incorporation into a gel framework

Tetra-alkyne **1** was treated with bisazide-terminated polyethylene glycol (PEG; number-average molecular weight (*M*_n_) = 5,000 g mol^−1^), CuBr and *N*,*N*,*N*′,*N*″,*N*″-pentamethyldiethylenetriamine (PMDETA) in CH_2_Cl_2_, forming gel-**1** through the chemical cross-linking of polymer chains at the motor nodes^30^ (Fig. 1a and Supplementary Information section 3.1). After the removal of copper salts and PMDETA by successive washings with CH_3_CN and aqueous sodium ethylenediaminetetraacetate (Na_4_-EDTA), gel-**1** was swollen in dioxane/H_2_O (8:5 v/v). This solvent system balances the need to swell gel-**1** with the solubility of the chemical fuel, and was used in all of the fuelling experiments reported in this study (Supplementary Information sections 3.2 and 3.3).

## Directional bias during fuelling

**Extended Data Fig. 1:**
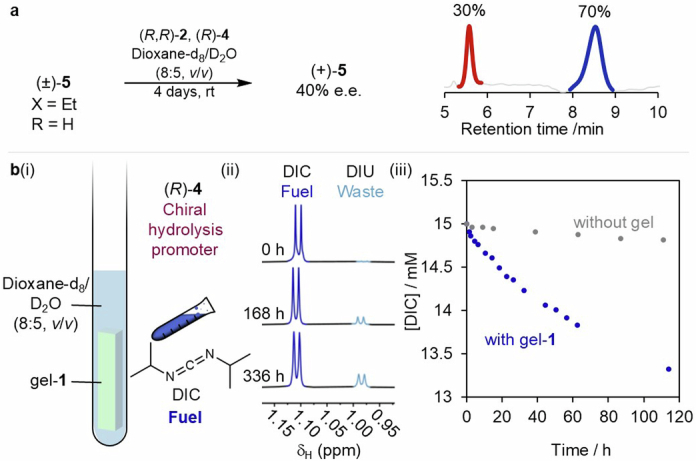
Directional bias and catalytic efficacy of gel-1 and model compounds. **a**, Chiral HPLC (ChiralPak-IF column, 25 °C, CH_2_Cl_2_:*i*-PrOH:CF_3_CO_2_H (95:5:0.1, *v*/*v*/*v*)) analysis showing 40% e.e. deracemisation of (±)-**5** under fuelling with (*R*,*R*)-**2** and (*R*)-**4** (Fig. 1d; Supplementary Information section 4.2); a directional bias of 2.3:1 clockwise:anti-clockwise rotation (i.e. a ‘mistake’ is made in directional rotation every 3 or 4 turns). **b**, (i) Catalysis of a carbodiimide-to-urea fuel-to-waste reaction by gel-**1** (Supplementary Information section 4.3). (ii) Partial ^1^H NMR spectra (dioxane-*d*_8_/D_2_O (8:5, *v*/*v*), 600 MHz, 298 K) showing progression of the fuel-to-waste reaction. Diisopropylcarbodiimide (DIC) was used as the fuel in this experiment as the ^1^H NMR resonances of DIC and diisopropylurea (DIU) used to monitor the reaction do not overlap with those of gel-**1**. (iii) Plot of the consumption of DIC fuel for the uncatalysed (grey) and gel-**1**-catalysed (blue) reactions, showing catalysis of the fuel-to-waste reaction by gel-**1**. Similar results were obtained using chiral fuel (*R*,*R*)-**2** or (*S*,*S*)-**2** instead of DIC (see Supplementary Information section 4.3).

A racemic model diacid (±)-**5** (ref. ^9^), which cannot fully rotate and, therefore, exists as an enantiomeric atropisomer, was treated with chiral carbodiimide (*R*,*R*)-**2** and hydrolysis promoter (*R*)-**4** in dioxane/H_2_O (8:5 v/v), producing urea (*R*,*R*)-**3** (Fig. 1d). The organocatalysis reaction resulted in a 40% enantiomeric excess of (+)-**5**, confirming that the chiral fuelling system generates similar directional rotational bias in the chemical engine cycle to the previous catalysis-driven motor molecule^9^ (Extended Data Fig. 1a and Supplementary Information section 4.2). We next confirmed that gel-**1** catalyses carbodiimide-to-urea hydration^13,34^ with an efficacy similar to that of the monomeric motor (Extended Data Fig. 1b and Supplementary Information section 4.3). These experiments demonstrated that (1) the diffusion of the fuelling reagents within the gel is not rate limiting^37^; (2) the rotary motors at the reticulation nodes of the swollen gel are accessible to the chemical fuel; and (3) when covalently embedded in the gel, motor **1** retains the ability to function as a catalyst for the fuel-to-waste reaction^9,35^.

## Fuelled gel contraction

**Fig. 2:**
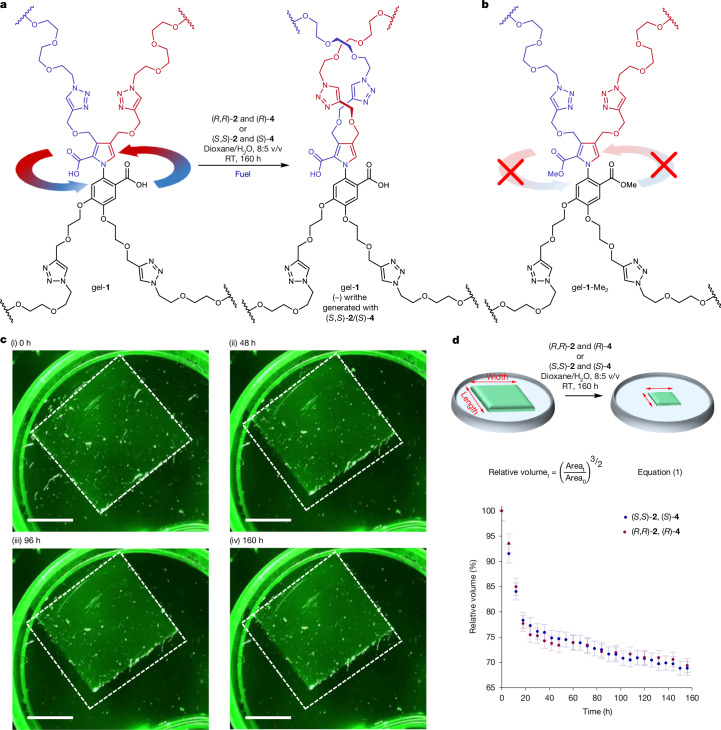
Macroscopic contraction of gel-**1** under chemical fuelling. **a**, Directional rotation of the rotor about the stator during catalysis by the motors embedded in gel-**1** results in the twisting of the polymer chains, inducing writhe (additional strand crossings; Supplementary Videos 1–4). **b**, Motor molecules in the methyl ester controlling gel-**1**-Me_2_ are unable to catalyse the fuel-to-waste reaction (nor can the methyl groups rotate past each other) and, therefore, do not directionally rotate under chemical fuelling (Supplementary Video 6 and Supplementary Information section 5.4.3). **c**, Images of gel-**1** during a chemically fuelled contraction with (*S*,*S*)-**2** and (*S*)-**4** (Supplementary Videos 1 and 2) at 0 h (i), 48 h (ii), 96 h (iii) and 160 h (iv). The white dashed line shows the outline of the gel before fuelling (*t* = 0). Scale bars, 3 mm. **d**, Contraction of gel-**1** under chemical fuelling. Equation (1) gives the decrease in volume (assuming isotropic contraction) based on the change in area of the horizontal face of the gel (moulded and then cut into the shape of a rectangular prism), pictured from above (the gel area is measured by image analysis using ImageJ 1.52a; Supplementary Information section 5.2). The percentage change in volume was plotted with respect to time, with contraction continuing under fuelling for 7 days. Fuelling carried out with (*S*,*S*)-**2** and (*S*)-**4** (blue data points) or (*R*,*R*)-**2** and (*R*)-**4** (red data points).

With an appropriate chemical fuelling protocol established, a thin square of gel-**1** (approximate dimensions, 10 × 10 × 1 mm^3^; around 0.08 mmol motor units) was treated with a solution of the chiral hydrolysis promoter (*S*)-**4** (4 mmol, 50 equiv.) in dioxane/H_2_O (8:5 v/v) at room temperature. The addition of (*S*)-**4** caused an expansion of the gel (approximately 10%) associated with diffusion-driven swelling^38^ and a likely change in the protonation state of the motor as (*S*)-**4** is basic (Supplementary Information section 5.4.1). Fuelling commenced (*t* = 0; Fig. 2d) by adding chiral carbodiimide (*S*,*S*)-**2** (8 mmol, 100 equiv.) in dioxane/H_2_O (8:5 v/v) only after the size of the gel was stable (4 h after the addition of (*S*)-**4**; Fig. 2a and Supplementary Information section 5.3). Contraction of the gel then occurred for 7 days (Fig. 2d (blue trace) and Supplementary Videos 1 and 2), resulting in an approximate 30% contraction of the gel (determined by measuring the decrease in surface area of the gel and assuming an isotropic effect on volume^32^ (Fig. 2d, equation (1) and Supplementary Information section 5.2). Even after the gel stopped contracting, the catalysis of carbodiimide hydration by the gel-embedded motors continued as long as unreacted fuel remained or if the fuel was replenished.

1 $${\rm{Relative}}\,{{\rm{volume}}}_{{\rm{t}}}={\left(\frac{{{\rm{Area}}}_{{\rm{t}}}}{{{\rm{Area}}}_{{\rm{0}}}}\right)}^{3/2}.$$

A dimethyl ester derivative of the gel, gel-**1**-Me_2_ (in which the carboxylic acid groups of the motor are methylated and are, therefore, unable to catalyse the hydration of the fuel (Fig. 2b)) displayed similar swelling when treated with (*S*)-**4** but no contraction after the addition of (*S*,*S*)-**2** (Supplementary Video 6 and Supplementary Information section 5.4.3).

**Extended Data Fig. 2:**
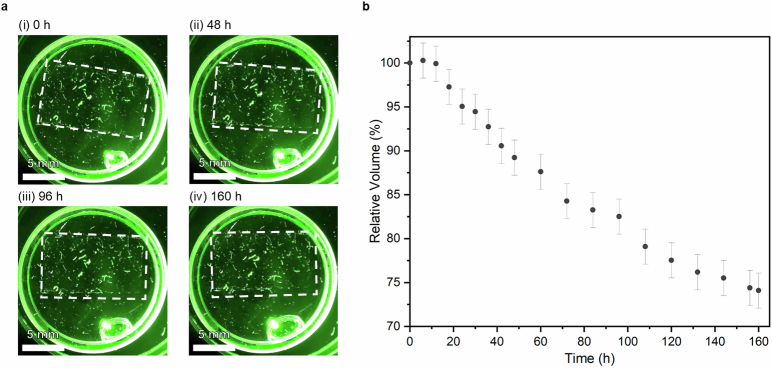
Macroscopic contraction of gel-1 under chemical fuelling with an achiral fuel and a chiral hydrolysis promoter. **a**, Images of gel-**1** during chemically fuelled contraction with (*S*)-**4** and DIC at (i) 0 h, (ii) 48 h, (iii) 96 h and (iv) 160 h. The white dashed line shows the outline of the gel prior to fuelling (*t* = 0). **b**, Contraction of gel-**1** under chemical fuelling. The change in %volume was plotted over time, with contraction continuing under fuelling for 7 days.

From the directionality obtained by fuelling (±)-**5** (Extended Data Fig. 1a), the directional rotation of the motor is anticlockwise with the (*S*,*S*)-**2** and (*S*)-**4** fuelling system, generating (–) writhe in the polymer chains of gel-**1**. Fuelling a pristine sample of gel-**1** with reagents of opposite chirality, that is, (*R*,*R*)-**2** and (*R*)-**4**, caused gel contraction with a similar rate and profile (Fig. 2d (red trace) and Supplementary Videos 3 and 4) to the (*S*,*S*)-**2** and (*S*)-**4** fuelling system. A clockwise rotation of the motor molecules results in twisting of the polymer strands to introduce writhe with a positive helical twist sense. However, fuelling a sample of gel-**1** with an achiral fuelling system (DIC and DMAP) led to no contraction of the gel, despite the efficient catalysis of the fuel-to-waste reaction by the gel-embedded motors (Supplementary Video 5 and Supplementary Information section 5.4.2; Extended Data Fig. 2 shows fuelling with achiral DIC and chiral hydrolysis promoter (*S*)-**4**).

## Fuel-contracted gel characterization

**Fig. 3:**
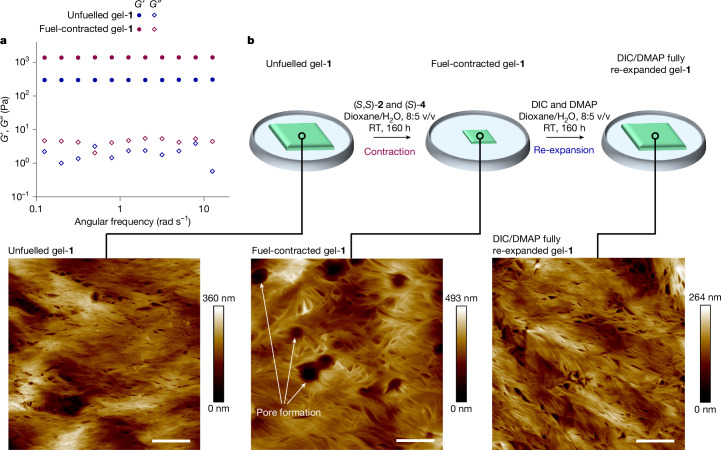
Rheological and AFM comparison of gel-**1** before and after chemically fuelled contraction and subsequent chemically fuelled re-expansion. **a**, Variation in *G*′ (storage modulus) and *G*″ (loss modulus) before and after gel contraction, as a function of angular frequency (Supplementary Information section 6). **b**, AFM images of gel-**1** before and after fuelled contraction, and after re-expansion under achiral fuelling (Supplementary Information section 6). The appearance of the micrometre-diameter pores reflects heterogeneities that are intrinsically present in such gels: regions with higher densities of motors produce more entanglements on fuelling, leaving free space (pores) between them^30^. The pores reduce in size or disappear on fuelled re-expansion of the gel. Scale bars, 2 µm.

**Extended Data Fig. 3:**
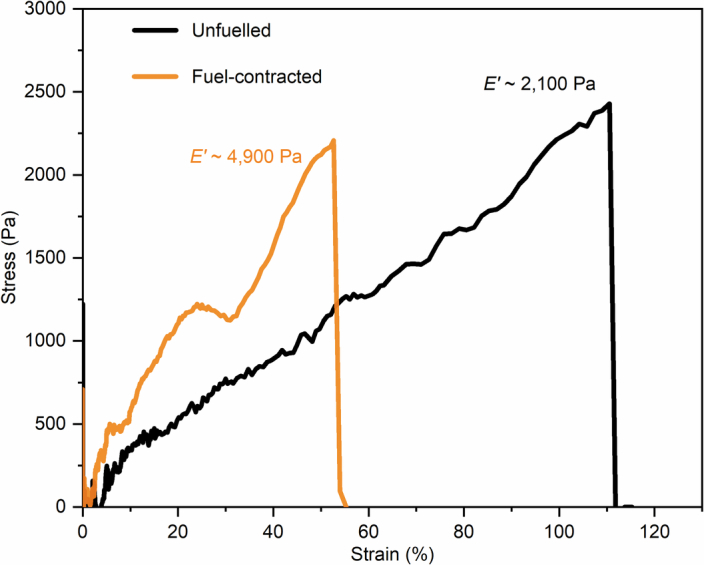
Tensile test of gel-1, unfuelled (black) and fuel-contracted (orange) with (*S*,*S*)-2 and (*S*)-4 after 7 days. The tensile tests show that the Young modulus of the fuel-contracted gel (4.9 kPa) is higher than that of the unfuelled gel (2.1 kPa), in agreement with the formation of new entanglements. We note that despite the increased stiffness of the gel upon fuel-driven contraction, the stress at break is still limited by defects in the gels (reaching 2.4 kPa for ~110% elongation in the unfuelled gel, and 2.25 kPa for ~55% elongation in the fuel-contracted gel).

The rheology of gel-**1** was compared before and after fuelling with (*S*,*S*)-**2** and (*S*)-**4** (Fig. 3a and Supplementary Information section 6). The storage (elastic) modulus (*G*′) of the gel was measured and found to be 2–3 orders of magnitude higher than the loss modulus (*G*″) in the measurability limit below 10 Pa. For both unfuelled and fuel-contracted gels, *G*′ is constant across the whole frequency range (Fig. 3a), which is typical for polymer gels at low frequency in the hydrodynamic regime. The storage modulus is proportional to the number of crossings multiplied by the elastic energy per chain. Therefore, the 4.7-fold increase in *G*′ (0.3 kPa for the unfuelled gel and 1.4 kPa for the fuel-contracted gel) indicates that an increase in the number of strand entanglements in the gel occurs as a result of fuelling^39,40^. The average value of 4.7 entanglements per motor in the contracted gel network (Supplementary Information section 8.2) is consistent with the kinetic asymmetry and fuel-use data measured in solution, which predict around five rotations based on the approximately 25 fuel-to-waste reactions catalysed by each motor in the gel. Tensile tests of gel-**1** show that the Young modulus (*E*′) of the contracted gel (4.9 kPa) is higher than that of the unfuelled gel (2.1 kPa), in agreement with the formation of new entanglements by fuelling (Extended Data Fig. 3 and Supplementary Information section 7). The stress at break reaches similar values of around 2.5 kPa for approximately 110% of elongation in the uncontracted form of the gel and for around 50% in its contracted form, in agreement with the topologically heterogeneous gel breaking at defect points^41^. The differences in rheology and mechanical properties between the unfuelled and fuel-contracted gels are a direct consequence of the winding of the polymer chains by catalysis-driven motor rotation at the nanoscale.

Effects caused by the twisting of the polymer strands in the fuelled gel were also evident at the microscopic scale (Supplementary Information section 6). Atomic force microscopy (AFM) images of the gel surface show changes in the polymer-chain conformation compared with the unfuelled gel, with the appearance of numerous kinks after contraction (Fig. 3b), reflecting an increase in writhe. The reduction in surface homogeneity and the appearance of micrometre-diameter pores in the gel after fuelling (Fig. 3b, fuel-contracted gel-**1**) can be attributed to the action of the winding of polymer chains around each other, generating large spaces empty of material in between denser, highly entangled regions^30^. On re-expansion (fuelling with DIC and DMAP), the microscopic structure of the gel reverts to being similar to that of the unfuelled gel (Fig. 3b, re-expanded gel).

All the macroscopic and microscopic features and measurements are indicative that the contraction of gel-**1** under fuelling with the chiral carbodiimide and hydrolysis promoter is a result of the organocatalysis-driven directional rotation of the motor molecules, increasing the entanglement (writhe^42^) of the polymer strands in the gel^30,43^.

## Chemomechanics at the stall force

The point at which gel contraction stops in the chiral fuelling experiments, despite fuel remaining and catalysis continuing, corresponds to the unravelling force exerted by the twisted polymer strands and the osmotic pressure from gel contraction, equalling the directional bias for rotation of the motor molecules under catalysis. This is the stall force, a typical performance measure of motor proteins^1–3^. How motor proteins respond to an applied force in chemical and conformational terms is often difficult to deconvolute clearly. However, because the mechanism of catalysis of the fuel-to-waste reaction by **1** is a well-defined series of steps, here it is apparent what the stall force corresponds to in terms of a chemomechanical mechanism. The directionality of rotation of **1** under catalysis (the ratcheting constant *K*_r_ (ref. ^12^) or Astumian’s *r*_0_ (ref. ^3^)) arises from the Curtin–Hammett asymmetry factor^7^ (*F*_C–H_) of the motor. This is determined by the two Curtin–Hammett-type kinetic resolutions in the reaction cycle (Fig. 1d). The force exerted by the twisted polymer strands affect the bias in the conformational exchange processes ((−)-**1**⇋(+)-**1** and (−)-**1**′⇋(+)-**1**′; Fig. 1d). The stall force corresponds to the point at which *F*_C–H_ = 1. This would be reached when the ratios [(−)-**1**]:[(+)-**1**] and [(−)-**1**′]:[(+)-**1**′], which are both 1:1 for the pairs of enantiomeric conformers in the absence of force, are driven away from 1:1 by the applied force such that at the stall force, they balance the Curtin–Hammett effect.

**Fig. 4:**
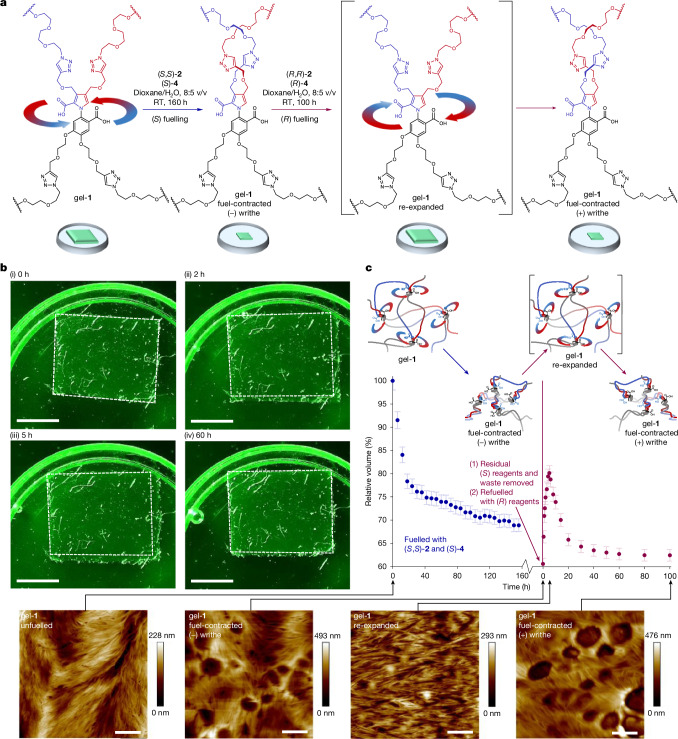
Chemically fuelled expansion–re-contraction of a sample of fuel-contracted gel-**1**. **a**, A gel-**1** sample that had been contracted with (*S*,*S*)-**2** and (*S*)-**4** (that is, the fuelling systems that drive the anticlockwise rotation of the motors) and then exhaustively washed to remove waste and residual fuel and hydrolysis promoter was then treated with (*R*,*R*)-**2** and (*R*)-**4** (that is, the fuelling system of opposite chirality) to power the rotation of the motor components in the opposite direction (that is, clockwise). The second fuelling system first powers the untwisting of the anticlockwise-twisted polymer strands, causing an expansion of the previously contracted gel (*t* = 0–5 h; red data points in **c**), after which the catalysis-driven rotation begins to reintroduce writhe of the opposite twist sense. **b**, Images of gel-**1** during this chemically fuelled expansion–re-contraction (Supplementary Videos 7 and 8) at 0 h (i), 2 h (ii), 5 h (iii) and 60 h (iv). The white dashed line shows the outline of the contracted gel before the second fuelling (*t* = 0, red data point). Scale bars, 3 mm. **c**, Percentage change in volume plotted with respect to time, showing the powered contraction–expansion–re-contraction behaviour of gel-**1** on successive fuelling with systems of opposite chirality (blue data points, (*S*,*S*)-**2** and (*S*)-**4**; red data points, (*R*,*R*)-**2** and (*R*)-**4**). The AFM image shows the appearance of the micrometre-diameter pores in the gel after fuelling with (*S*,*S*)-**2** and (*S*)-**4** to rotate the motors anticlockwise, generating (–) writhe in the polymer chains (second AFM image from the left). Subsequent fuelling of the contracted (–)-writhe gel with (*R*,*R*)-**2** and (*R*)-**4** rotates the motors clockwise. At the point of maximum re-expansion, when most of the original twists have been unwound, the AFM image shows that the number and size of pores are greatly reduced (third AFM image from the left). A continued clockwise rotation of the motors re-contracts the gel (with (+) writhe in the polymer strands), and micrometre-diameter pores reappear in the gel (AFM image on the right). Scale bars, 2 µm. Additional information is provided in Supplementary Information section 5.5.

Once the fuel supply is exhausted, the motor molecules are kinetically locked in the diacid state and the gel remains contracted (no relaxation/re-expansion of the gel occurs for the course of several months). However, the ability to select the direction of rotation of the motors according to the handedness of the fuel enables the macroscopic gel contraction to be reversed by powered unwinding of the polymer chains (Fig. 4, Supplementary Videos 7 and 8 and Supplementary Information section 5.5).

## Powering the motor molecules in reverse

A sample of gel-**1** was contracted to approximately 70% of its original size by fuelling with (*S*,*S*)-**2** and (*S*)-**4** to cause anticlockwise rotation and induce (–) twists in the polymer strands (0–160 h; Fig. 4c (blue data points)). The resulting (−)-writhe-contracted-gel was then washed extensively to remove waste and residual fuel and reagents, resulting in a further reduction in the volume of the now-‘empty’ (−)-writhe-contracted gel to 61% of its original size (Fig. 4c; red data point at 0 h of the second fuelling). The (−)-writhe-contracted gel was then re-swollen in dioxane/water (8:5 v/v) and (*R*,*R*)-**2** and (*R*)-**4** added to power the catalysis-driven rotation of the gel-embedded motors in the opposite direction (that is, clockwise). Under this fuelling regime, the (−)-writhe-contracted gel first expanded for 5 h from the 61% minimum volume to a maximum of 81% of the original gel size (Fig. 4c, red data points). Approximately 10% of this expansion can be attributed to diffusion-driven swelling from the addition of the new batch of fuel and hydrolysis promoter, but much more modest expansion in control experiments (second fuellings with (*S*,*S*)-**2**/(*S*)-**4** or DIC/DMAP; Supplementary Information section 5.5 and Supplementary Video 9) indicate that the substantial expansion results from the powered clockwise unwinding of the anticlockwise-twisted polymer strands. After the maximum size of the gel is reached under fuelling with the (*R*,*R*)-**2** and (*R*)-**4** fuelling system (5 h; Fig. 4c (red data points)), the rotation of the motors under catalysis continues in the clockwise direction and the gel begins to re-contract, reaching a minimum volume of 62% after approximately 60 h of fuelling with (*R*,*R*)-**2** and (*R*)-**4** (Fig. 4c (red data points), Supplementary Videos 7 and 8 and Supplementary Information section 5.5). The powered re-expansion–re-contraction of the contracted gel is, again, accompanied by the closing and reopening of the micrometre-scale pores observable by AFM (Fig. 4c and Supplementary Fig. 14).

The original volume of the gel is not fully recovered by the powered expansion (Fig. 4c, red data points). This is as expected because all the originally twisted strands will not become fully unwound at the same time. Once an anticlockwise-twisted strand pair becomes fully unwound by the now-clockwise-rotating motor, that strand pair will continue to be twisted clockwise by the motor, winding those strands around each other again before some of the other, anticlockwise-twisted strand pairs in the gel have been fully unwound. As such, expansion mediated by powered directional rotation does not return the gel to its equilibrium state (although fuelling with the achiral fuelling system does; see Supplementary Information section 5.5.3), but rather drives the gel from one out-of-equilibrium state to another. Notably, the rate of re-expansion (0–5 h; Fig. 4c (red data points)) is considerably faster than the rate of subsequent contraction (5–60 h; Fig. 4c (red data points)). We attribute this to the re-expansion being accelerated by the untwisting force exerted by the twisted strands, improving the kinetic asymmetry (stereochemical bias) of the chemically gated steps of the motor rotary catalysis. The release of elastic energy stored in the contracted gel to accelerate this process is a direct result of energy being transduced from the fuel-to-waste reaction by motor rotational catalysis during the first fuelled contraction (Fig. 4c (blue data points)).

## Energy transduction through kinetic asymmetry

Current generations of artificial chemically (for example, pH) responsive gels and polymers used for actuation operate through switching^44,45^. By contrast, the motor-molecule units in gel-**1** generate force by fuelling the biasing of the kinetics of ground-state conformational changes to achieve kinetic asymmetry—the same type of catalysis-driven information ratchet mechanism as that found in biological motor molecules. In doing so, work is accumulated; each turn of a motor progressively adds to the force generated and work done. This is fundamentally different to switching, where any work done by the change in state of the switch is undone by a full operating cycle^11,45–48^. The generation of force and performing of work by gel-**1** is reminiscent of myosin II, the motor protein that powers muscle contraction in most animal cells^8^ (although gel-**1** acts through rotary rather than linear molecular-level dynamics). Myosin II is a hexameric protein with a molecular mass of 450 kD, whereas each organocatalytic motor unit in gel-**1** consists of just 17 non-hydrogen atoms. Nevertheless, in both cases, the action of catalysis causes force to be generated and work to be done by the catalyst transducing chemical energy from the catalysed reaction to mechanical energy and elastic energy storage (Fig. 1b). The simplicity of the synthetic motor system means that the chemomechanical mechanism by which this happens is clearly apparent. As both key conformational changes in the ratcheting cycle occur between enantiomeric atropisomers ((+)-**1** and (–)-**1**; (+)-**1**′ and (−)-**1**′), there is no power stroke. The experimental demonstration of the transduction of chemical energy to perform work against a load through kinetic asymmetry provides a minimalist mechanistic illustration of how catalysis-driven molecular motors can extract order from chaos^4^.

## Methods

The synthesis of all the compounds and detailed experimental procedures for all the other experiments are given in the Supplementary Information.

### Representative method for formation of gel-**1**

A solution of **1** (0.7 mg, 1.2 µmol) in degassed CH_2_Cl_2_ (200 µl) was added to a solution of polyoxyethylene bis(azide) (average *M*_n_, 5,000; 12 mg, 2.4 µmol), CuBr (3.4 mg, 24 µmol) and PMDETA (5 µl, 24 µmol) in degassed CH_2_Cl_2_ (200 µl). The gel was formed in 10 min and was washed extensively with CH_3_CN, Na_4_-EDTA and water. A colourless and transparent gel-**1** was obtained (7.6 mg dry gel, 60% yield).

To form gels in a uniform shape suitable for the fuel contraction experiments, after the reagents were mixed (at the point of gel formation discussed above), the mixture was quickly homogenized (<2 min) and transferred to a custom-made stainless steel mould of 20 × 20 × 1 mm^3^ (height × width × depth). After 30 min, the gel was transferred from the mould to a closed bottle with CH_3_CN (50 ml), and agitated on a shaking plate (shaking frequency, 160 turns per minute) for 30–45 min. The procedure was repeated, washing the gel with different solvents in the following sequence: CH_3_CN, H_2_O, EDTA solution at pH 9 (11.7 g l^−1^ adjusting the pH to 9.0 with approximately 2 M NaOH), H_2_O, EDTA solution at pH 9, H_2_O (twice), a mixture of H_2_O/dioxane (5:8 v/v; twice). The total number of washings was ten.

### General fuel contraction of gel-**1**

(*S*)-**4** was dissolved in dioxane/H_2_O (800 µl, 8:5 v/v) in a cylindrical quartz cell (*φ*, 17 × 6 mm^2^). A square sample of gel measuring approximately 10 × 10 × 1 mm^3^ was added to the solution. The gel was allowed to equilibrate for 4 h. Subsequently, (*S*,*S*)-**2** was added and the gel size was monitored by video recording using a Veho DX-3 USB camera.

### AFM

AFM images were obtained by scanning the samples using Nanoscope 8 (Bruker) operated in the peak-force tapping mode. Peak-force AFM is based on the peak-force tapping technology, during which the probe is oscillating in a similar fashion as it is in tapping mode, but far below the resonance frequency. Each time the tip and the sample are brought together, a force curve is captured. These forces can be controlled at levels much lower than the contact mode and even lower than the tapping mode, enabling operation on the most delicate, soft samples, as is the case here. An ultrasharp silicon tip on a nitride lever was used (Bruker, ScanAsyst; spring constant of 0.4 N m^–1^ and tip radius of about 5 nm). During AFM imaging, the force was reduced to avoid dragging the molecules by the tip. Here the applied peak force is about 50 pN. All the analyses of the images were conducted with integrated software.

### Rheology measurements

Rheology measurements were performed using a TA Instruments hybrid rheometer HR-3 equipped with standard Peltier parallel steel plates with temperature control (20 mm diameter) set at 25 °C, and with a solvent trap to create a thermally stable vapour barrier, virtually eliminating any solvent loss during the experiment. Oscillation experiments were performed under a strain of 0.5% and in the frequency range from 0.1 to 10 Hz. In the plots, all the points that showed a torque different from the displacement (that is, Lissajous plot different from a straight line) were removed. The data, shown in Fig. 3a, were used to determine *G*′ (storage modulus) and *G*″ (loss modulus).

## Online content

Any methods, additional references, Nature Portfolio reporting summaries, source data, extended data, supplementary information, acknowledgements, peer review information; details of author contributions and competing interests; and statements of data and code availability are available at 10.1038/s41586-024-08288-x.

## Supplementary information

Supplementary Information Supplementary Information sections 1–12, including the experimental procedures and data (synthesis, fuelling and characterization), Figs. 1–20 and Table 1.

Supplementary Video 1 Contraction with *S* reagents (close up): close-up video of the first 20 h fuelled contraction of gel-**1** with (*S*,*S*)-**2** and (*S*)-**4**.

Supplementary Video 2 Contraction with *S* reagents (full): full video (0–160 h) of the fuelled contraction of gel-**1** with (*S*,*S*)-**2** and (*S*)-**4**.

Supplementary Video 3 Contraction with *R* reagents (close up): close-up video of the first 20 h fuelled contraction of gel-**1** with (*R*,*R*)-**2** and (*R*)-**4**.

Supplementary Video 4 Contraction with *R* reagents (full): full video (0–160 h) of the fuelled contraction of gel-**1** with (*R*,*R*)-**2** and (*R*)-**4**.

Supplementary Video 5 Achiral fuelling: treatment of gel-**1** with achiral fuel DIC and DMAP.

Supplementary Video 6 Fuelling of control gel: treatment of control gel-**1**-Me2 with (*S*,*S*)-**2** and (*S*)-**4**.

Supplementary Video 7 Expansion–contraction (close up): close-up video of the first 20 h fuelled expansion–contraction of the pre-contracted gel ((*S*,*S*)-**2** and (*S*)-**4**) treated with (*R*,*R*)-**2** and (*R*)-**4**.

Supplementary Video 8 Expansion–contraction (close up): full video (0–100 h) of the fuelled expansion–contraction of the pre-contracted gel ((*S*,*S*)-**2** and (*S*)-**4**) treated with (*R*,*R*)-**2** and (*R*)-**4**.

Supplementary Video 9 Expansion with achiral fuel: expansion of the pre-contracted gel ((*S*,*S*)-**2** and (*S*)-**4**) treated with achiral DIC and DMAP.

## Data Availability

The data that support the findings of this study are available in the Article and its Supplementary Information or are available from the Mendeley data repository (https://data.mendeley.com/) at 10.17632/b4tfyf522s.1.
