# Supplementary material for: Transducing chemical energy through catalysis by an artificial molecular motor
Source: Nature. 2025 Jan 15;637(8046):594–600. doi: 10.1038/s41586-024-08288-x (PMC11735380; doi:10.1038/s41586-024-08288-x)
Supplement: Supplementary file 1 — Supplementary Information sections 1–12, including the experimental procedures and data (synthesis, fuelling and characterization), Figs. 1–20 and Table 1. [file 41586_2024_8288_MOESM1_ESM.pdf]

nature portfolio

https://doi.org/10.1038/s41586-024-08288-x

Supplementary information

Transducing chemical energy through catalysis by an artificial molecular motor

In the format provided by the authors and unedited

Transducing chemical energy through catalysis by an artificial molecular motor

Peng-Lai Wang,[1,2] Stefan Borsley,[1] Martin J. Power,[1] Alessandro Cavasso,[3] Nicolas Giuseppone*[3,4] and David A. Leigh*[1,2]

Affiliations:

[1]Department of Chemistry, University of Manchester, Oxford Road, Manchester M13 9PL, UK

[2]School of Chemistry and Molecular Engineering, East China Normal University, 200062 Shanghai, China

[3]SAMS Research Group, Université de Strasbourg, Institut Charles Sadron – CNRS, 23 rue du Loess, BP 84047, 67034 Strasbourg Cedex 2, France

[4]Institut Universitaire de France (IUF), Paris 75005, France

*Corresponding authors. Email: david.leigh@manchester.ac.uk, giuseppone@unistra.fr

Supplemental Information – Experimental procedures and data (synthesis, fuelling, characterisation)

Contents

S1. General methods and abbreviations 2
S1.1 List of supplementary videos 3
S2. Synthesis and characterisation of small organic molecules 4
S2.1 Synthesis of motor **1** and **1**-Me_2 4
S2.2 Synthesis of chiral fuels and chiral hydrolysis promoters 19
S2.3 Synthesis of α,ω-diazido-poly(ethylene glycol) PEG_M 21
S3. Preparation of gel-**1** 23
S3.1 Synthetic procedure for formation of gel-**1** 23
S3.2 Optimisation of gelation conditions 25
S3.3 Optimisation of solvent conditions for gel swelling 26
S4. Evaluation of directional rotation 27
S4.1 Chemical fuelling of motor **1** 27
S4.2 Directional rotation of model motor **5** under unbuffered conditions 29
S4.3 ^{1}H NMR analysis of the catalytic efficacy of gel-**1** 29
S5. Gel contraction experiments 30
S5.1 Image recording 30
S5.2 Image analysis 30
S5.3 Contraction experiments with gel-**1** 31
S5.3.1 Gel contraction experiments with varied length of PEG_M 32
S5.4 Control gel contraction experiments 33
S5.4.1 Treatment of gel-**1** with only hydrolysis promoter (*S*)-**4** 33
S5.4.2 Treatment of gel-**1** with achiral DMAP and DIC 34
S5.4.3 Operation of non-rotating gel-**1**-Me_2 35
S5.5 Gel expansion–contraction experiments 36
S5.5.1 Expansion–contraction – treatment with reagents of opposite chirality 36
S5.5.2 Control – treatment with reagents of the same chirality 36
S5.5.3 Control – treatment with achiral reagents 37
S5.6 Thermal response of unfuelled and fuel-contracted gel-**1** 38
S6. Rheology and AFM experiments 39
S7. Tensile testing experiments 41
S8. Non-ideal gels and energy storage 42
S8.1 Characterisation of the non-ideal gel 42
S8.2 Quantification of motor rotation in gel and elastic energy storage 42
S9. NMR spectra 45
S10. HPLC traces 64
S11. Mass spectra 65
S12. References 66

S1. General methods and abbreviations

Unless stated otherwise, reagents were obtained from commercial sources and used without purification. Anhydrous solvents THF, DMF and CH_2Cl_2 were obtained by passing the solvent through an activated alumina column on a Phoenix SDS (solvent drying system; JC Meyer Solvent Systems, CA, USA). ^1H NMR spectra were recorded on a Bruker Avance III instrument with an Oxford AS600 magnet equipped with a cryoprobe [5mm CPDCH ^{13}C-^1H/D] (600 MHz). Chemical shifts are reported in parts per million (ppm) relative to tetramethylsilane from high to low frequency using the residual solvent peak as the internal reference ($CDCl_3$ = 7.26 ppm, CD_3CN = 1.94 ppm, 1,4-dioxane-d_8= 3.53 ppm). All ^1H resonances are reported to the nearest 0.01 ppm. The multiplicity of ^1H signals is indicated as: s = singlet; d = doublet; t = triplet; q = quartet; multiplet; br = broad; or combinations of thereof. Coupling constants (*J*) are quoted in Hz and reported to the nearest 0.1 Hz. Where appropriate, averages of the signals from peaks displaying multiplicity were used to calculate the value of the coupling constant. ^{13}C NMR spectra were recorded on the same spectrometer at 298 K with the central resonance of the solvent peak as the internal reference ($CDCl_3$ = 77.16 ppm, CD_3CN = 118.26 ppm). All ^{13}C resonances are reported to the nearest 0.1 ppm. DEPT, COSY, HSQC and HMBC experiments were used to aid structural determination and spectral assignment. Flash column chromatography was carried out using Silica 60 Å (particle size 40–63 µm, Sigma Aldrich, UK) as the stationary phase. Preparative TLC was performed using PLC 20 × 20 cm, 60 F254 preparatory plates of various thicknesses (250–2000 µm). Analytical TLC was performed on precoated silica gel plates (0.25 mm thick, 60 F254, Merck, Germany) and visualized using both short and long wave ultraviolet light in combination with standard laboratory stains (acidic potassium permanganate, iodine vapor). Infrared spectra were recorded neat on a Bruker FT-IR Alpha II Platinum ATR spectrometer. Low resolution ESI mass spectrometry was performed with a Thermo Scientific LCQ Fleet Ion Trap Mass Spectrometer or an Agilent Technologies 1200 LC system with an Advion Expression CMS L single quadrupole MS detector. High-resolution mass spectrometry (HRMS) was carried out at the Mass Spectrometry Service, Department of Chemistry, University of Manchester.

Abbreviations: DIC: diisopropylcarbodiimide; DIU: diisopropylurea; DMAP: 4-(dimethylamino)pyridine; DMF: dimethylformamide; DMSO: dimethylsulfoxide; EDTA:

ethylenediaminetetraacetic acid; ESI: electrospray ionisation; h: hour; HRMS: high-resolution mass spectrometry; MES buffer: 2-morpholinoethanesulfonic acid monohydrate buffer solution; NMR: nuclear magnetic resonance; PMDETA: *N*,*N*,*N*',*N*'',*N*''-pentamethyldiethylenetriamine; ppm: parts per million; TBAF: tetra-*n*-butylammonium fluoride; THF: tetrahydrofuran; TLC: thin layer chromatography; rt: room temperature.

S1.1 List of supplementary videos

Supplementary Video S1 | Contraction with *S*-reagents (close-up). Close-up video of first 20 h fuelled contraction of gel-**1** with (*S*,*S*)-**2** and (*S*)-**4**.

Supplementary Video S2 | Contraction with *S*-reagents (full). Full video (0−160 h) of fuelled contraction of gel-**1** with (*S*,*S*)-**2** and (*S*)-**4**.

Supplementary Video S3 | Contraction with *R*-reagents (close-up). Close-up video of first 20 h fuelled contraction of gel-**1** with (*R*,*R*)-**2** and (*R*)-**4**.

Supplementary Video S4 | Contraction with *R*-reagents (full). Full video (0−160 h) of fuelled contraction of gel-**1** with (*R*,*R*)-**2** and (*R*)-**4**.

Supplementary Video S5 | Achiral fuelling. Treatment of gel-**1** with achiral fuel DIC and DMAP.

Supplementary Video S6 | Fuelling of control gel. Treatment of control gel-**1**-Me_2 with (*S*,*S*)-**2** and (*S*)-**4**.

Supplementary Video S7 | Expansion–contraction (close-up). Close-up video of first 20 h fuelled expansion–contraction of pre-contracted gel ((*S*,*S*)-**2** and (*S*)-**4**) treated with (*R*,*R*)-**2** and (*R*)-**4**.

Supplementary Video S8 | Expansion–contraction (full). Full video (0–100 h) of fuelled expansion–contraction of pre-contracted gel ((*S*,*S*)-**2** and (*S*)-**4**) treated with (*R*,*R*)-**2** and (*R*)-**4**.

Supplementary Video S9 | Expansion with achiral fuel. Expansion of pre-contracted gel ((*S*,*S*)-**2** and (*S*)-**4**) treated with achiral DIC and DMAP.

S2. Synthesis and characterisation of small organic molecules

S2.1 Synthesis of motor 1 and 1-Me_2

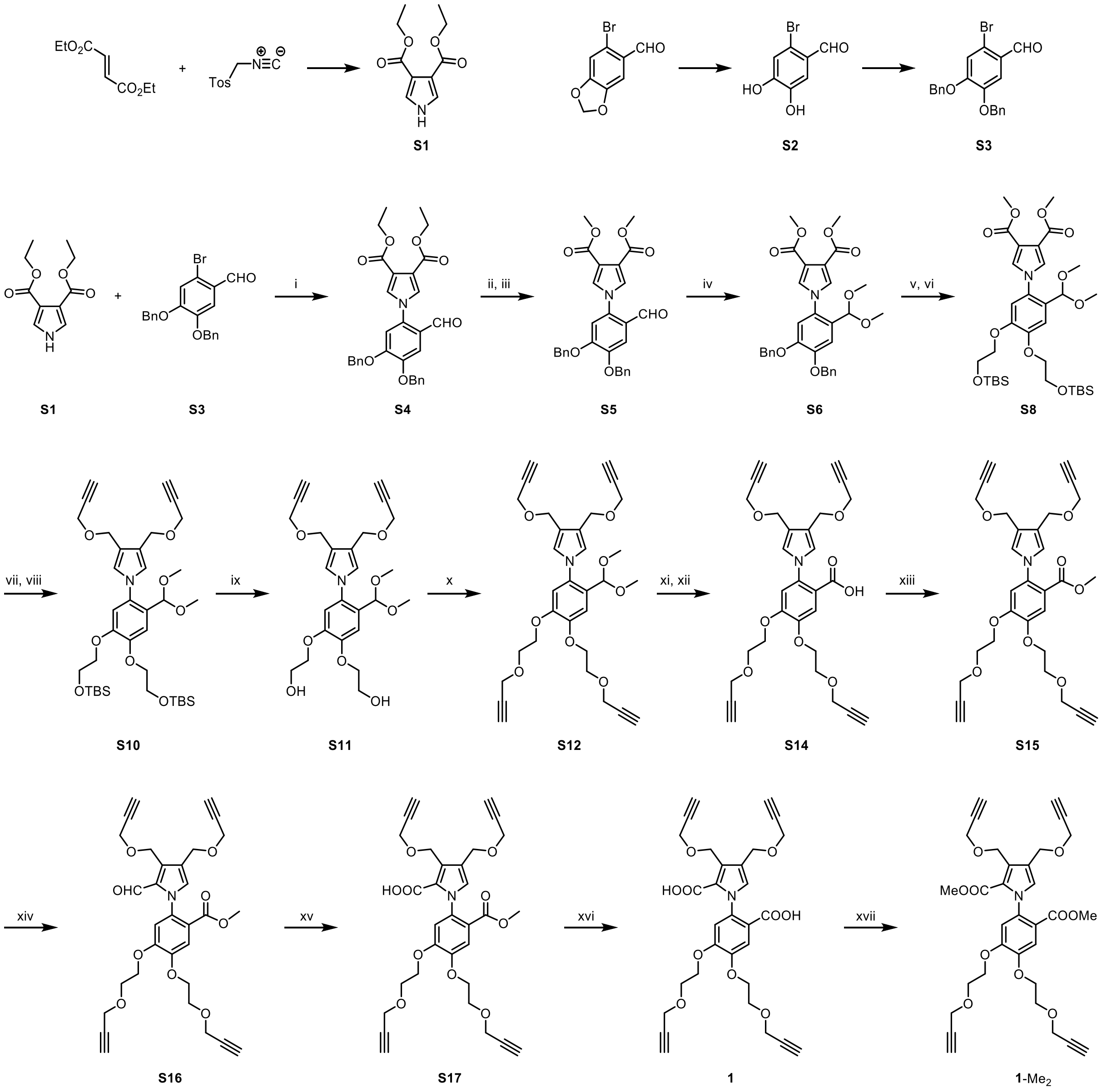

Scheme S1. Reagents and conditions for the synthesis of motor **1** and **1-**Me_2. Compounds **S1, S2** and **S3** were synthesised according to reported procedures.[S1,S2,S3] (i) CuI, K_2CO_3, *trans-N,N'*-dimethylcyclohexane-1,2-diamine, DMF, 120 °C, 30 h. (ii) NaOH, THF/EtOH/H_2O, 80 °C, 2 h. (iii) K_2CO_3, MeI, DMF, 70 °C, 2 h. (iv) *para*-toluenesulfonic acid monohydrate, trimethylorthoformate, dry MeOH, 68 °C, 2 h. (v) 10% Pd/C, MeOH, r.t., 4 h. (vi) K_2CO_3, KI, (2-bromoethoxy)-*tert*-butyldimethylsilane, DMF, 80 °C, 8 h. (vii) $LiAlH_4$, dry THF, 0 °C, 2 h. (viii) NaH, dry THF, 0 °C, 2 h, then propargyl bromide, r.t., 12 h. (ix) TBAF, THF, 0 °C, 0.5 h. (x) NaH, dry THF, 0 °C, 2 h, then propargyl bromide, r.t., 12 h. (xi) 1 M HCl, THF, r.t., 4 h. (xii) $NaClO_2$, NaH_2PO_4, 2-methylbut-2-ene, THF/*t*-BuOH/H_2O, r.t., 24 h. (xiii) K_2CO_3, MeI, DMF, r.t., 16 h. (xiv) $POCl_3$, DMF, 40 °C, 1 h. xv) $NaClO_2$, NaH_2PO_4, 2-methylbut-2-ene, THF/*t*-BuOH/H_2O, r.t., 24 h. (xvi) NaOH, THF/EtOH/H_2O, 70 °C, 2 h. (xvii) K_2CO_3, MeI, DMF, r.t., 16 h.

S4

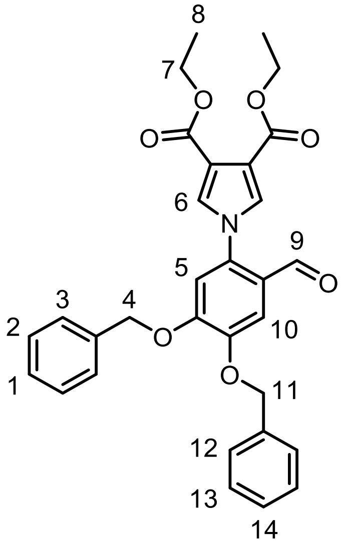

To a Schlenk tube were added CuI (9.5 mg, 0.05 mmol), **S1** (211 mg, 1.0 mmol), K_2CO_3 (290 mg, 2.1 mmol), **S3** (477 mg, 1.2 mmol) and a stirrer bar. The tube was fitted with a rubber septum, evacuated and backfilled with nitrogen three times. *Trans-N,N'*-dimethylcyclohexane-1,2-diamine (32 μL, 0.20 mmol) and dry DMF (1.0 mL) were sequentially added under nitrogen. The mixture was heated to 120 °C for 30 h. The mixture was diluted with EtOAc and washed with water and brine. The organic phase was dried over anhydrous sodium sulfate and concentrated under reduced pressure. The crude product was purified through flash column chromatography (SiO_2) eluting with petroleum ether/EtOAc (4:1→2:1, *v*/*v*) to afford **S4** (416 mg, 79%).

^{1}H NMR (600 MHz, CD_3CN) δ 9.60 (s, 1H, H_9), 7.53 (s, 1H, H_{10}), 7.49 (s, 2H, H_6), 7.47 – 7.33 (m, 10H, $H_{1,2,3,12,13,14}$), 7.17 (s, 1H, H_5), 5.22 (s, 2H, H_4), 5.20 (s, 2H, H_{11}), 4.25 (q, 4H, H_7), 1.30 (t, 6H, H_8).

^{13}C NMR (151 MHz, CD_3CN) δ 188.2, 164.1, 154.6, 149.7, 137.4, 137.2, 136.9, 130.6, 129.6, 129.5, 129.3, 129.1, 129.0, 128.8, 124.9, 117.9, 112.9, 112.1, 72.1, 71.6, 61.2, 14.5.

HRMS (ESI$^+$) calcd. For $C_{31}H_{29}NO_7Na$ $[M+Na]^+$ = 550.1842, found 550.1846.

S5

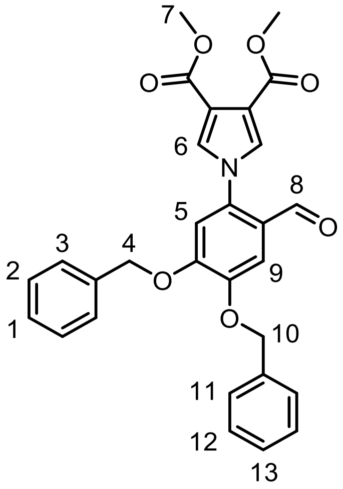

NaOH (128 mg, 3.2 mmol) was added to the stirred solution of compound **S4** (528 mg, 1.0 mmol) in a mixed solvent of THF, EtOH and H_2O (12 mL, 1:1:1 *v*/*v*/*v*). The solution was heated to 80 °C and stirred for 2 h. The mixture was cooled to room temperature, acidified to pH = 3 with 2 M HCl, and extracted with EtOAc. The organic phase was washed with brine, dried over anhydrous sodium sulfate and evaporated under reduced pressure. The crude product was dissolved in dry DMF (5 mL). To the solution were added K_2CO_3 (828 mg, 6 mmol) and iodomethane (250 µL, 4 mmol) under nitrogen. The mixture was heated to 70 °C for 2 h. The mixture was diluted with EtOAc and washed with water and brine. The organic phase was dried over anhydrous sodium sulfate and concentrated under reduced pressure. The crude product was purified through flash column chromatography (SiO_2) eluting with petroleum ether/EtOAc (4:1→2:1, *v*/*v*) to afford **S5** (475 mg, 95%).

^{1}H NMR (600 MHz, CD_3CN) δ 9.60 (s, 1H, H_8), 7.54 (s, 1H, H_9), 7.51 (s, 2H, H_6), 7.48 – 7.35 (m, 10H, $H_{1,2,3,11,12,13}$), 7.18 (s, 1H, H_5), 5.23 (s, 2H, H_4), 5.21 (s, 2H, H_{10}), 3.78 (s, 6H, H_7).

^{13}C NMR (151 MHz, CD_3CN) δ 188.3, 164.4, 154.6, 149.7, 137.4, 137.1, 136.9, 130.8, 129.6, 129.5, 129.4, 129.2, 129.0, 128.8, 125.0, 117.4, 112.9, 112.1, 72.1, 71.7, 52.1.

HRMS (ESI^+) calcd. For $C_{29}H_{25}NO_7Na$ $[M+Na]^+$ = 522.1529, found 522.1534.

S6

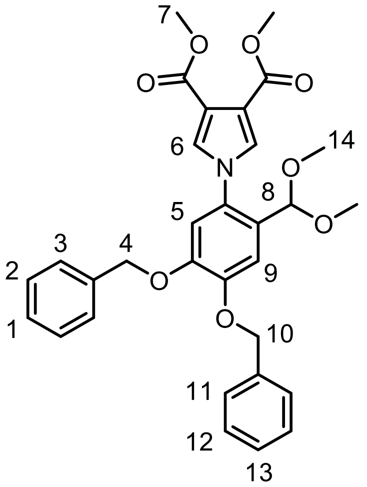

To a flask were added **S5** (1.5 g, 3.0 mmol), *p*-toluenesulfonic acid (45 mg, cat.) and trimethylorthoformate (3 mL) under nitrogen. Dry MeOH (30 mL) was injected. The mixture was refluxed at 68 °C for 2 hours. After cooling, 40 ml EtOAc was added and then washed with sat. $NaHCO_3$ and water. The aqueous layer was extracted with EtOAc. The combined organic solution was washed with brine and dried over anhydrous sodium sulfate and concentrated under reduced pressure. The crude product was purified through flash column chromatography (SiO_2) eluting with petroleum ether/EtOAc (2:1, *v*/*v*) to afford **S6** (1.6 g, 98%).

^{1}H NMR (600 MHz, CD_3CN) δ 7.46 – 7.32 (m, 10H, $H_{1,2,3,11,12,13}$), 7.38 (s, 2H, H_6), 7.25 (s, 1H, H_9), 7.04 (s, 1H, H_5), 5.19 (s, 2H, H_{10}), 5.11 (s, 2H, H_4), 4.93 (s, 1H, H_8), 3.78 (s, 6H, H_7), 3.18 (s, 6H, H_{14}).

^{13}C NMR (151 MHz, CD_3CN) δ 164.6, 149.7, 149.3, 137.9, 137.6, 131.7, 130.5, 129.5, 129.45, 129.1, 129.0, 128.9, 128.8, 127.4, 116.7, 113.7, 113.1, 100.7, 71.8, 71.6, 54.0, 52.0.

HRMS (ESI$^+$) calcd. For $C_{31}H_{31}NO_8Na$ $[M+Na]^+$ = 568.1947, found 568.1954.

S8

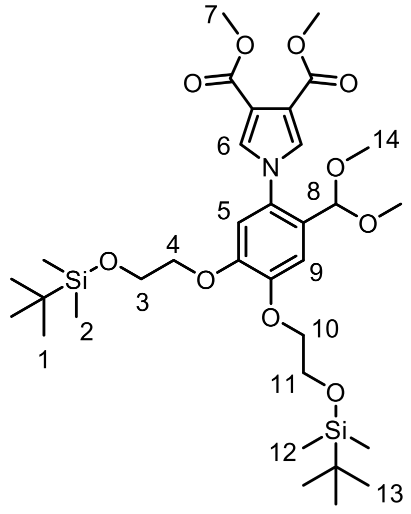

To a sealed solution of **S6** (1.1 g, 2.0 mmol, 1.0 eq.) in MeOH (50 mL) was added 10% Pd/C (400 mg, 0.1 eq.), the reaction vessel was then evacuated and backfilled three times with 1 atm H_2 and stirred under a balloon of H_2 for 4 hours at room temperature. The mixture was filtered over celite and concentrated under reduced pressure to give the crude acid **S7** (assumed quant., 2.0 mmol), which was used without further purification.

To **S7** (assumed 2.0 mmol) in dry DMF (6 mL) were added K_2CO_3 (1.1 g, 8.0 mmol), KI (1.33 g, 8.0 mmol). To the mixture was added the solution of (2-bromoethoxy)-*tert*-butyldimethylsilane (1.91 g, 8.0 mmol) in dry DMF (4 mL) at room temperature and the suspension was stirred at 80 °C for 8 hours. The mixture was diluted with EtOAc and washed with water and brine. The organic phase was dried over anhydrous sodium sulfate and concentrated under reduced pressure. The crude product was purified through flash column chromatography (SiO_2) eluting with petroleum ether/EtOAc (4:1→3:1, *v*/*v*) to afford **S8** (906 mg, 66.4%).

^{1}H NMR (600 MHz, CD_3CN) δ 7.39 (s, 2H, H_6), 7.16 (s, 1H, H_9), 6.96 (s, 1H, H_5), 4.96 (s, 1H, H_8), 4.10 (t, *J* = 5.0 Hz, 2H, H_{10}), 4.06 (t, *J* = 5.0 Hz, 2H, H_4), 3.97 (t, *J* = 5.0 Hz, 2H, H_{11}), 3.95 (t, *J* = 5.0 Hz, 2H, H_3), 3.78 (s, 6H, H_7), 3.21 (s, 6H, H_{14}), 0.90 (s, 9H, H_{13}), 0.87 (s, 9H, H_1), 0.10 (s, 6H, H_{12}), 0.07 (s, 6H, H_2).

^{13}C NMR (151 MHz, CD_3CN) δ 164.6, 149.9, 149.5, 131.6, 130.6, 127.1, 116.6, 113.2, 112.4, 100.6, 71.7, 71.6, 62.8, 62.7, 53.9, 51.9, 26.3, 26.2, 18.9, 18.8, −5.1, −5.2.

HRMS (ESI$^+$) calcd. For $C_{33}H_{55}NO_{10}Si_2Na$ $[M+Na]^+$ = 704.3262, found 704.3285.

S10

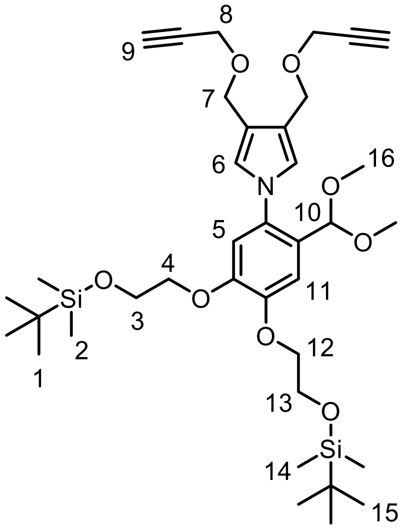

To a flask was added **S8** (1.3 g, 1.9 mmol) and dry THF (20 mL) under nitrogen. After the solution was cooled to 0 °C in an ice bath, $LiAlH_4$ (2.5 mL, 2.4 M in THF) was added to the solution dropwise. The mixture was stirred for 2 hours at 0 °C. After diluting with Et_2O and ensuring the temperature remained at 0 °C, to the mixture was sequentially slowly added 0.23 mL H_2O, 0.23 mL aqueous 15% NaOH solution, and 0.69 mL H_2O. The mixture was warmed to room temperature and stirred for 15 min, then dried over anhydrous magnesium sulfate. The solvent was removed to give the desired product **S9** (assumed quant., 1.9 mmol), which was used without further purification.

To a flask under N_2 was added compound **S9** (assumed, 1.9 mmol) and dry THF (16 mL). NaH (300 mg, 7.4 mmol, 60% in mineral oil) was added to the solution at 0 °C and the mixture was let stir at room temperature for 2 h. Propargyl bromide (0.80 mL, 80 wt. % in toluene) was added and the mixture was stirred for 12 h. The mixture was quenched with saturated NH_4Cl solution and extracted with EtOAc. The organic phase was washed with brine, dried over anhydrous sodium sulfate and concentrated under reduced pressure. The crude product was purified through flash column chromatography (SiO_2), eluting with petroleum ether/EtOAc (6:1→4:1, *v*/*v*) to give the desired product **S10** (1.15 g, 86%).

^{1}H NMR (600 MHz, CD_3CN) δ 7.12 (s, 1H, H_{11}), 6.86 (s, 1H, H_5), 6.84 (s, 2H, H_6), 4.89 (s, 1H, H_{10}), 4.50 (s, 4H, H_7), 4.14 (d, J = 2.5 Hz, 4H, H_8), 4.08 (t, J = 4.9 Hz, 2H, H_{12}), 4.06 (t, J = 4.8 Hz, 2H, H_4), 3.96 (t, J = 4.9 Hz, 2H, H_{13}), 3.94 (t, J = 4.8 Hz, 2H, H_3),

3.21 (s, 6H, H_{16}), 2.70 (t, *J* = 2.5 Hz, 2H, H_9), 0.90 (s, 9H, H_{15}), 0.87 (s, 9H, H_1), 0.10 (s, 6H, H_{14}), 0.07 (s, 6H, H_2).

^{13}C NMR (151 MHz, CD_3CN) δ 149.9, 148.8, 133.6, 127.1, 124.6, 120.4, 112.9, 112.4, 101.3, 81.2, 75.4, 71.6, 71.5, 63.9, 62.8, 62.7, 56.9, 54.3, 26.2, 26.1, 18.9, 18.8, −5.1, −5.2.

HRMS (ESI$^+$) calcd. For $C_{37}H_{59}NO_8Si_2Na$ $[M+Na]^+$ = 724.3677, found 724.3701.

S11

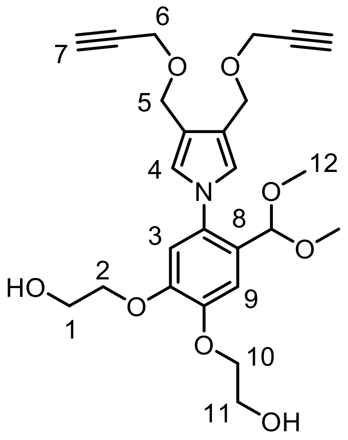

To a solution of **S10** (421 mg, 0.60 mmol) in THF (15 mL) was added TBAF (1.32 mL, 1M in THF, 1.32 mmol) at 0 °C and the mixture was stirred at 0 °C for 30 min. Then a saturated solution of NH_4Cl (10 mL) was added and the mixture was extracted with CH_2Cl_2 (3×20 mL). The combined organic layers were dried over Na_2SO_4 and concentrated under reduced pressure. The crude product was purified through flash column chromatography (SiO_2), eluting with CH_2Cl_2/MeOH (20:1, *v*/*v*) to give the desired product **S11** (270 mg, 95%).

^{1}H NMR (600 MHz, CD_3CN) δ 7.15 (s, 1H, H_9), 6.88 (s, 1H, H_3), 6.85 (s, 2H, H_4), 4.89 (s, 1H, H_8), 4.50 (s, 4H, H_5), 4.14 (d, *J* = 2.5 Hz, 4H, H_6), 4.10 (t, *J* = 4.4 Hz, 2H, H_{10}), 4.06 (t, *J* = 4.4 Hz, 2H, H_2), 3.82 – 3.79 (m, 4H, $H_{1,11}$), 3.22 (s, 6H, H_{12}), 2.71 (t, *J* = 2.5 Hz, 2H, H_7).

^{13}C NMR (151 MHz, CD_3CN) δ 150.1, 149.0, 133.9, 127.5, 124.6, 120.5, 113.4, 113.2, 101.3, 81.2, 75.4, 72.2, 72.0, 63.9, 61.2, 61.1, 56.9, 54.4.

HRMS (ESI$^+$) calcd. For $C_{25}H_{31}NO_8Na$ $[M+Na]^+$ = 496.1947, found 496.1952.

S12

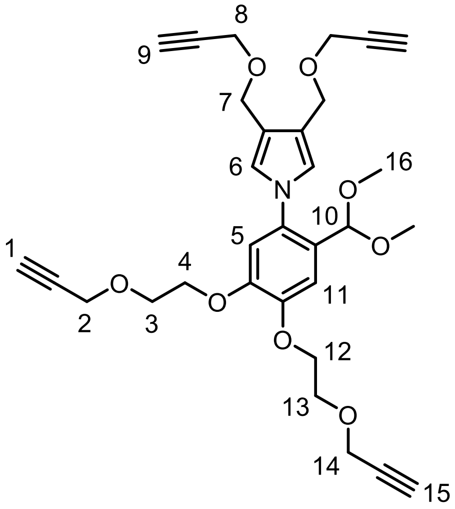

To a flask under N_2 was added compound **S11** (710 mg, 1.5 mmol) and dry THF (15 mL). NaH (180 mg, 4.5 mmol, 60% in mineral oil) was added to the solution at 0 °C and the mixture was stirred at room temperature for 2 h. Propargyl bromide (0.65 mL, 80 wt. % in toluene) was added and the mixture was stirred for 12 h. The mixture was quenched with saturated NH_4Cl solution and extracted with EtOAc. The organic phase was washed with brine, dried over anhydrous sodium sulfate and concentrated under reduced pressure. The crude product was purified through flash column chromatography (SiO_2) eluting with petroleum ether/EtOAc (4:1→2:1, *v*/*v*) to give the desired product **S12** (635 mg, 77%).

^{1}H NMR (600 MHz, CD_3CN) δ 7.13 (s, 1H, H_{11}), 6.86 (s, 1H, H_5), 6.85 (s, 2H, H_6), 4.89 (s, 1H, H_{10}), 4.50 (s, 4H, H_7), 4.25 (d, *J* = 2.5 Hz, 2H, H_{14}), 4.23 (d, *J* = 2.5 Hz, 2H, H_2), 4.19 (t, *J* = 4.5 Hz, 2H, H_{12}), 4.13 – 4.16 (m, 2H, H_4), 4.14 (d, *J* = 2.5 Hz, 4H, H_8), 3.84 (t, *J* = 4.5 Hz, 2H, H_{13}), 3.82 (t, *J* = 4.5 Hz, 2H, H_3), 3.22 (s, 6H, H_{16}), 2.73 (t, *J* = 2.5 Hz, 1H, H_{15}), 2.72 (t, *J* = 2.5 Hz, 1H, H_1), 2.70 (t, *J* = 2.5 Hz, 2H, H_9).

^{13}C NMR (151 MHz, CD_3CN) δ 149.6, 148.6, 133.7, 127.3, 124.6, 120.5, 112.9, 112.5, 101.3, 81.2, 80.8, 80.7, 75.9, 75.8, 75.4, 69.5, 69.4, 68.9, 68.8, 63.9, 58.9, 58.8, 56.9, 54.4.

HRMS (ESI$^+$) calcd. For $C_{31}H_{35}NO_8Na$ $[M+Na]^+$ = 572.2260, found 572.2269.

S13

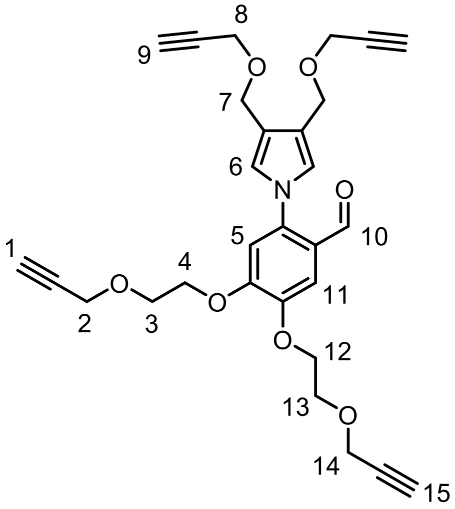

To a solution of **S12** (934 mg, 1.7 mmol) in THF (25 mL) was added 1 M HCl (10 mL) and the mixture was stirred for 4 hours at room temperature. Then the mixture was extracted with CH_2Cl_2 (3 × 20 mL). The combined organic layers were washed with saturated aqueous $NaHCO_3$ solution and brine, then dried over Na_2SO_4 and concentrated under reduced pressure to give the crude product **S13**, which was used without further purification.

^{1}H NMR (600 MHz, CD_3CN) δ 9.61 (s, 1H, H_{10}), 7.41 (s, 1H, H_{11}), 7.00 (s, 1H, H_5), 6.99 (s, 2H, H_6), 4.51 (s, 4H, H_7), 4.27 – 4.23 (m, 8H, $H_{2,4,12,14}$), 4.15 (d, *J* = 2.4 Hz, 4H, H_8), 3.87 – 3.85 (m, 4H, $H_{3,13}$), 2.74 – 2.73 (m, 2H, $H_{1,15}$), 2.71 (t, *J* = 2.4 Hz, 2H, H_9).

^{13}C NMR (151 MHz, CD_3CN) δ 188.9, 154.6, 148.8, 139.6, 125.4, 124.6, 121.6, 111.8, 111.1, 81.2, 80.7, 80.6, 75.9, 75.8, 75.5, 69.7, 69.5, 68.7, 68.5, 63.8, 58.9, 58.9, 57.1.

HRMS (ESI$^+$) calcd. For $C_{29}H_{29}NO_7Na$ $[M+Na]^+$ = 526.1842, found 526.1851.

S14

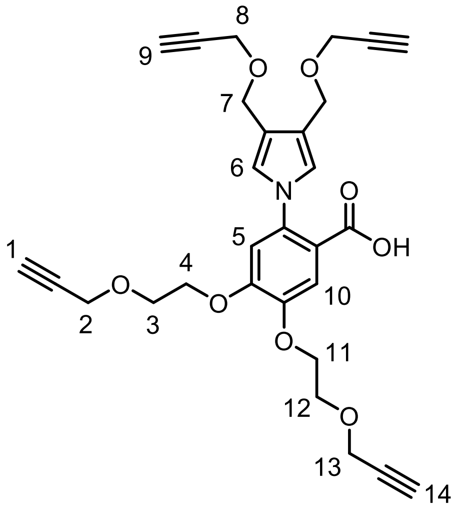

To a solution of aldehyde **S13** (856 mg, 1.7 mmol) in THF (38 mL) and *t*-BuOH (38 mL) was added 2-methylbut-2-ene (13 mL) at room temperature. Subsequently, a solution of $NaClO_2$ (461 mg, 5.1 mmol) and NaH_2PO_4 (612 mg, 5.1 mmol) in water (11 mL) was added dropwise to the solution and the mixture was stirred vigorously for 24 h at room temperature. The mixture was then diluted with saturated aqueous NH_4Cl and extracted with ethyl acetate. The combined organic extracts were washed with brine, dried over Na_2SO_4, filtered, and concentrated under reduced pressure to give the crude product **S14**, which was used without further purification.

^{1}H NMR (600 MHz, CD_3CN) δ 7.41 (s, 1H, H_{10}), 6.91 (s, 1H, H_5), 6.80 (s, 2H, H_6), 4.48 (s, 4H, H_7), 4.25 (d, J = 2.5 Hz, 2H, H_{13}), 4.23 (d, J = 2.5 Hz, 2H, H_2), 4.22 – 4.20 (m, 4H, $H_{4,11}$), 4.12 (d, J = 2.4 Hz, 4H, H_8), 3.85 – 3.83 (m, 4H, $H_{3,12}$), 2.74 – 2.72 (m, 2H, $H_{1,14}$), 2.70 (t, J = 2.4 Hz, 2H, H_9).

^{13}C NMR (151 MHz, CD_3CN) δ 166.9, 152.5, 147.9, 135.6, 124.0, 120.5, 119.6, 116.2, 113.1, 81.3, 80.7, 80.6, 75.9, 75.8, 75.3, 69.6, 69.5, 68.8, 68.6, 63.8, 58.9, 56.8.

HRMS (ESI$^+$) calcd. For $C_{29}H_{29}NO_8Na$ $[M+Na]^+$ = 542.1785, found 542.1778.

S15

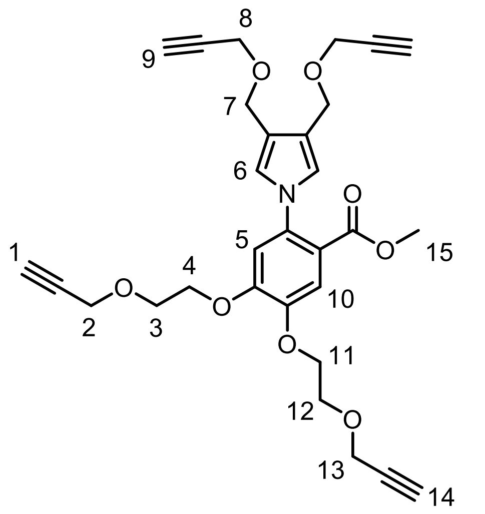

S14 (883 mg, 1.7 mmol) was dissolved in dry DMF (9 mL). To the solution were added K_2CO_3 (704 mg, 5.1 mmol) and iodomethane (212 µL, 3.4 mmol) under nitrogen. The mixture was stirred at room temperature for 16 hours. The mixture was diluted with EtOAc and washed with water and brine. The organic phase was dried over anhydrous sodium sulfate and evaporated under reduced pressure. The crude product was purified through flash column chromatography (SiO_2) eluting with petroleum ether/EtOAc (1:1, *v*/*v*) to afford **S15** (744 mg, 82%).

^{1}H NMR (600 MHz, CD_3CN) δ 7.37 (s, 1H, H_{10}), 6.93 (s, 1H, H_5), 6.78 (s, 2H, H_6), 4.48 (s, 4H, H_7), 4.25 (d, *J* = 2.5 Hz, 2H, H_{13}), 4.24 (d, *J* = 2.5 Hz, 2H, H_2), 4.22 – 4.20 (m, 4H, $H_{4,11}$), 4.12 (d, *J* = 2.4 Hz, 4H, H_8), 3.86 – 3.83 (m, 4H, $H_{3,12}$), 3.67 (s, 3H, H_{15}), 2.74 – 2.72 (m, 2H, $H_{1,14}$), 2.70 (t, *J* = 2.4 Hz, 2H, H_9).

^{13}C NMR (151 MHz, CD_3CN) δ 167.0, 152.5, 147.9, 135.4, 123.9, 120.6, 119.9, 115.9, 112.9, 81.2, 80.7, 80.6, 75.9, 75.8, 75.4, 69.7, 69.5, 68.8, 68.6, 63.8, 58.9, 56.8, 52.6.

HRMS (ESI$^+$) calcd. For $C_{30}H_{31}NO_8Na$ $[M+Na]^+$ = 556.1947, found 556.1966.

S16

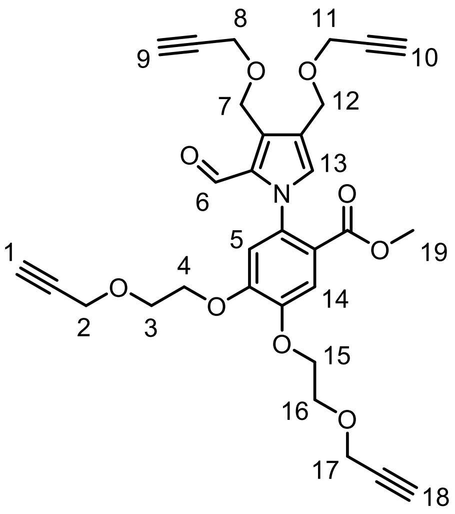

$POCl_3$ (0.43 mL, 4.63 mmol) was added dropwise to dry DMF (9 mL) in a flask under nitrogen atmosphere and the resulting mixture was allowed to stir for 15 min at 0 °C. To this mixture was then added **S15** (240 mg, 0.45 mmol) in dry DMF (2 mL). The mixture was stirred at 40 °C for an additional hour. After cooling to room temperature, the reaction mixture was diluted with water and hydrolysis was completed by adding a saturated aqueous sodium acetate solution. The mixture was cooled and extracted with EtOAc, and the combined organic layer was washed successively with water and brine. The organic phase was dried over anhydrous sodium sulfate and concentrated under reduced pressure. The crude product was purified through flash column chromatography (SiO_2), eluting with petroleum ether/EtOAc (3:2, *v*/*v*) to afford **S16** (222 mg, 88%).

^{1}H NMR (600 MHz, CD_3CN) δ 9.59 (s, 1H, H_6), 7.53 (s, 1H, H_{14}), 6.99 (s, 1H, H_{13}), 6.96 (s, 1H, H_5), 4.82 (d, J = 2.2 Hz, 2H, H_7), 4.54 (s, 2H, H_{12}), 4.26 (d, J = 2.4 Hz, 2H, H_{17}), 4.24 (t, J = 4.4 Hz, 2H, H_{15}), 4.23 (d, J = 2.4 Hz, 2H, H_2), 4.20 (d, J = 2.4 Hz, 2H, H_8), 4.19 – 4.17 (m, 2H, H_4), 4.17 (d, J = 2.4 Hz, 2H, H_{11}), 3.87(t, J = 4.4 Hz, 2H, H_{16}), 3.82 (t, J = 4.4 Hz, 2H, H_3), 3.60 (s, 3H, H_{19}), 2.76 (t, J = 2.4 Hz, 1H, H_9), 2.75 – 2.73 (m, 2H, $H_{10,18}$), 2.72 (t, J = 2.4 Hz, 1H, H_1).

^{13}C NMR (151 MHz, CD_3CN) δ 179.9, 165.5, 152.4, 148.8, 134.2, 131.6, 131.6, 130.7, 122.2, 121.3, 115.6, 115.0, 80.9, 80.9, 80.7, 80.6, 75.9, 75.9, 75.9, 75.8, 69.6, 69.5, 68.8, 68.6, 63.1, 61.6, 58.9, 58.9, 57.6, 57.3, 52.5.

HRMS (ESI$^+$) calcd. For $C_{31}H_{31}NO_9Na$ $[M+Na]^+$ = 584.1897, found 584.1913.

S17

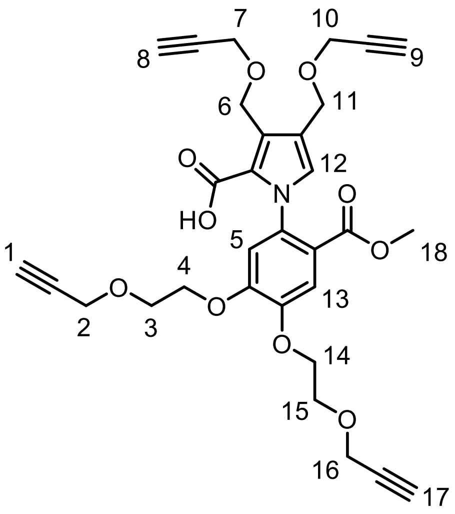

To a solution of aldehyde **S16** (1.35 g, 2.4 mmol) in THF (45 mL) and *t*-BuOH (45 mL) was added 2-methylbut-2-ene (21 mL) at room temperature. Subsequently, a solution of $NaClO_2$ (651 mg, 7.2 mmol) and NaH_2PO_4 (864 mg, 7.2 mmol) in water (16 mL) was added dropwise to the solution and the mixture was stirred vigorously for 24 h at room temperature. The mixture was then diluted with saturated aqueous NH_4Cl and extracted with ethyl acetate. The combined organic extracts were washed with brine, dried over Na_2SO_4, filtered, and concentrated under reduced pressure. The crude product was purified through flash column chromatography (SiO_2), eluting with petroleum ether/EtOAc (2:1→0:1, *v*/*v*) to afford **S17** (1.26 g, 91%).

^{1}H NMR (600 MHz, CD_3CN) δ 7.50 (s, 1H, H_{13}), 6.90 (s, 1H, H_5), 6.88 (s, 1H, H_{12}), 4.83 – 4.79 (m, 2H, H_6), 4.52 (s, 2H, H_{11}), 4.26 (d, *J* = 2.5 Hz, 2H, H_{16}), 4.24 – 4.22 (m, 2H, H_{14}), 4.22 (d, *J* = 2.4 Hz, 2H, H_2), 4.20 (d, *J* = 2.4 Hz, 2H, H_7), 4.17 – 4.15 (m, 4H, $H_{4,10}$), 3.86 (t, *J* = 4.3 Hz, 2H, H_{15}), 3.83 – 3.81(m, 2H, H_3), 3.60 (s, 3H, H_{18}), 2.74 – 2.72 (m, 4H, $H_{1,8,9,17}$).

^{13}C NMR (151 MHz, CD_3CN) δ 165.7, 161.4, 152.3, 148.4, 136.0, 129.9, 127.2, 124.0, 121.5, 121.2, 115.5, 114.9, 81.0, 81.0, 80.7, 80.6, 75.9, 75.8, 75.7, 75.7 (2 carbon signals overlapping), 69.6, 69.5, 68.8, 68.6, 63.4, 63.1, 58.9, 58.9 (2 carbon signals overlapping), 57.7, 57.2, 52.4.

HRMS (ESI$^+$) calcd. For $C_{31}H_{31}NO_{10}Na$ $[M+Na]^+$ = 600.1846, found 600.1841.

1

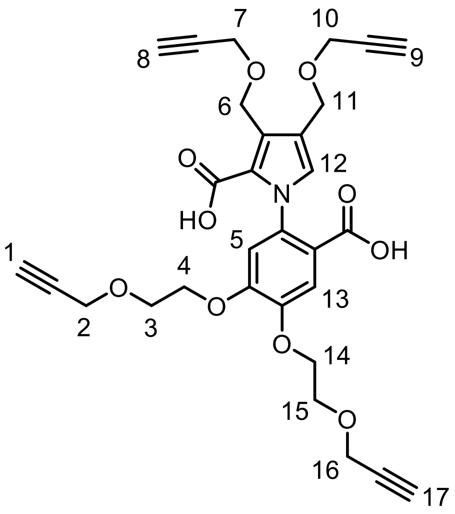

NaOH (200 mg, 5.0 mmol) was added to the stirred solution of compound **S17** (578 mg, 1.0 mmol) in a mixed solvent of THF, ethanol and water (12 mL, 1:1:1 *v*/*v*/*v*). The solution was heated to 70 °C and stirred for 2 h. The mixture was cooled to room temperature, acidified to pH = 3 with 2 M HCl, and extracted with EtOAc. The organic phase was washed with brine, dried over anhydrous sodium sulfate and concentrated under reduced pressure. The crude product was purified through flash column chromatography (SiO_2), eluting with petroleum ether/EtOAc (1:1→0:1, *v*/*v*) to afford **1** (507 mg, 90%).

^{1}H NMR (600 MHz, CD_3CN) δ 7.50 (s, 1H, H_{13}), 6.88 (s, 1H, H_{12}), 6.87 (s, 1H, H_5), 4.81 (s, 2H, H_6), 4.51 (s, 2H, H_{11}), 4.26 (d, *J* = 2.5 Hz, 2H, H_{16}), 4.24 – 4.22 (m, 4H, $H_{2,14}$), 4.18 (d, *J* = 2.4 Hz, 2H, H_7), 4.17 – 4.14 (m, 4H, $H_{4,10}$), 3.86 (t, *J* = 4.3 Hz, 2H, H_{15}), 3.83 – 3.81(m, 2H, H_3), 2.74 – 2.72 (m, 4H, $H_{1,8,9,17}$).

^{13}C NMR (151 MHz, CD_3CN) δ 165.7, 161.4, 152.3, 148.4, 136.0, 129.9, 127.2, 124.0, 121.5, 121.2, 115.5, 114.9, 81.0, 81.0, 80.7, 80.6, 75.9, 75.8, 75.7, 75.7 (2 carbon signals overlapping), 69.6, 69.5, 68.8, 68.6, 63.4, 63.1, 58.9, 58.9 (2 carbon signals overlapping), 57.7, 57.2, 52.4.

HRMS (ESI$^+$) calcd. For $C_{30}H_{29}NO_{10}Na$ $[M+Na]^+$ = 586.1684, found 586.1672.

1-Me$_2$

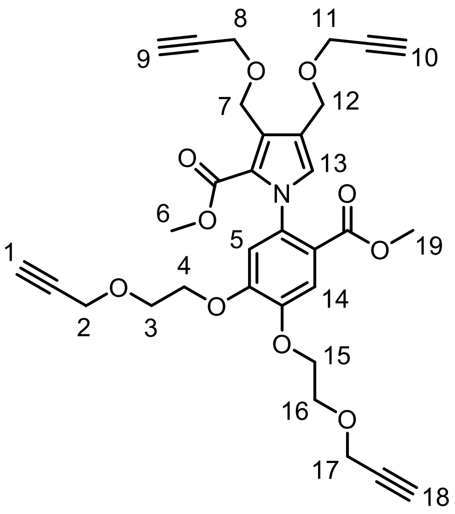

1 (28 mg, 0.05 mmol) was dissolved in dry DMF (1 mL). To the solution were added K_2CO_3 (41 mg, 0.30 mmol) and iodomethane (12.5 μL, 0.20 mmol) under nitrogen. The mixture was stirred at room temperature for 16 hours. The mixture was diluted with EtOAc and washed with water and brine. The organic phase was dried over anhydrous sodium sulfate and concentrated under reduced pressure. The crude product was purified through flash column chromatography (SiO_2), eluting with petroleum ether/EtOAc (1.5:1→1:1, *v*/*v*) to afford **2** (27 mg, 91%).

^{1}H NMR (600 MHz, CD_3CN) δ 7.50 (s, 1H, H_{14}), 6.90 (s, 1H, H_5), 6.89 (s, 1H, H_{13}), 4.82 – 4.78 (m, 2H, H_7), 4.52 (s, 2H, H_{12}), 4.26 (d, *J* = 2.4 Hz, 2H, H_{17}), 4.25 – 4.22 (m, 4H, $H_{2,15}$), 4.17 – 4.15 (m, 6H, $H_{4,8,11}$), 3.86 (t, *J* = 4.3 Hz, 2H, H_{16}), 3.83 – 3.82 (m, 2H, H_3), 3.60 (s, 3H, H_{19}), 3.59 (s, 3H, H_6), 2.74 (t, *J* = 2.4 Hz, 2H, H_{18}), 2.75 – 2.72 (m, 4H, $H_{1,9,10}$).

^{13}C NMR (151 MHz, CD_3CN) δ 165.7, 161.7, 152.3, 148.4, 136.0, 129.7, 127.2, 124.1, 121.6, 121.2, 115.5, 114.8, 81.3, 81.0, 80.7, 80.6, 75.9, 75.8, 75.6, 75.3, 69.6, 69.5, 68.8, 68.6, 63.4, 62.9, 60.9, 58.9, 57.5, 57.2, 52.4, 51.5.

HRMS (ESI$^+$) calcd. For $C_{30}H_{30}NO_{10}$ $[M+H]^+$ = 591.2104, found 591.2109.

S2.2 Synthesis of chiral fuels and chiral hydrolysis promoters

Chiral hydrolysis promoters (*S*)-**4** and (*R*)-**4** were synthesised according to reported procedures. All spectral data were in agreement with reported values.[S4]

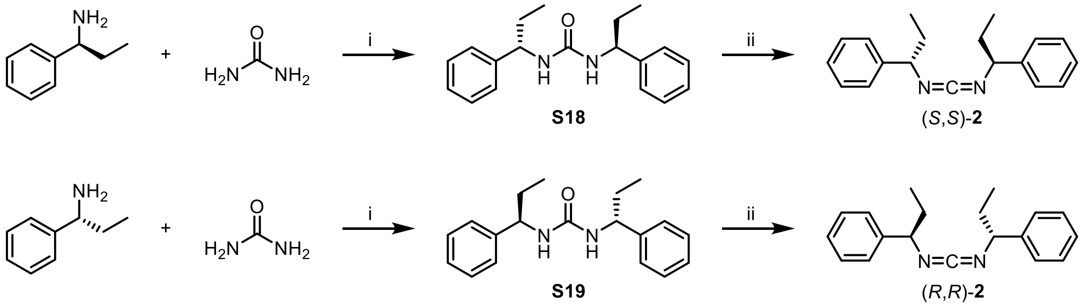

Scheme S2. Reagents and conditions: (i) *n*-butanol, 130 °C, 5 days. ii) PPh_3, CCl_4, TEA, CH_2Cl_2, 40 °C, 8 h.

S18

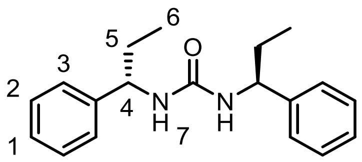

Urea (870 mg, 14.5 mmol) and (*S*)-1-phenylpropan-1-amine (5.0 mL, 34.8 mmol) were added to 10 mL butanol. The reaction mixture was refluxed for 5 days. After cooling down to room temperature, the mixture was diluted with EtOAc and washed with water and brine. The organic phase was dried over anhydrous sodium sulfate and concentrated under reduced pressure. The crude product was purified through flash column chromatography (SiO_2), eluting with petroleum ether/EtOAc (6:1→2:1, *v*/*v*) to afford **S18** (2.45 g, 57%).

^{1}H NMR (600 MHz, CD_3CN) δ 7.32 – 7.21 (m, 10H, $H_{1,2,3}$), 5.40 (m, 2H, H_7), 4.51 (m, 2H, H_4), 1.68 – 1.61 (m, 4H, H_5), 0.80 (t, *J* = 7.4 Hz, 6H, H_6),

^{13}C NMR (151 MHz, CD_3CN) δ 158.1, 145.6, 129.2, 127.5, 127.2, 56.4, 30.9, 11.0.

HRMS (ESI^+) calcd. For $C_{19}H_{24}N_2ONa$ $[M+Na]^+$ = 319.1781, found 319.1783.

(S,S)-**2**

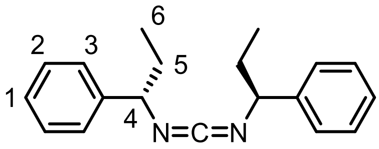

S18 (2.1 g, 7.1 mmol) and triphenylphosphine (2.22 g, 8.4 mmol) were suspended in 5.0 mL of dry CH_2Cl_2. CCl_4 (688 μL, 7.1 mmol) and Et_3N (990 μL, 7.1 mmol) were added and the reaction mixture was heated to reflux for 8 h. The solvent was removed under reduced pressure, and the crude product was purified through flash column chromatography (SiO_2), eluting with CH_2Cl_2 to afford (S,S)-**2** (1.40 g, 61%). Enantiomeric excess was determined by HPLC (Chiralpak IA), *n*-hexane/*i*-propanol =99:1, 25 °C, 210 nm, 1.0 mL/min, retention times: t_R (minor) 5.31 min, t_R (major) 5.61 min, ee = 99%.

^{1}H NMR (600 MHz, CD_3CN) δ 7.33 – 7.22 (m, 10H, $H_{1,2,3}$), 4.30 (t, J = 7.0 Hz, 2H, H_4), 1.77 – 1.66 (m, 4H, H_5), 0.83 (t, J = 7.3 Hz, 6H, H_6),

^{13}C NMR (151 MHz, CD_3CN) δ 143.8, 140.5, 129.3, 128.3, 127.4, 63.7, 32.4, 11.2.

HRMS (ESI^+) calcd. For $C_{19}H_{23}N_2$ $[M+H]^+$ = 279.1856, found 279.1860.

The synthesis of (R,R)-**2** was performed similarly to that of (S,S)-**2**, but with reagents of the opposite handedness.

S19

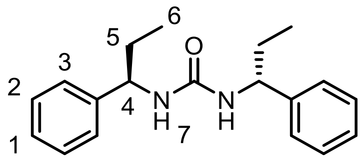

^{1}H NMR (600 MHz, CD_3CN) δ 7.32 – 7.21 (m, 10H, $H_{1,2,3}$), 5.28 (m, 2H, H_7), 4.50 (m, 2H, H_4), 1.69 – 1.61 (m, 4H, H_5), 0.81 (t, J = 7.4 Hz, 6H, H_6).

^{13}C NMR (151 MHz, CD_3CN) δ 158.0, 145.7, 129.2, 127.5, 127.1, 56.4, 30.9, 11.0.

HRMS (ESI^+) calcd. For $C_{19}H_{24}N_2ONa$ $[M+Na]^+$ = 319.1781, found 319.1779.

(*R*,*R*)-**2**

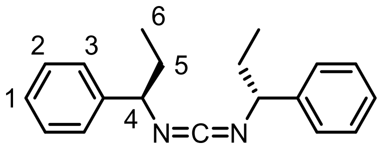

Enantiomeric excess was determined by HPLC (Chiralpak IA), *n*-hexane/*i*-propanol =99:1, 25 °C, 210 nm, 1.0 mL/min, retention times: t_R (major) 5.31 min, t_R (minor) 5.61 min, ee = 99%.

^{1}H NMR (600 MHz, CD_3CN) δ 7.35 – 7.20 (m, 10H, $H_{1,2,3}$), 4.30 (t, J = 7.0 Hz, 2H, H_4), 1.77 – 1.66 (m, 4H, H_5), 0.83 (t, J = 7.3 Hz, 6H, H_6).

^{13}C NMR (151 MHz, CD_3CN) δ 143.8, 140.5, 129.3, 128.3, 127.4, 63.8, 32.4, 11.2.

HRMS (ESI^+) calcd. For $C_{19}H_{23}N_2$ $[M+H]^+$ = 279.1856, found 279.1858.

S2.3 Synthesis of α,ω-diazido-poly(ethylene glycol) PEG$_M$

(The subscript M denotes the number average molecular weight of the polymer)

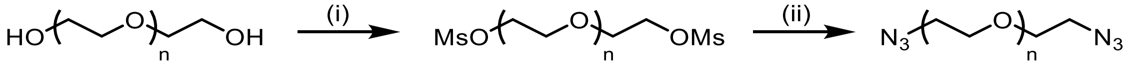

Scheme S3. Reagents and conditions: (i) MsCl, CH_2Cl_2, r.t., 2 days. ii) NaN_3, DMF, 50 °C, 24 h.

α,ω-dimesylate-poly(ethylene glycol)

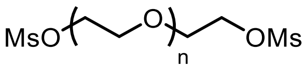

In a typical experiment, α,ω-dihydroxy-poly(ethylene glycol) (1.0 g) with number average molecular weight, *M*, was placed in a flask and triethylamine (10 equiv.) was added. The flask was cooled to 0°C and a solution of methanesulfonyl chloride (10 equiv.) dissolved in dichloromethane (8 mL) was added dropwise over the course of one hour. After complete addition, the solution was allowed to warm to room temperature and stirring was pursued overnight. The mixture was then concentrated under vacuum and the solid was reprecipitated twice in cold isopropanol (100 mL) from dichloromethane (10 mL) to afford α,ω-dimesylate-poly(ethylene glycol) as a white powder.

^{1}H NMR (600 MHz, $CDCl_3$) δ 4.38 (m, 4H), 3.78 – 3.50 (m, n × 4H), 3.08 (s, 6H).

$PEG_{3,000}$: yield: 58%, degree of functionalization: quant.

$PEG_{6,000}$: yield: 95%, degree of functionalization: quant.

α,ω-diazido-poly(ethylene glycol)

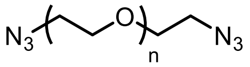

In a typical experiment, compound α,ω-dimesylate-poly(ethylene glycol) (500 mg) was dissolved in DMF (2 mL). Sodium azide (10 equiv.) was then added and the mixture was stirred for two days at room temperature. The solvent was partially evaporated under reduced pressure and the solution was diluted with dichloromethane (3 mL). The mixture was dropped in cold diethyl ether (50 mL) and the solid was collected by filtration to afford α,ω-diazido-poly(ethylene glycol) as a colourless solid.

^{1}H NMR (600 MHz, $CDCl_3$) δ 3.79 – 3.50 (m, n × 4H), 3.39 (m, 4H).

$PEG_{3,000}$: yield: 31%, degree of functionalisation: quant.

$PEG_{6,000}$: yield: 90%, degree of functionalisation: quant.

S3. Preparation of gel-1

S3.1 Synthetic procedure for formation of gel-1

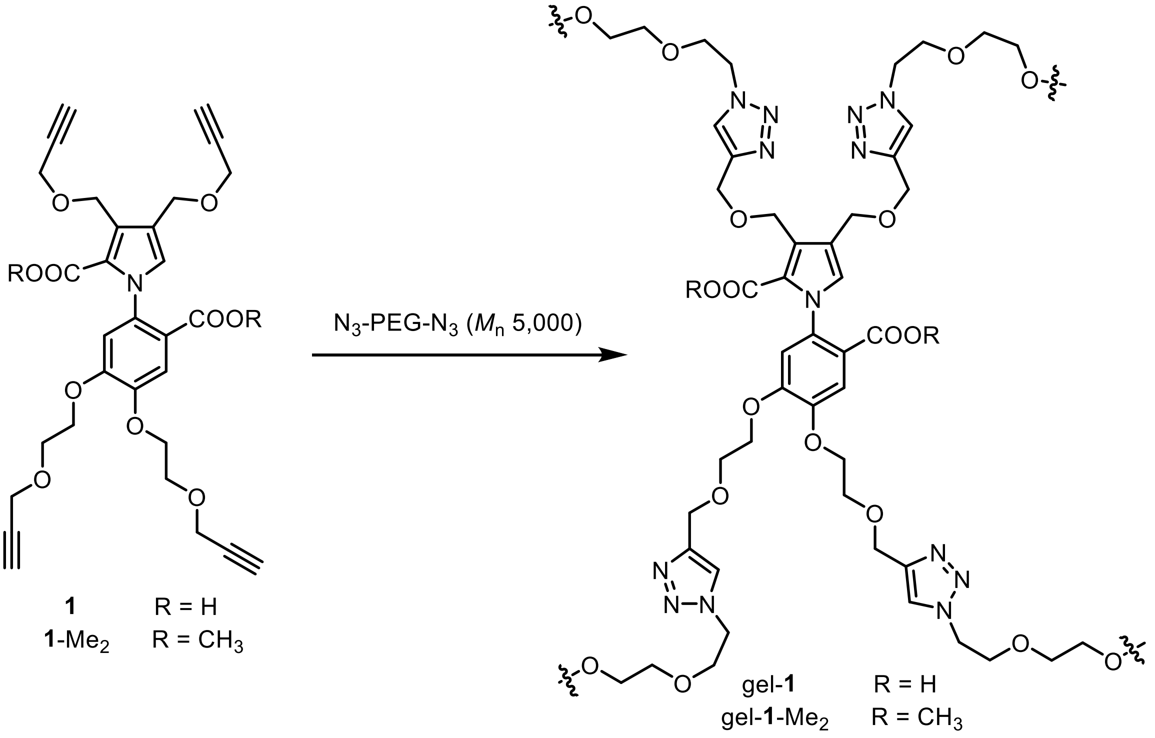

Scheme S4. Reagents and conditions: CuBr, PMDETA, CH_2Cl_2, r.t., 10 min.

To a solution of polyoxyethylene bis(azide) (average M_n 5,000) (12 mg, 2.4 μmol), CuBr (3.4 mg, 24 μmol), *N*,*N*,*N*',*N*'',*N*''-pentamethyldiethylenetriamine (5 μL, 24 μmol) in degassed CH_2Cl_2 (200 μL) was added a solution of **1** (0.7 mg, 1.2 μmol) in degassed CH_2Cl_2 (200 μL).* The gel was formed within 10 min and was washed extensively with CH_3CN, aqueous sodium ethylenediaminetetraacetate (Na_4-EDTA), and water. A colourless and transparent gel-**1** was obtained (7.6 mg dry gel, 60% yield).

Formation of gel-**1**-Me_2 was performed similarly to gel **1** but using **1**-Me_2. Polymerization of **1**-Me_2 (0.7 mg, 1.2 μmol) and polyoxyethylene bis(azide) (average M_n 5,000) (12 mg, 2.4 μmol) yielded gel-**1**-Me_2 (7.0 mg dry gel, 55% yield).

In order to form gels in a uniform shape suitable for the fuel contraction experiments, after the reagents were mixed (at the point marked * above), the mixture was quickly homogenized (<2 minutes) and transferred to a custom-made stainless steel mould of 20 x 20 x 1 mm (height x width x depth). After 30 min the gel was transferred from the mould to a closed bottle with 50 mL of CH_3CN, and agitated on a shaking plate (shaking frequency 160 turn per minute) for 30–45 min; the procedure was repeated,

washing the gel with different solvents: CH_3CN; then H_2O; then EDTA solution at pH = 9 (11.7 g/L adjusting the pH to 9.0 with ~2 M NaOH); then H_2O; then EDTA solution at pH = 9; then H_2O twice; then a mixture of H_2O/dioxane (5:8) twice. Total number of washings = 10.

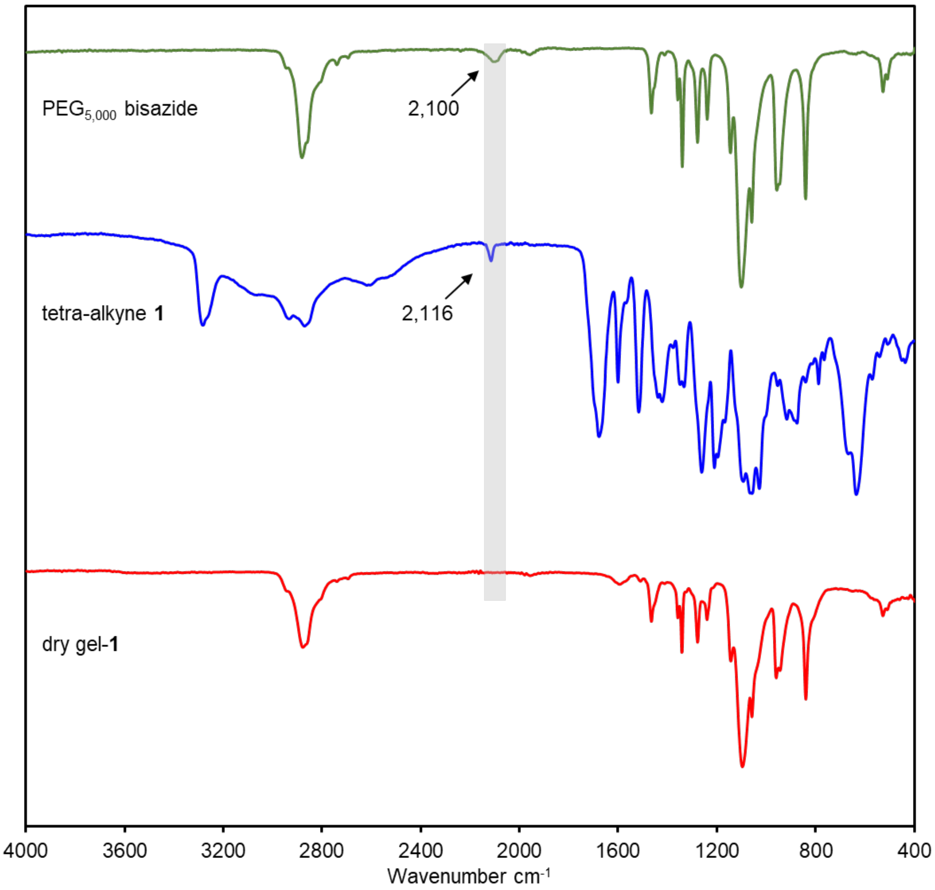

Spectrum S1. Stacked FT-IR spectra (ATR, neat) of $PEG_{5,000}$ bisazide, tetra-alkyne **1** and dry gel-**1**. The FT-IR spectrum of dry gel-**1** (red) shows no stretches at 2,100 and 2,116 cm^{-1}, indicating the successful click reaction.

S3.2 Optimisation of gelation conditions

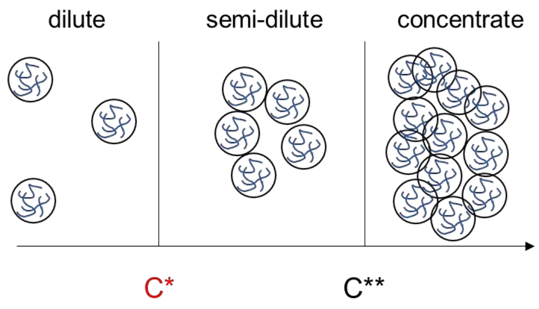

Figure S1. Schematic representation of the critical gelation concentration, C*.[S6]

The crosslinking of polyethylene glycol chains to form and contract a polymer gel is highly condition/concentration dependent. To achieve optimum gelation and actuation, the critical overlap concentration C* must be reached before crosslinking the gel (Figure S1).[S6] At concentrations below C*, crosslinking will be incomplete, and gelation will not occur or dangling chains will not sustain the winding process generated by the motor. If the concentration is too high, already existing physical crosslinks will decrease the capacity to create new entanglements by motor rotation. A range of conditions and concentrations were screened to approach the ideal C* conditions as shown in Table S1.

Table S1. Optimisation of conditions for the gel preparation.[S6]

Mass of motor **1** /mg	$PEG_{5,000}$ bisazide	CuBr	PMDETA	Solvent	Reaction temperature / °C	Reaction time	Gel formation observed?
0.35 mg	2 eq.	5 eq.	5 eq.	DMF (100 µL)	50	16 h	NO
0.35 mg	2 eq.	20 eq.	20 eq.	DMF (100 µL)	80	16 h	NO
0.35 mg	2 eq.	20 eq.	20 eq.	CH_2Cl_2 (80 µL)	20	16 h	NO
0.35 mg	2 eq.	20 eq.	20 eq.	CH_2Cl_2 (100 µL)	20	16 h	YES
1.30 mg	2 eq.	15 eq.	15 eq.	CH_2Cl_2 (200 µL)	20	1 min	YES
0.70 mg	2 eq.	20 eq.	20 eq.	CH_2Cl_2 (400 µL)	20	10 min	YES (gel-**1**)
0.70 mg	2 eq.	20 eq.	20 eq.	CH_2Cl_2 (410 µL)	20	16 h	NO

S3.3 Optimisation of solvent conditions for gel swelling

In order to best observe the effects of directional rotation of the motor on the gel network, it was important to ensure maximum swelling of the polymer gel. It was necessary to establish optimal solvent conditions to balance solubility of the reagents with gel swelling. The volume that the gel-**1** swells to varies significantly with solvent and can also be influenced by the solute. Consequently, compared to previously reported operation conditions for the rotary motor,[S5] buffer was removed (the high ionic strength[S7] proved incompatible), and the percentage of water was slightly increased in order to allow greater swelling of the gel, resulting in an optimised operating solvent of dioxane/H_2O (8:5, *v*/*v*). Gel samples were found to equilibrate in this solvent mixture within 1 h (indicated by a constant size of the gel) and then re-equilibrate for 4 h after the subsequent addition of a hydrolysis promoter.

S4. Evaluation of directional rotation

S4.1 Chemical fuelling of motor 1

[**1**] = 1 mM, [DMAP] = 1 mM, [DIC] = 10 mM, [MES buffer] = 100 mM (pH_{obs} 5.10 in D_2O) in dioxane-d_8/D_2O (8:5, *v*/*v*).

Motor **1** (4 µL of a 0.2 M stock solution, 1 mM) and DMAP (4 µL of a 0.2 M stock solution, 1 mM) were dissolved in 2-(*N*-morpholino)ethanesulfonic acid MES-buffered (100 mM, pH_{obs} 5.10) dioxane-d_8/D_2O (800 µL, 8:5, *v*/*v*). Diisopropyl carbodiimide (DIC) (10 eq., [DIC] = 10 mM) was added and the sample was monitored by ^{1}H NMR spectroscopy (Figure S2).

DIC was converted to diisopropylurea (DIU) waste, and transient formation of anhydride of motor **1** was observed.

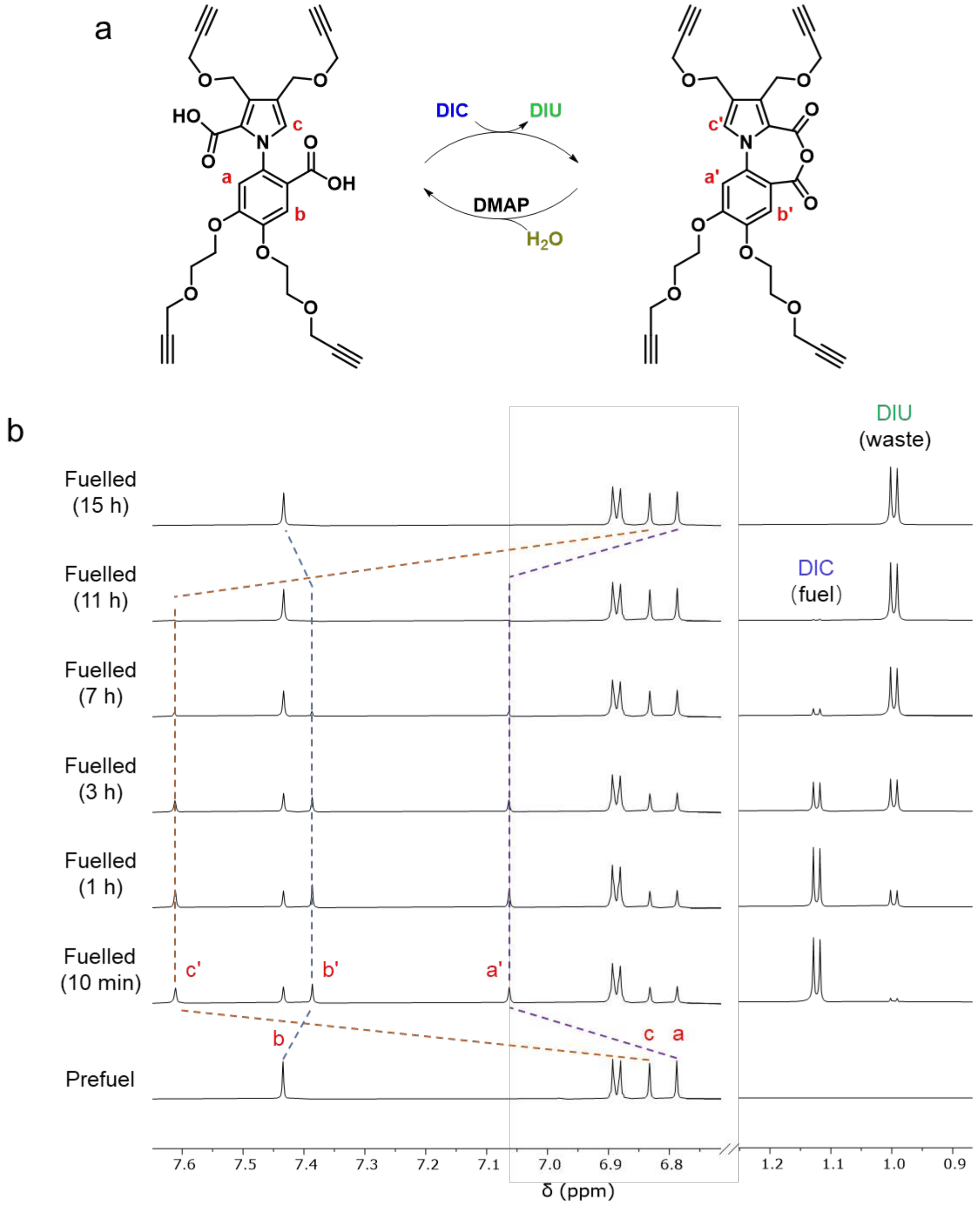

Figure S2. Fuelling of motor **1**. **a** Reaction cycle and **b** partial ^{1}H NMR spectra (dioxane-d_8/D_2O (8:5 *v*/*v*), 600 MHz, 298 K) showing transient formation of the anhydride of motor **1** upon treatment with diisopropylcarbodiimide. The region 6.7–7.7 ppm is scaled vertically 60× compared to region 0.8–1.3 ppm.

S4.2 Directional rotation of model motor 5 under unbuffered conditions

Racemic (±)-**5** (0.2 mg, 0.1 mM) and (*R*)-**4** (2.6 mg, 5 mM) were dissolved in unbuffered dioxane-d_8/D_2O (800 µL, 8:5, *v*/*v*). (*R*,*R*)-**2** (2.2 mg,10 mM) was added, and the sample was kept at room temperature for 4 days. The ratio of enantiomers of **5** was determined by chiral HPLC (ChiralPak-IF column, 25 °C, CH_2Cl_2:*i*-PrOH:CF_3CO_2H (95:5:0.1, *v*/*v*/*v*)).

S4.3 ^{1}H NMR analysis of the catalytic efficacy of gel-1

[Gel-**1**] = (in terms of motor units) estimated at 0.1 mM, [(*S*)-**4**] = 5 mM, [DIC] = 10 mM or [(*S*,*S*)-**2**] = 20 mM in unbuffered dioxane-d_8/D_2O (8:5, *v*/*v*).

To evaluate the ability of gel-**1** to catalyse the fuel-to-waste reaction, gel-**1** (estimated concentration of motor units = 0.1 mM) and (*S*)-**4** (2.5 mg, 5 mM) were placed in an NMR tube with dioxane-d_8/D_2O (800 µL, 8:5, *v*/*v*). DIC (1.0 mg, 10 mM) or [(*S*,*S*)-**2**] (4.4 mg, 20 mM) was added and the sample was monitored by ^{1}H NMR spectroscopy.

An identical experiment was performed without gel-**1** to establish a background rate of DIC hydration.

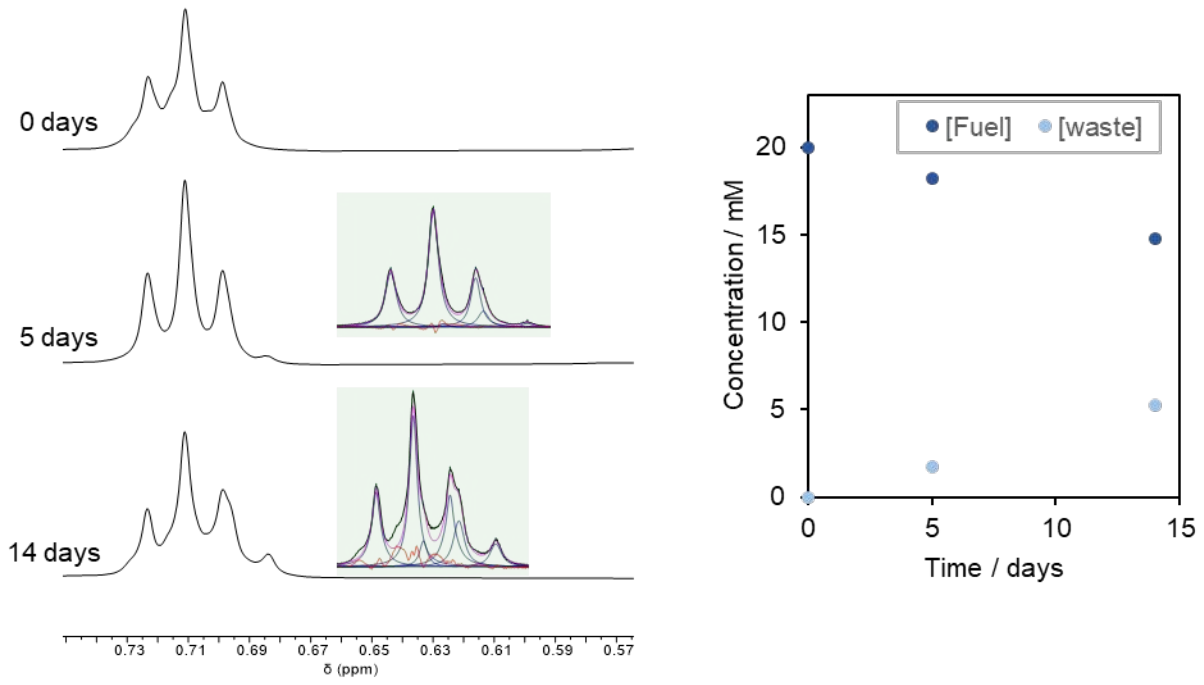

Figure S3. Plot of the consumption of [(*S*,*S*)-**2**] fuel for the gel-**1**-catalysed reaction, showing catalysis of the fuel-to-waste reaction by gel-**1**. Lorentzian/Gaussian peak fitting was used to deconvolute overlapping resonances and estimate integrals of fuel and waste signals.

S5. Gel contraction experiments

S5.1 Image recording

Still and video images were recorded using a Veho DX-3 USB camera. The gel was illuminated with a LIU525A 525 nm green light emitting diode array light source for easier visualisation. Still images were either independently recorded or taken as captures from continuous video recording.

S5.2 Image analysis

Digital images were analysed using ImageJ software (Figure S3), which was used to measure the size of the gel. The outline of the gel could be traced and the size measured. The 15 mm diameter of the quartz cell was used as a reference scale.

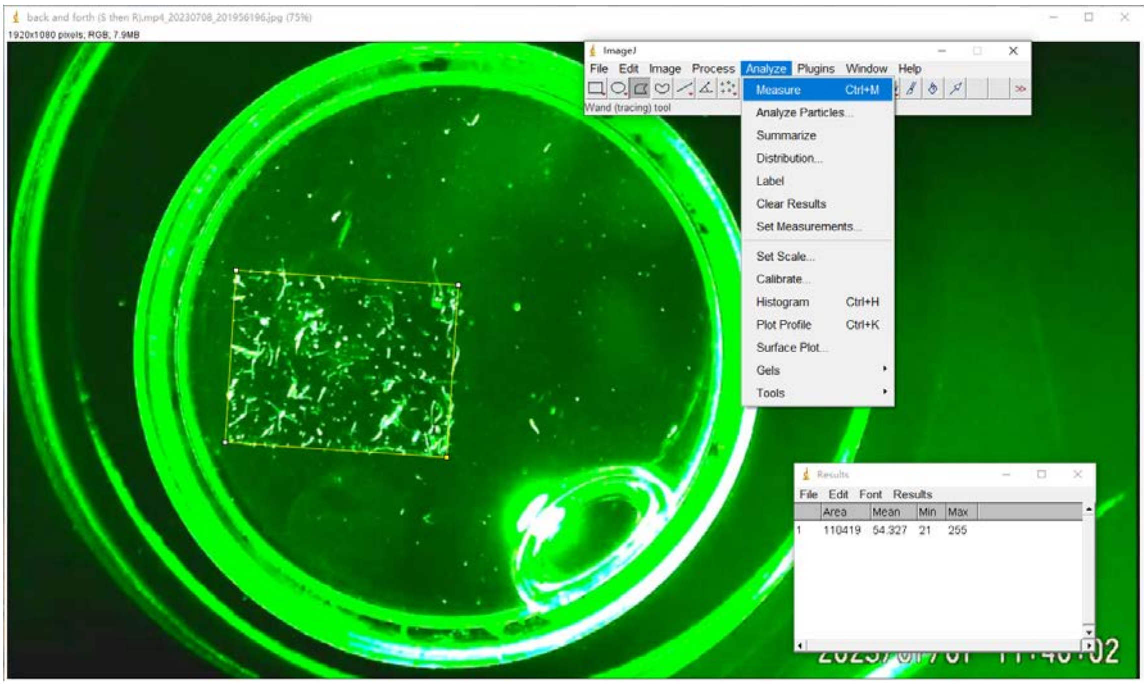

Figure S4. Measuring the area of the gel from the snapshots using ImageJ software.

S5.3 Contraction experiments with gel-1

(*S*)-**4** (4 µmol, 50 equiv.) was dissolved in dioxane/H_2O (800 µL, 8:5, *v*/*v*). To the solution was added a square sample of gel-**1** measuring approx. 10 × 10 × 1 mm (approximate [motor] = 0.08 µmol, 1 equiv.). The gel was allowed to equilibrate for 4 hours (see Section S3.3). Subsequently, (*S*,*S*)-**2** (8 µmol, 100 equiv.) was added and the gel size was monitored by video recording (see Section S5.1).

A similar experiment was performed by substituting (*R*)-**4** and (*R*,*R*)-**2** for (*S*)-**4** and (*S*,*S*)-**2**.

Another experiment was performed by substituting (*S*)-**4** and DIC for (*S*)-**4** and (*S*,*S*)-**2**.

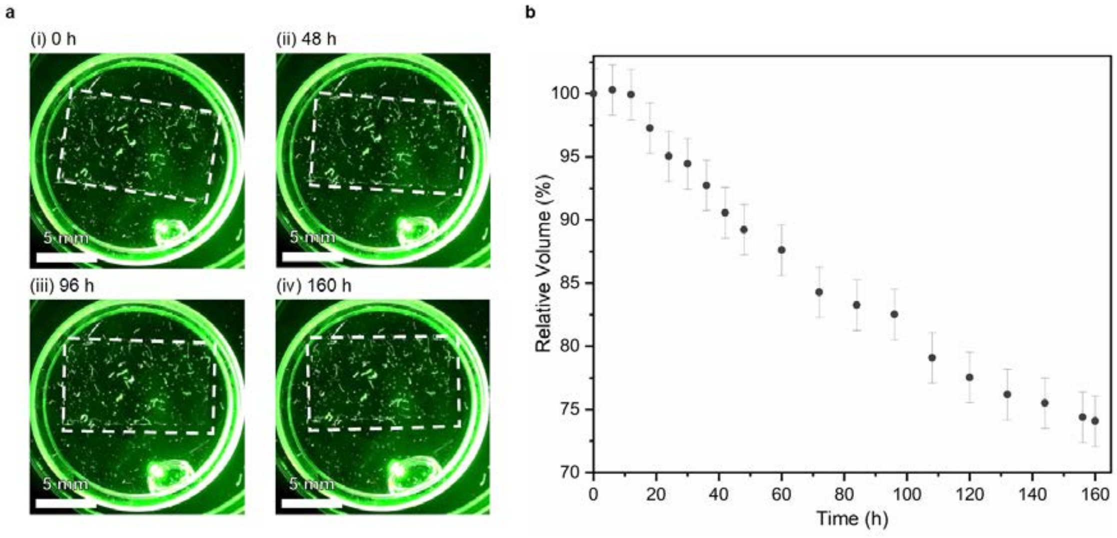

Figure S5. (a) Images of gel-**1** during chemically fuelled contraction with (*S*,*S*)-**2** and DIC at (i) 0 h, (ii) 48 h, (iii) 96 h and (iv) 160 h. The white dashed line shows the outline of the gel prior to fuelling (*t* = 0). (b) Contraction of gel-**1** under chemical fuelling. The change in %volume was plotted over time, with contraction continuing under fuelling for 7 days.

This experiment results in slightly less contraction (reaching a final contracted volume of 74% after 160 h (c.f. contraction with chiral fuel, contracted volume = 70% after 160 h)). This reduced extent of gel contraction is consistent with the reduction in directionality of rotation of the motors as a consequence of removing the fuelling gating. Quantitative comparisons between these experiments are difficult due to differences in swelling and osmotic pressure between the gels incorporating DIC/DIU vs fuel **2**/waste **3**.

S5.3.1 Gel contraction experiments with varied length of PEG_M

Formation of $gel_{PEG3,000}$ and $gel_{PEG6,000}$ were performed similarly to gel-**1** but using $PEG_{3,000}$-bisaizde and $PEG_{6,000}$-bisaizde respectively. Polymerisation of **1** (1.2 mg) and polyoxyethylene bis(azide) (average M_n 3,000) (12.8 mg) generated $gel_{PEG3,000}$. Polymerization of **1** (0.55 mg) and polyoxyethylene bis(azide) (average M_n 6,000) (11.7 mg) generated $gel_{PEG6,000}$.

The contraction experiments were performed as described in S5.3.

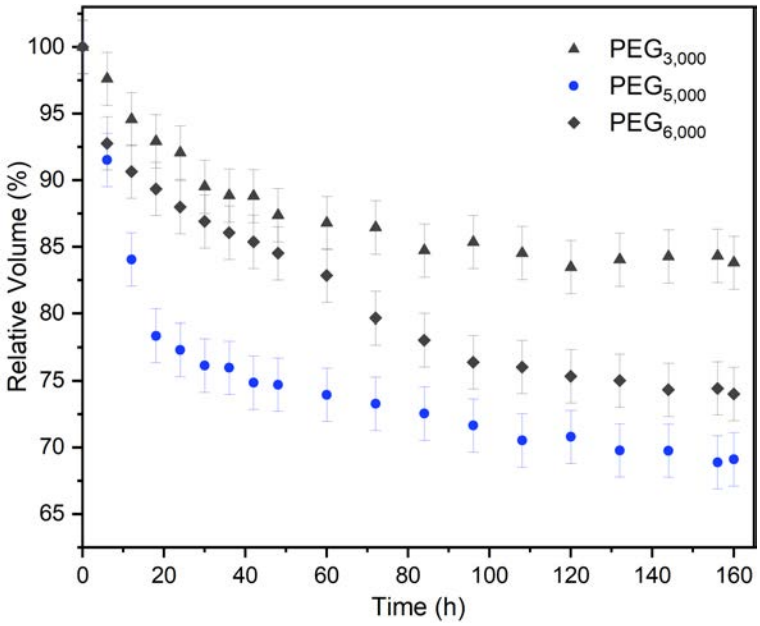

Figure S6. Fuelled contraction of **$gel_{PEG3,000}$**, **$gel_{PEG5,000}$** (**gel-1**), and **$gel_{PEG6,000}$**. Gels made with $PEG_{5,000}$ gave the largest contraction under fuelling, with both longer and shorter polymer strand gels contracting less. The differences in contraction between the different gels under fuelling reflect that the different lengths (and ratios of PEG:motor) will statistically produce different topologies, such as loops that will not enter into an ideal regular network and will not participate in the elastic modulus. Furthermore, the chemical nature of the motor (aromatic regions and H-bonding groups) will produce different levels of residual aggregation with the different PEG:motor ratios, leading to other heterogeneities and a variation of the proportion of defects depending on the M_n/concentration of the system.

S5.4 Control gel contraction experiments

S5.4.1 Treatment of gel-1 with only hydrolysis promoter (*S*)-4

A rectangular sample of gel-**1** was placed in dioxane/H_2O (800 µL, 8:5, *v*/*v*) and equilibrated for 4 hours. To this was added (*S*)-**4** (5 mM) and the gel size was monitored (Figure S4).

A slight swelling of the gel was observed. The motors in the gel are unable to rotate in the absence of fuel, so this swelling is being driven by diffusion of (*S*)-**4** into the polymer gel.[S8]

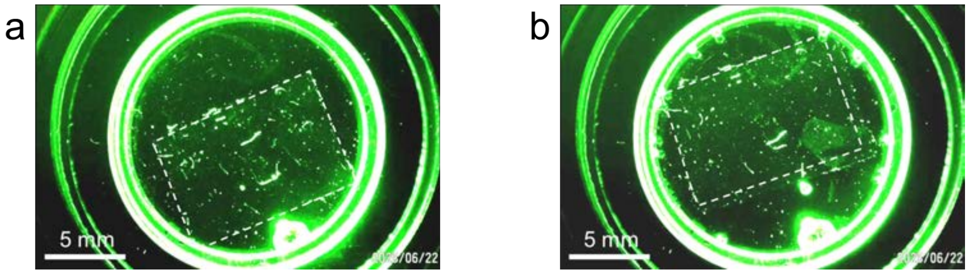

Figure S7. Snapshots of the gel-**1** in dioxane/H_2O (800 µL, 8:5, *v*/*v*) **a** before and **b** 4 h after treatment with (*S*)-**4** (5 mM) at room temperature. The initial gel size is indicated in all images by the white outline.

S5.4.2 Treatment of gel-1 with achiral DMAP and DIC

A rectangular sample of gel-**1** was placed in dioxane/H_2O (800 µL, 8:5, *v*/*v*) and equilibrated for 4 hours. To this was added DMAP (5 mM), DIC (10 mM) and the gel size was monitored (Figure S5).

The motors in the gel do not rotate directionally under achiral fuelling, despite efficient catalysis of the fuel-to-waste reaction by the gel-embedded motors. Instead, the addition of DMAP and DIC caused an initial modest expansion of the gel associated with diffusion-driven swelling and a likely change in protonation state of the motor since DMAP is basic.

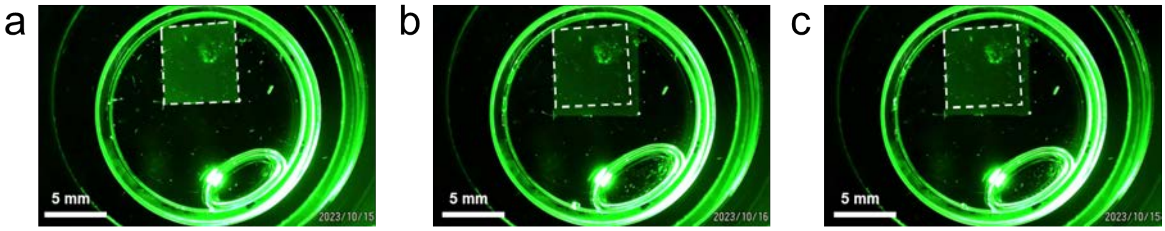

Figure S8. Snapshots of the gel-**1** in dioxane/H_2O (800 µL, 8:5, *v*/*v*) **a** before, **b** 4 h, **c** 24 h after treatment with DMAP (5 mM) and DIC (10 mM) at room temperature. The initial gel size is indicated in all images by the white outline.

S5.4.3 Operation of non-rotating gel-1-Me$_2$

(*S*)-**4** (5 mM) was dissolved in dioxane/H_2O (800 µL, 8:5, *v*/*v*). To the solution was added a rectangular sample of gel-**1**-Me$_2$. The gel was allowed to equilibrate for 4 hours (see Section S3.3). Subsequently (*S*,*S*)-**2** (10 mM, 100 equiv.) was added and the gel size was monitored.

No contraction was observed (Figure S6) despite the presence of both the chiral fuel and chiral anhydride catalyst, since the methylated motor cannot catalyse the fuel-to-waste reaction. Instead, a slight swelling was observed, driven by diffusion of (*S*,*S*)-**2** into the polymer network.[S8]

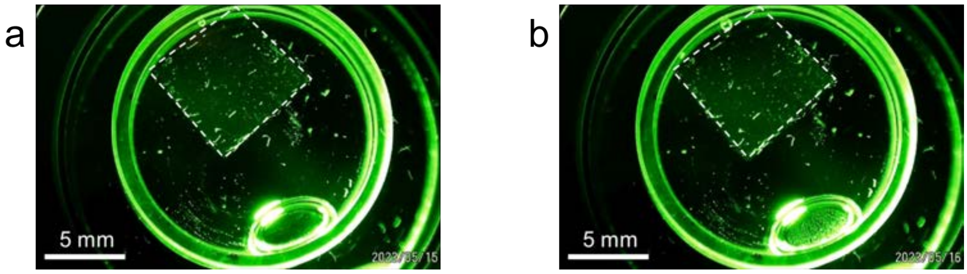

Figure S9. Snapshots of gel-**1**-Me$_2$ in the presence of (*S*)-**4** (5 mM) in dioxane/H_2O (800 µL, 8:5, *v*/*v*) **a** before and **b** 12 h after treatment with (*S*,*S*)-**2** (10 mM) at room temperature. The initial gel size is indicated in both images by the white outline.

S5.5 Gel expansion–contraction experiments

Initial fuelled gel contraction

Gel-**1** was contracted for 7 days with (*S*)-**4** and (*S*,*S*)-**2** as described in Section S5.3.

The contracted gel was washed twice with CH_3CN/H_2O (7:3, *v*/*v*) to remove all residual chiral carbodiimide fuel, urea waste and the chiral hydrolysis promoter. The resultant gel was equilibrated in dioxane/H_2O (8:5, *v*/*v*).

S5.5.1 Expansion–contraction – treatment with reagents of opposite chirality

To the pre-contracted gel in dioxane/H_2O (800 µL, 8:5, *v*/*v*) were added reagents of the opposite chirality to those employed for the initial contraction: (*R*,*R*)-**2** (10 mM) and (*R*)-**4** (5 mM). The gel size was monitored by video recording. Expansion–contraction behaviour was observed (manuscript, Figure 4).

S5.5.2 Control – treatment with reagents of the same chirality

To the pre-contracted gel in dioxane/H_2O (800 µL, 8:5, *v*/*v*) were added reagents of the same chirality to those employed for the first batch of fuelling: (*S*,*S*)-**2** (10 mM) and (*S*)-**4** (5 mM).

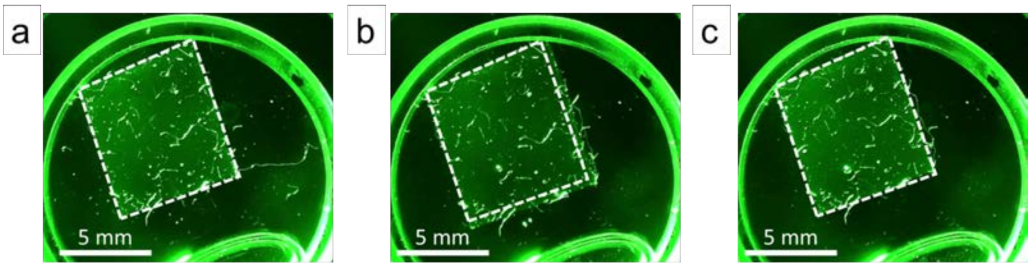

Figure S10. Snapshots of the pre-contracted (with (*S*,*S*)-**2** and (*S*)-**4**) gel-**1** in dioxane/H_2O (800 µL, 8:5, *v*/*v*) **a** before and **b** 3 h and **c** 40 h after treatment with (*S*,*S*)-**2** (10 mM) and (*S*)-**4** (5 mM) at room temperature. The initial gel size is indicated in all images by the white outline.

The modest expansion (~10%, Fig. S7b) is substantially less than when reagents of the opposite chirality to the first batch of fuel are used (manuscript, Figure 4) and is consistent with expansion driven by diffusion of the reagents into the polymer network (as observed in Section S4). The slight subsequent contraction is likely a result of additional motor units becoming accessible to the fuel after reswelling.

S5.5.3 Control – treatment with achiral reagents

To the pre-contracted gel in dioxane/H_2O (800 µL, 8:5, *v*/*v*) were added achiral reagents: DIC (10 mM) and DMAP (5 mM). The gel size was monitored by video recording (Figure S11). The observed expansion is consistent with fuelling enabling the acid groups on the rotor and stator to pass each other (by transiently forming the anhydride), resulting in the release of elastic energy stored in the twisted polymer strands of the gel by unravelling.

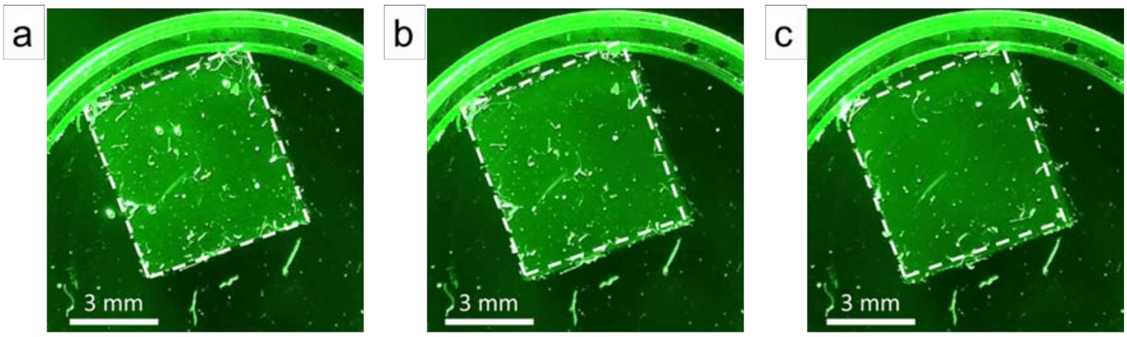

Figure S11. Snapshots of the pre-contracted (with (*S*,*S*)-**2** and (*S*)-**4**) gel-**1** in dioxane/H_2O (800 µL, 8:5, *v*/*v*) **a** before and **b** 72 h and **c** 144 h after treatment with DMAP (5 mM) and DIC (10 mM) at room temperature. The initial gel size is indicated in all images by the white outline.

S5.6 Thermal response of unfuelled and fuel-contracted gel-1

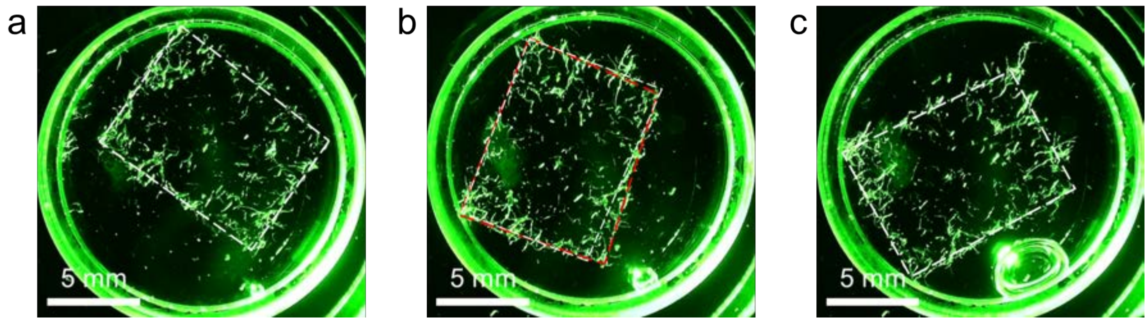

Figure S12. Snapshots of unfuelled gel-**1** a) before, b) heated to 70 ˚C, c) cooled down to r.t. The initial and after heating gel size are indicated in the images by the white and red outline respectively.

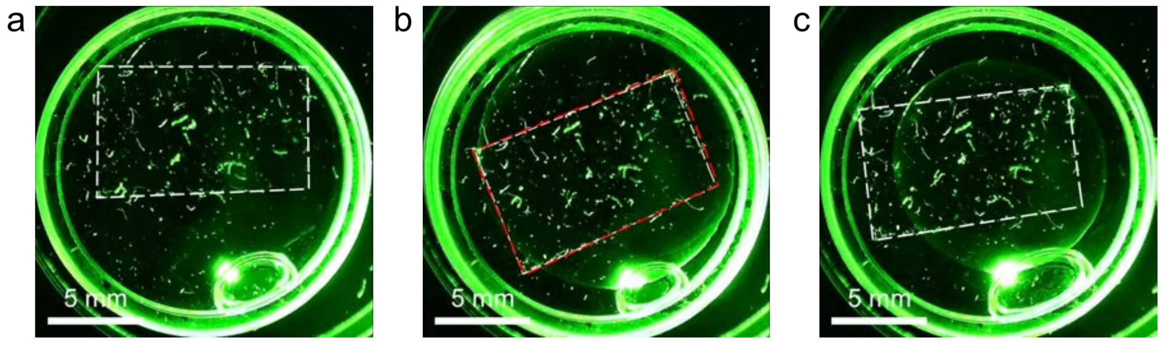

Figure S13. Snapshots of fuel-contracted **gel-1** a) before, b) heated to 70 ˚C, c) cooled down to r.t. The initial and after heating gel size are indicated in the images by the white and red outline respectively.

Upon heating, both the unfuelled and the fuel-contracted gels expand slightly (likely due to greater solvent incorporation resulting in increased swelling), and return to the original pre-heated size upon cooling back to room temperature. At 70 °C, the unfuelled gel expands to 107% of its original size, while the contracted gel expands to 104%. The lesser expansion of the contracted gel is consistent with the lower flexibility of the twisted polymer strands. We note that the twisted polymer strands in the contracted gel cannot unravel even at elevated temperatures, because of the very high activation energy barrier for the carboxylic acid groups on the rotor and stator of each motor to pass each other.

S6. Rheology and AFM experiments

Rheology measurements were performed with a TA instruments hybrid rheometer HR 3 equipped with a standard Peltier parallel plate steel with temperature control (20 mm diameter) set at 25 °C, and with a solvent trap to create a thermally stable vapor barrier, virtually eliminating any solvent loss during the experiment. Oscillation experiments were performed with a strain of 0.5% and frequency from 0.1 to 10 Hz. In the plots, all the points that showed a torque different from the displacement (i.e. Lissajous plot different from a straight line) were removed. Data shown in Figure 3a of the manuscript.

Atomic force microscopy (AFM) images were obtained by scanning the samples using a Nanoscope 8 (Bruker) operated in Peak-Force tapping mode. Peak-Force AFM is based on Peak force tapping technology, during which the probe is oscillating in a similar fashion as it is in tapping mode, but far below the resonance frequency. Each time the tip and the sample are brought together, a force curve is captured. These forces can be controlled at levels much lower than contact mode, and even lower than tapping mode, allowing operation on even the most delicate soft samples, as is the case here. Ultra-sharp silicon tip on nitride lever was used (Bruker, Scanasyst with spring constant of 0.4 N/m and tip radius of about 5 nm). During AFM imaging, the force was reduced in order to avoid dragging of molecules by the tip. Here the applied peak force is about 50 pN. All analyses of the images were conducted in integrated software. Data shown in Figure 3b and 4c of the manuscript and Figure S14.

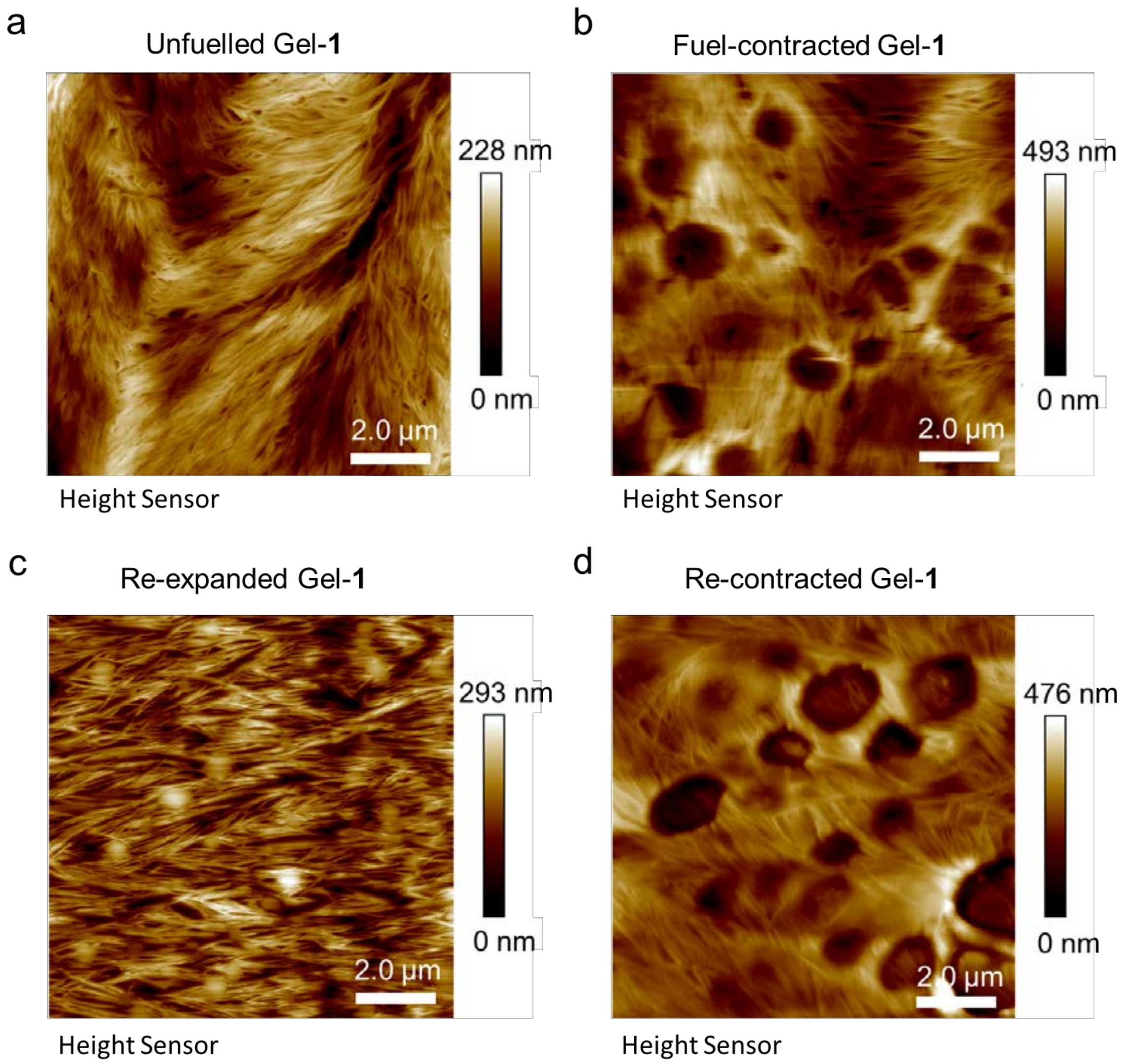

Figure S14. AFM images of gel-**1** a) unfuelled, b) fuel-contracted with (*S*,*S*)-**2** and (*S*)-**4** after 7 days, c) re-expansion with (*R*,*R*)-**2** and (*R*)-**4** after 5 hours, d) re-contraction with (*R*,*R*)-**2** and (*R*)-**4** after 7 days. The kinks and pores created as a consequence of the entanglements caused by fuelling with the (*S*)-fuelling system initially disappear when re-expanding the gel to its maximum size (i.e. at the point where most of the motor-induced twists in the chains have been unwound) by fuelling with the (*R*)-fuelling system. But continued rotation of the motors in this direction with the (*R*)-fuelling system then winds the polymer chains about each other again generating microscopic kinks and pores in the gel again.

S7. Tensile testing experiments

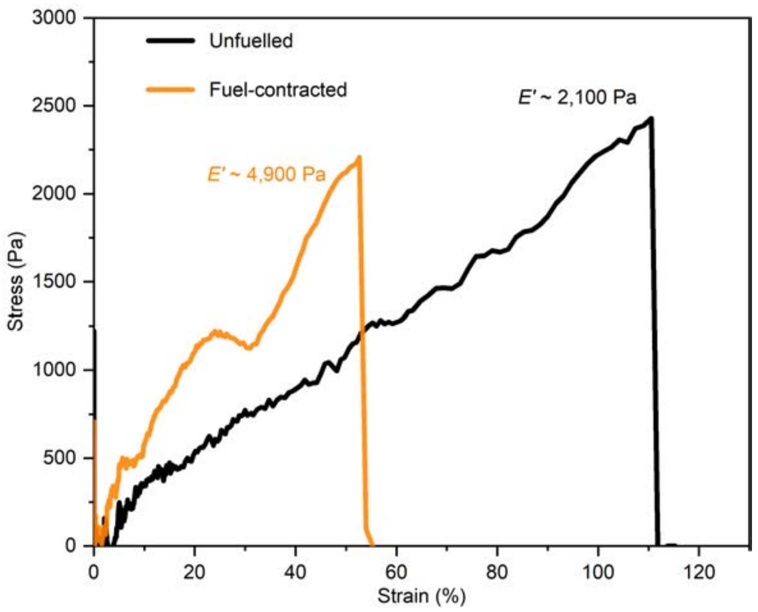

Figure S15. Tensile test of gel-**1**, unfuelled (black) and fuel-contracted (orange) with (*S*,*S*)-**2** and (*S*)-**4** after 7 days.

We used a Discovery Hybrid Rheometer 3 from TA Instruments, equipped with the torsion rectangular shape, to perform tensile tests. During the test, samples were fixed after perfect alignment of the sample holder, with the rotation blocked to allow only tensile stresses. We measured the sample width and fixed it to the sample holder. The mould used during fabrication ensured that the thickness (1 mm) was the same for all samples. The length of the sample was determined automatically by the rheometer using calibrations carried out before each test. Sample lengths varied from 5 to 12 mm. During the test, a continuous displacement of 10 µm/s was imposed and the normal force was acquired. The elastic modulus of the material was estimated using standard mechanics equations. The elongation and stress until crack were also determined.

The tensile tests show that the Young modulus of the fuel-contracted gel (4.9 kPa) is higher than that of the unfuelled gel (2.1 kPa), in agreement with the formation of new entanglements. We note that despite the increased stiffness of the gel upon fuel-driven contraction, the stress at break is still limited by defects in the gels (reaching 2.4 kPa for ~110% elongation in the unfuelled gel, and 2.25 kPa for ~55% elongation in the fuel-contracted gel).

S8. Non-ideal gels and energy storage

S8.1 Characterisation of the non-ideal gel

For approaching an ideal gel from chemical design, one should in principle crosslink a single component star polymer with equivalent lengths of polymer arms, and a homogeneous chemical nature.[S9] Here, we have a totally different topological design, with a central motor tetraalkyne (motor **1**), which is cross-linked with PEG bisazide in a bicomponent system. Consequently, the elastic network cannot be ideal because the connections will statistically produce different topologies, such as loops that will not enter into an ideal regular network and will not participate to the elastic modulus. Furthermore, the chemical nature of the motor (having aromatic ring and H-bonding groups) will likely produce some residual aggregation leading to other heterogeneities (indeed, as evidenced by the appearance of pores in the AFM images upon contraction, Figure 4c, re-expanded gel). To demonstrate the regular nature of a gel network, one should measure for different molecular weights of the polymer that the swelling ratio *Q* exhibits a power law correlation.[S10] We experimentally measured the equilibrium swelling ratio *Q* for the unfuelled gel made at c* for $PEG_{5,000}$, $PEG_{3,000}$ and $PEG_{10,000}$ (e.g. for $PEG_{5,000}$: $Q = V_{eq}/V_{dry} = 1 \times 10^{-2}$ cm^3/0.042 × 10^{-2} cm^3 = 23.8, where V_{dry} is the volume when dry and V_{eq} is the swelling volume at which the osmotic pressure and elastic contributions to the free energy are balanced). We did not find a power law correlation with other chain lengths, as expected given the nature of the chemical topology discussed above.

S8.2 Quantification of motor rotation in gel and elastic energy storage

Although the gels are not ideal as discussed above (Section S8.1), we can still make an approximation following the Panyukov model,[S11] which states that the elastic modulus of the gel $G(\phi)$ in a swollen state (either extended or contracted) is proportional to the polymer volume fraction and the chain number density, $\frac{\phi}{Nb^3}$, times the elastic free energy per chain. This is true in a good solvent regime and for $\frac{1}{Q} < \phi_0 < \phi^{**}$ (ϕ_0 is the polymer volume fraction at the concentration of gel preparation, and ϕ^{**} is the crossover concentration)

$$G(\phi) \cong kT \frac{\phi}{Nb^3}\left(\frac{\lambda R_0}{R_{ref}}\right)^2 \cong kT \frac{\phi}{Nb^3}\left(\frac{\phi_0}{\phi}\right)^{2/3}\left(\frac{\phi}{\phi_0}\right)^{(2\upsilon-1)/(3\upsilon-1)} \qquad \text{equation } \mathbf{S1}$$

Where υ = 0.588 in a good solvent[S11] $\lambda=(V/V_0)^{1/3}=(\phi_0/\phi)^{1/3}$ is the linear deformation, $R^2=(\lambda R_0)^2$ is the mean-square end-to-end distance of network strands in the final state (*e.g.* at equilibrium or in a swollen/de-swollen state), and R^2_{ref} is the mean-square fluctuation of the end-to-end distance of the network strand that in many cases is equal to the mean-square end-to-end distance of a free chain with the same number of monomers as the strand in the same solution (with here $N \approx 120$ PEG units). Before chemical fuelling, the concentration of polymer is the concentration of preparation, which is equal to the crossover concentration for the optimal system: $\phi = \phi_0 = \phi^*$ with linear deformation $\lambda = 1$. Using the experimental value of the modulus $G' = 300$ Pa, one obtains an initial number density of effective polymer strands equal to $(\phi/Nb^3)_{\text{initial}} \cong 300/kT \cong 0.73\times10^{23}$ strands m^{-3}, representing the number of strands that effectively participate in the elasticity of the gel. Note that other chains such as trapped entanglements, loops, dangling ends, and other connectivity defects are elastically ineffective strands that do not contribute to G. When contracted $\phi_0/\phi = V/V_0$ = (0.213 cm^3/0.314 cm^3) = 0.68 = λ^3 and G' = 1,400 Pa, one can extract a final number density $(\phi/Nb^3)_{\text{final}} \cong 3.4\times10^{23}$ strands m^{-3}, a value 4.7 times larger than the initial one indicating that N has decreased due to the formation of new entanglements.

This is a direct experimental observation that the motors rotation indeed twists polymer chains at the nanoscale leading to the formation of new entanglements which play the role of additional cross-links that increase the elastic modulus. The 4.7× increase in the number of polymer strands during the rotation/contraction process can be related to an energy, which is proportional to the number of new strands times the elastic energy per chain. It quantifies that this type of motorised system can convert chemical energy into elastic energy.

The calculated value for the increase in strand entanglements (4.7×) from the contraction data is in broad agreement with the number of rotations expected from the motor based on the kinetic gating and fuel use of the motor. With a selectivity (gating) of 1.1 for anhydride formation and 2.1 for anhydride hydrolysis, the motor is expected to perform an average of 0.20 net forward rotations per fuel-to-waste reaction catalysed by the motor (net forward rotations = forward cycles – backward cycles = $\left(\frac{1.1}{1.1+1}\times\frac{2.1}{2.1+1}\right)-\left(\frac{1}{1.1+1}\times\frac{1}{2.1+1}\right)$). Consequently, for the ~25 equivalents of fuel-to-waste reactions catalysed by the motor in a typical contraction experiment on gel-**1**, each motor-molecule would be expected to directionally rotate ~5.0 times on average. The

motor rotation is in good agreement with the increase in entanglement in the gel, especially given the assumptions in both calculations. The higher value for motor rotation vs the observed increase in entanglements may reflect a decrease in directionality as the motor works against the opposing elastic force of the gel, as well as the possibility of inactive strands/motors being trapped in heterogeneities in the gel structure.

We can tentatively relate these values to a transduction energy between catalysis and mechanical work. In each piece of gel used in these experiments, the following energy was produced: $G_{\text{cont}} \times V_{\text{cont}} - G_{\text{expand}} \times V_{\text{expand}} = 1400 \times 0.213\ 10^{-6} - 300 \times 0.314\ 10^{-6} = 0.000204$ J. There is 1 mg of motor in each piece of gel, that is 1.76 10^{-6} mol, thus giving a mechanical energy delivered by the motor equal to 0.116 kJ mol^{-1}, which is similar to the predicted maximum energy stored by a model motor in solution (Extended Data Fig. 1a) of 0.19 kJ mol^{-1} (according to: $\Delta G = RT\left(S_{\text{Eq}}^{H} - S_{\text{SS}}^{H}\right)$, where $S^{H} = -[(+)\text{-}\mathbf{1}]ln[(+)\text{-}\mathbf{1}] - [(-)\text{-}\mathbf{1}]ln[(-)\text{-}\mathbf{1}]$.[S12]

S9. NMR spectra

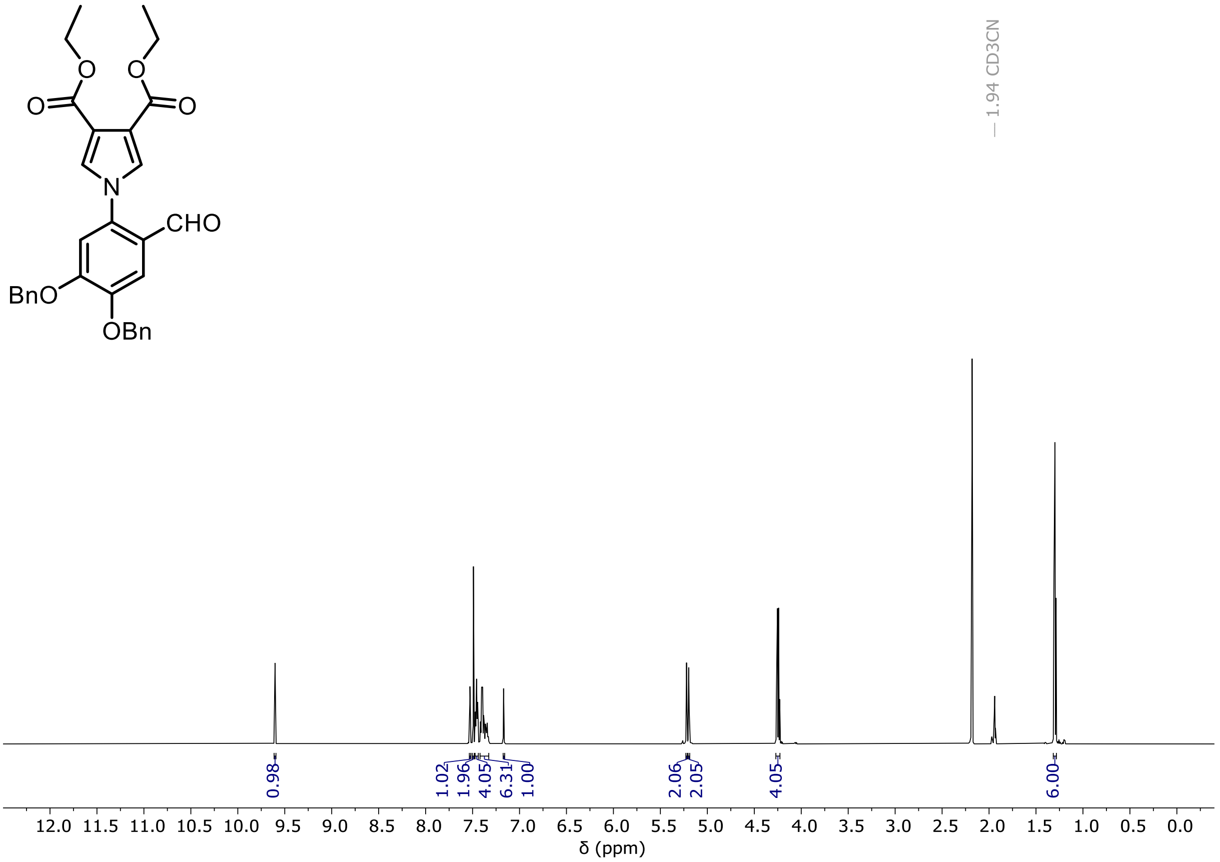

Spectrum S2: ^{1}H NMR spectrum (600 MHz, CD_3CN) of **S4**.

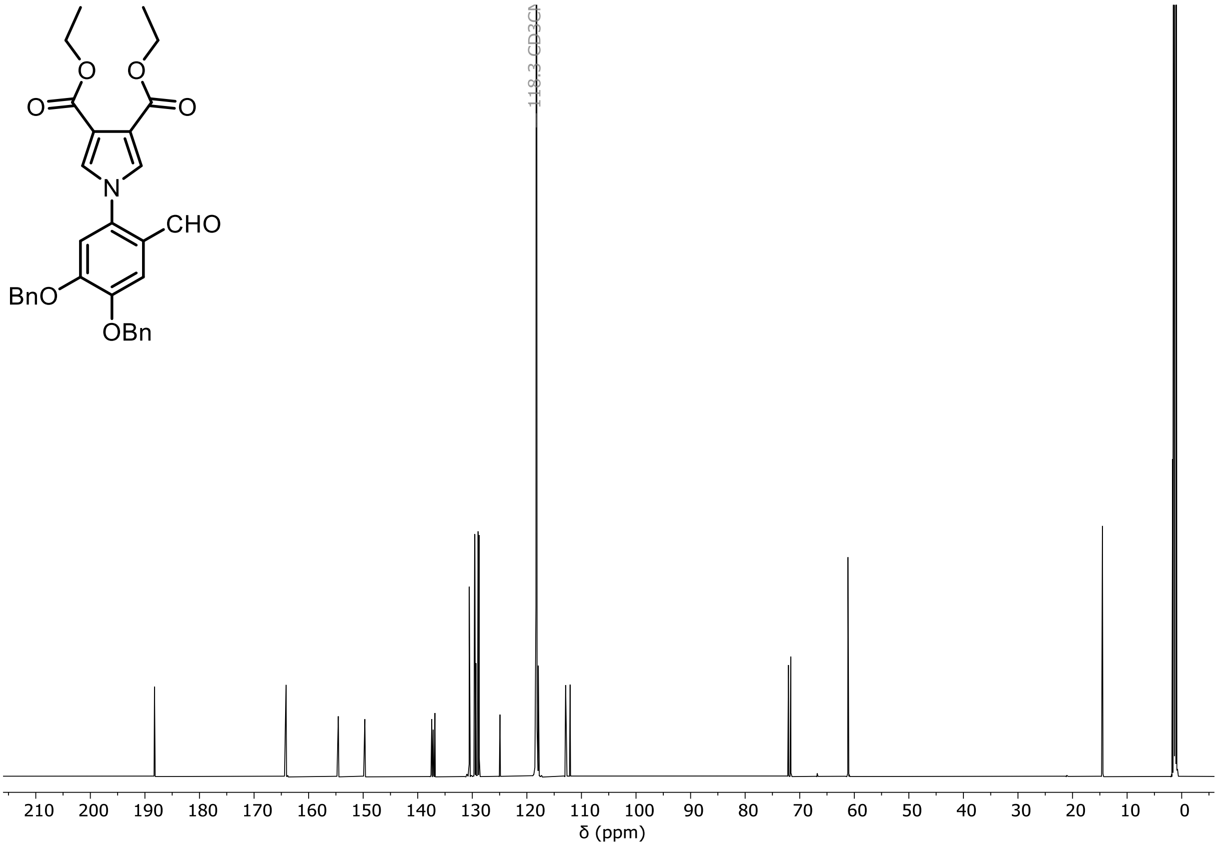

Spectrum S3: ^{13}C NMR spectrum (151 MHz, CD_3CN) of **S4**.

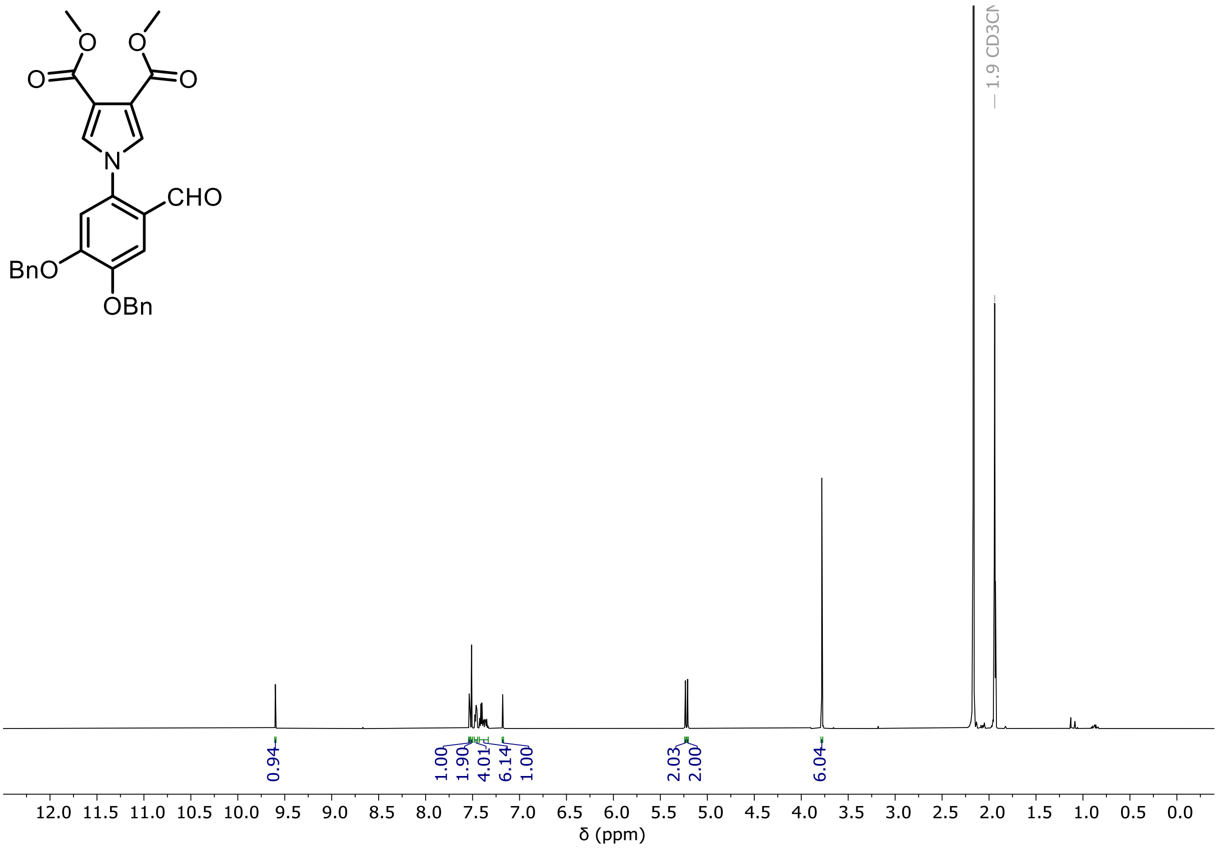

Spectrum S4: ^{1}H NMR spectrum (600 MHz, CD_3CN) of **S5**.

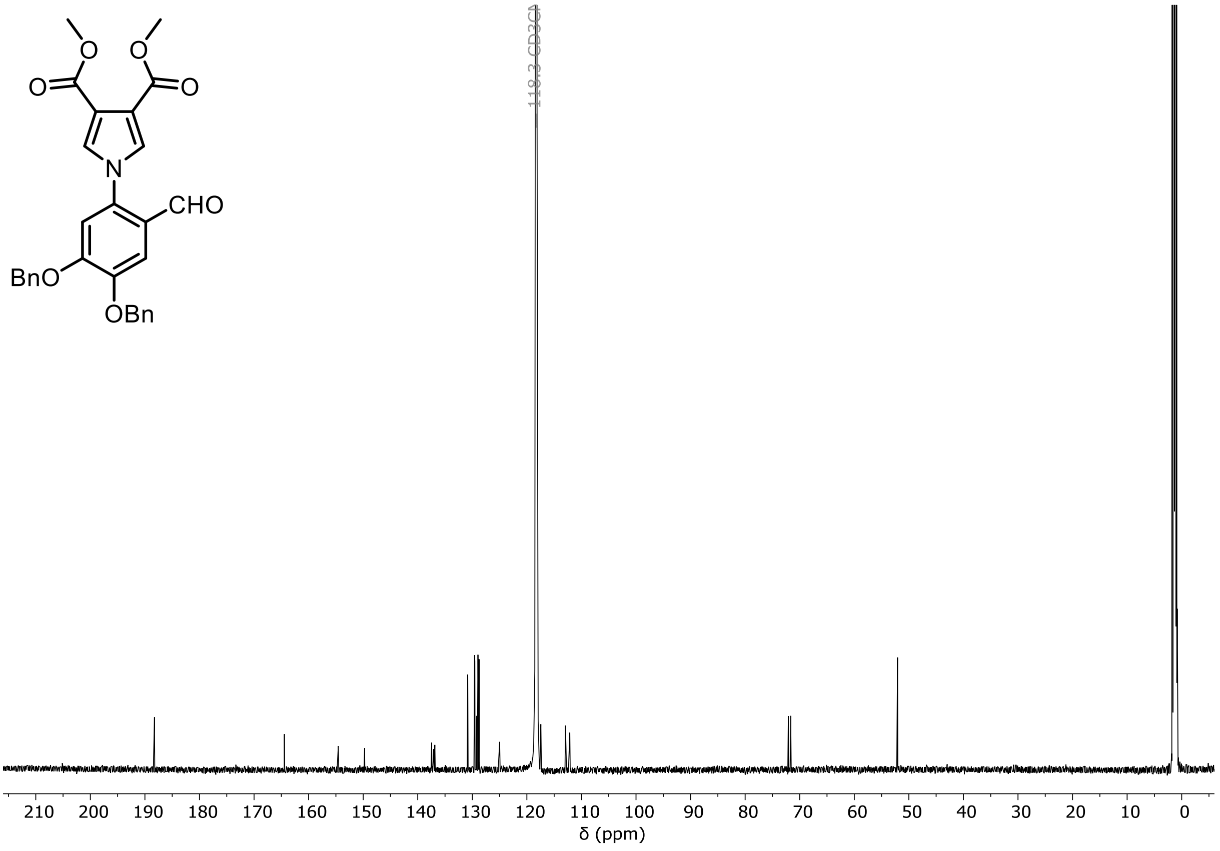

Spectrum S5: ^{13}C NMR spectrum (151 MHz, CD_3CN) of **S5**.

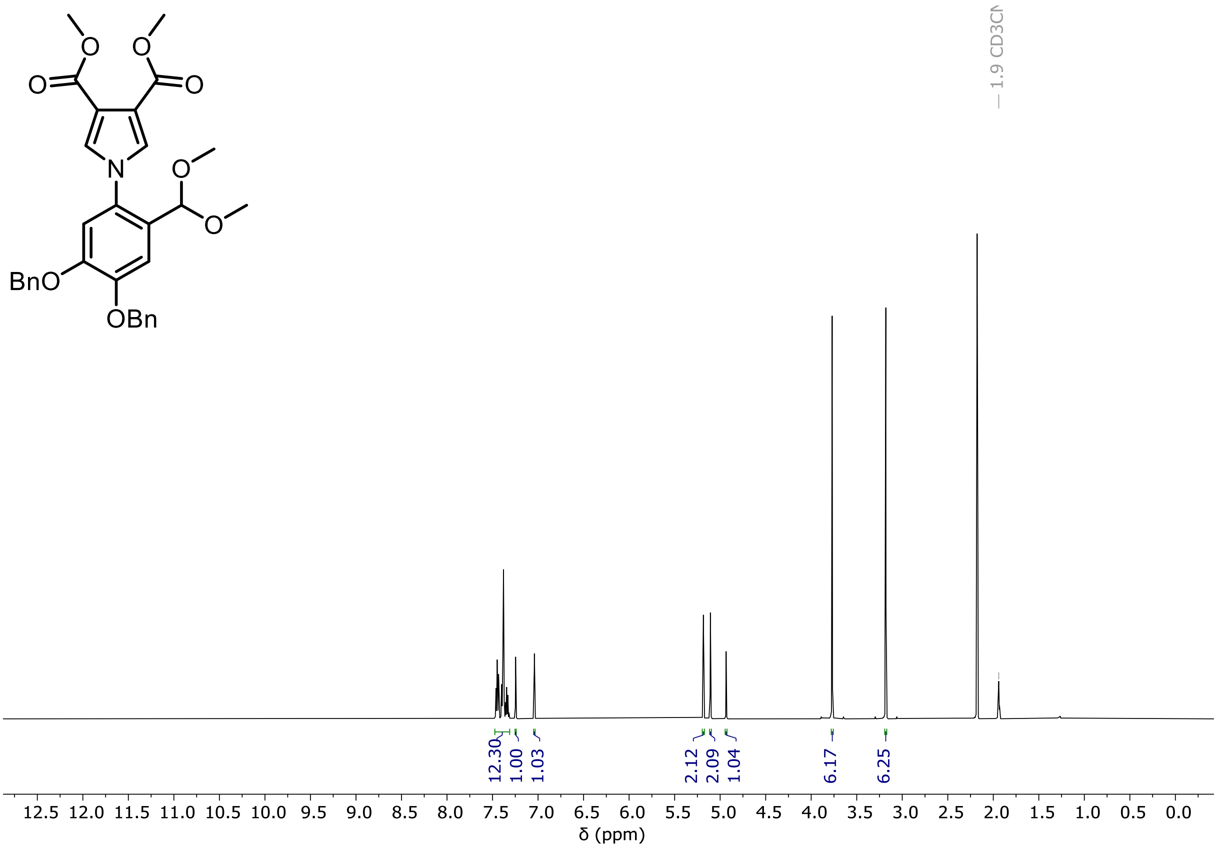

Spectrum S6: ^{1}H NMR spectrum (600 MHz, CD_3CN) of **S6**.

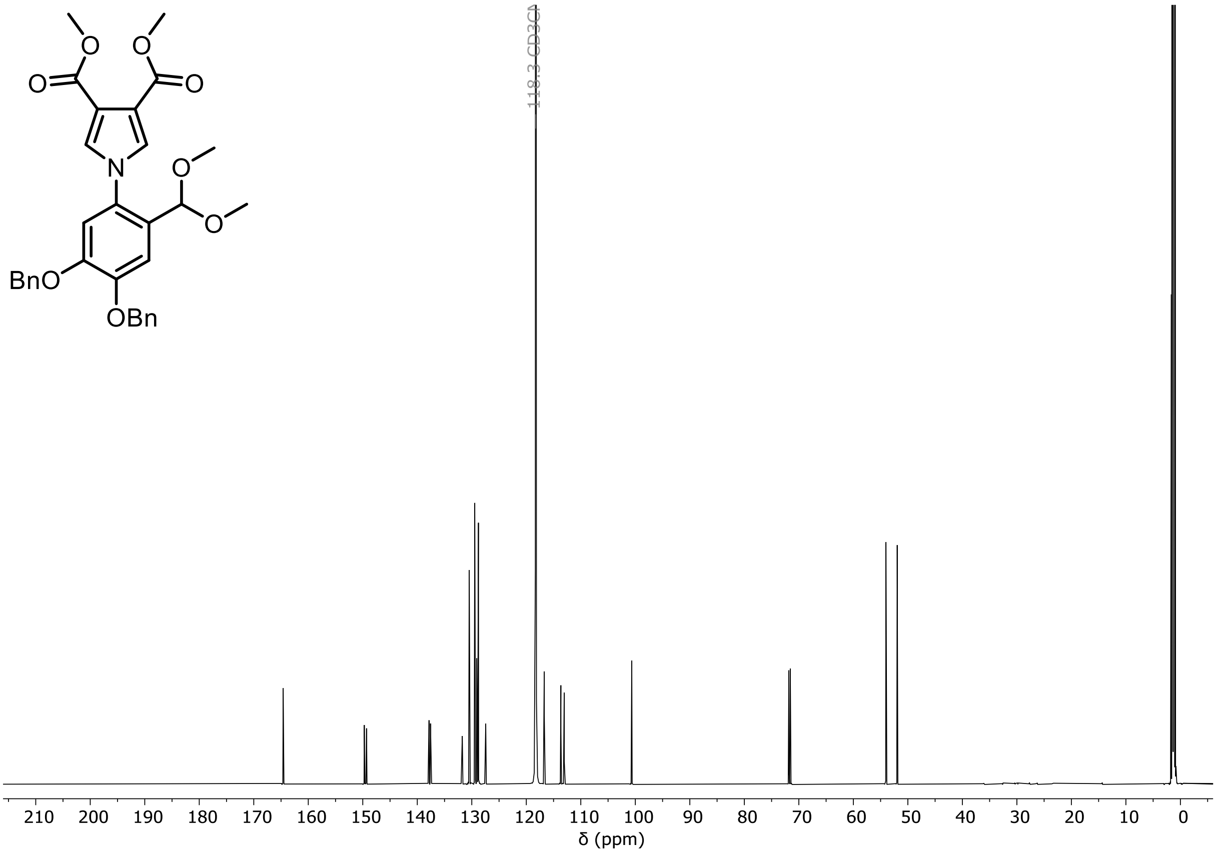

Spectrum S7: ^{13}C NMR spectrum (151 MHz, CD_3CN) of **S6**.

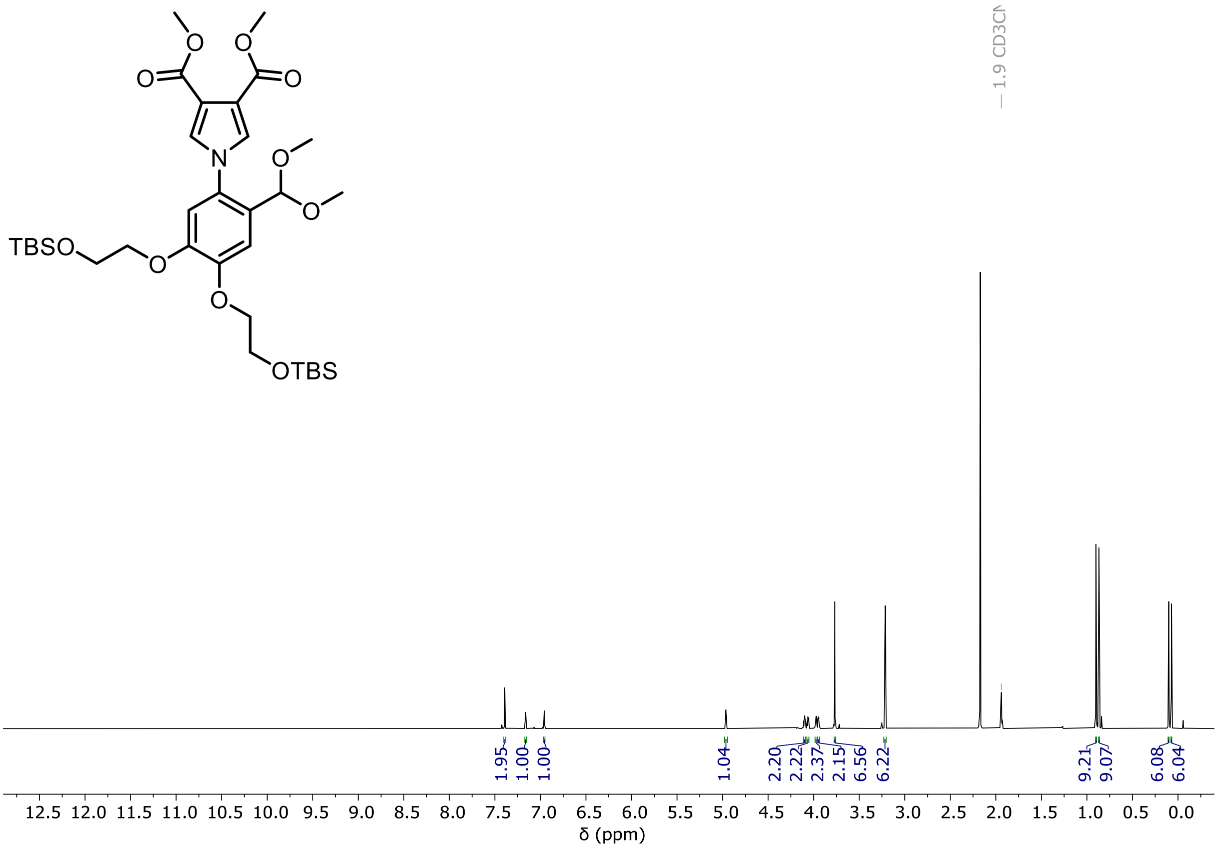

Spectrum S8: ^{1}H NMR spectrum (600 MHz, CD_3CN) of **S8**.

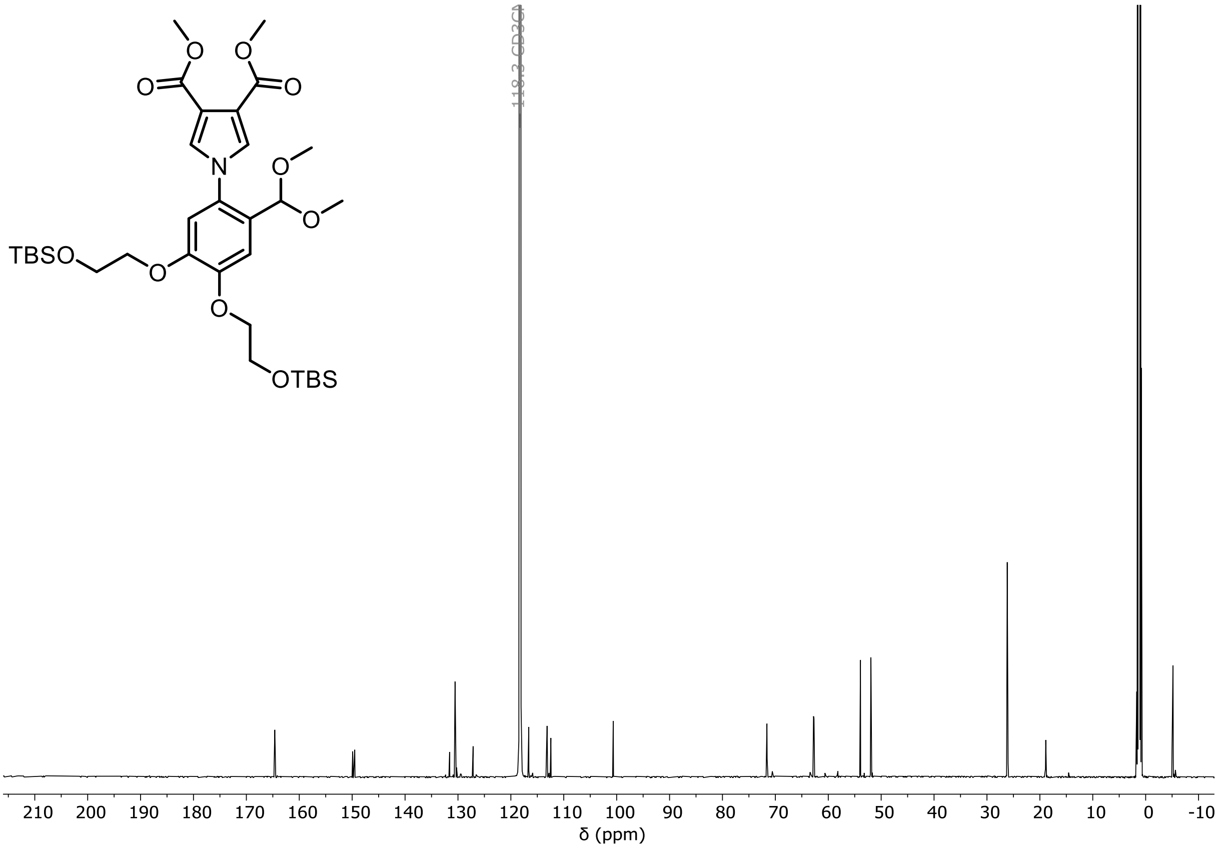

Spectrum S9: ^{13}C NMR spectrum (151 MHz, CD_3CN) of **S8**.

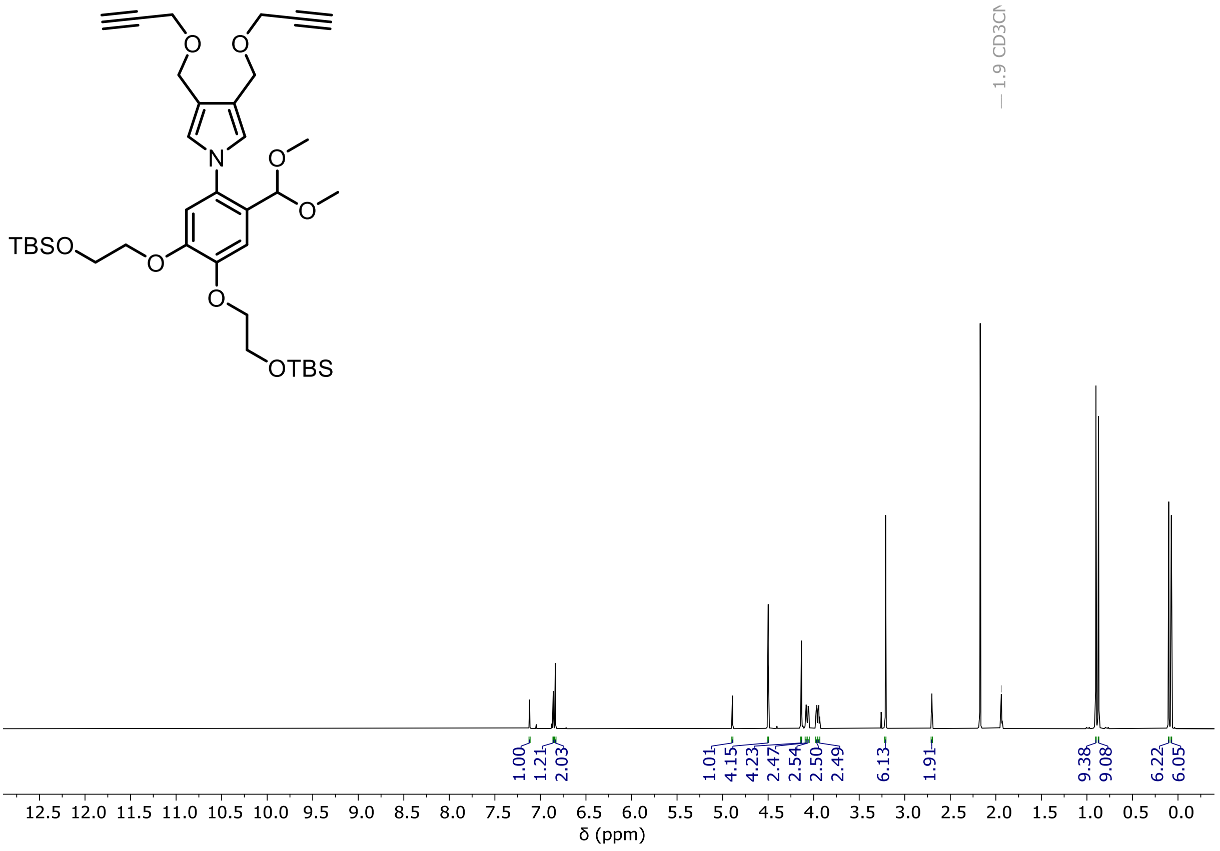

Spectrum S10: ^{1}H NMR spectrum (600 MHz, CD_3CN) of **S10**.

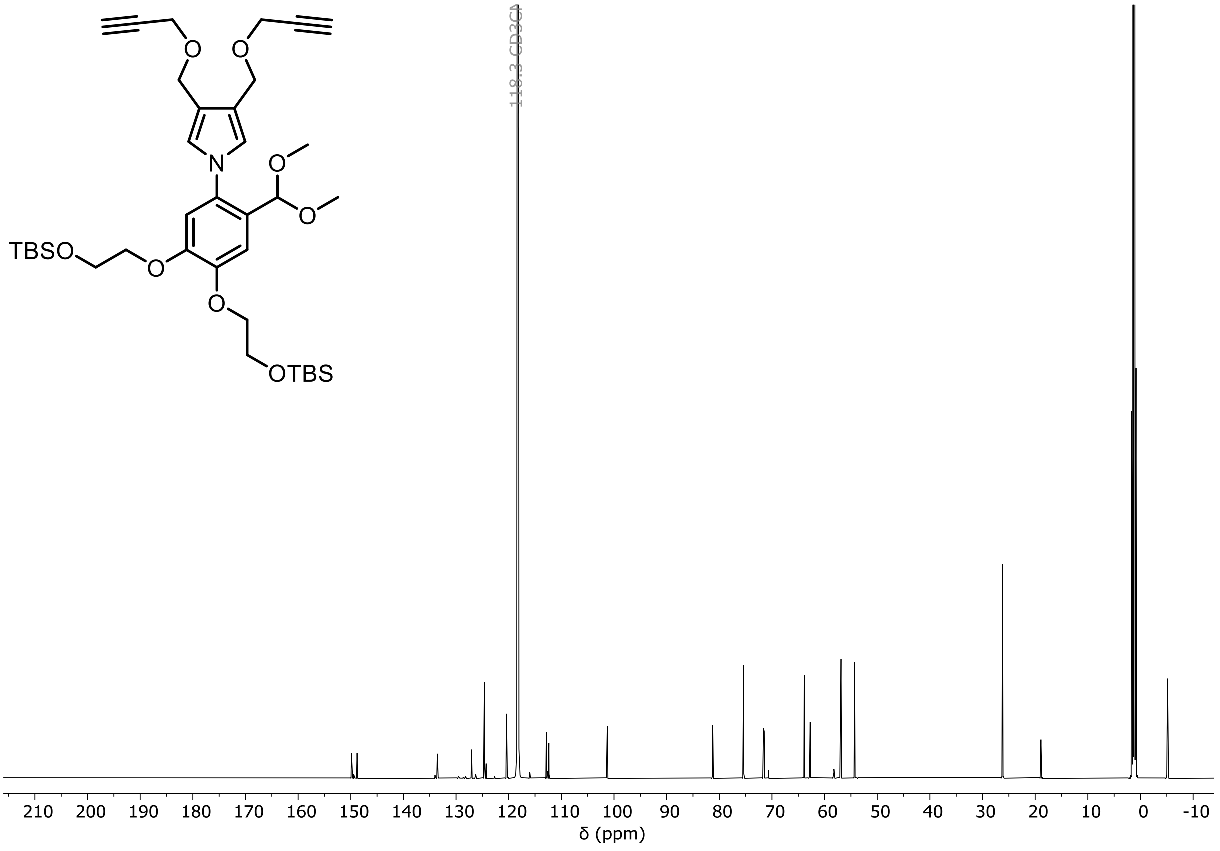

Spectrum S11: ^{13}C NMR spectrum (151 MHz, CD_3CN) of **S10**.

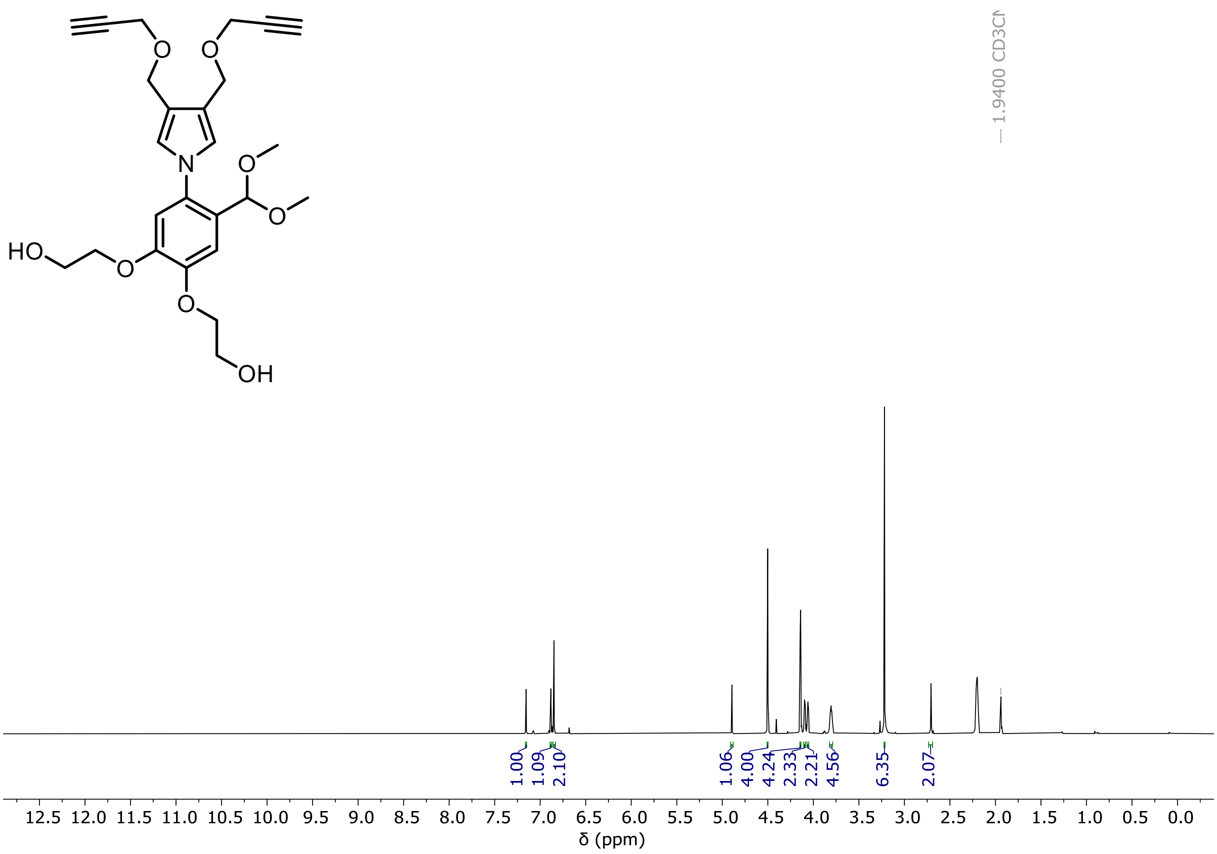

Spectrum S12: ^{1}H NMR spectrum (600 MHz, CD_3CN) of **S11**.

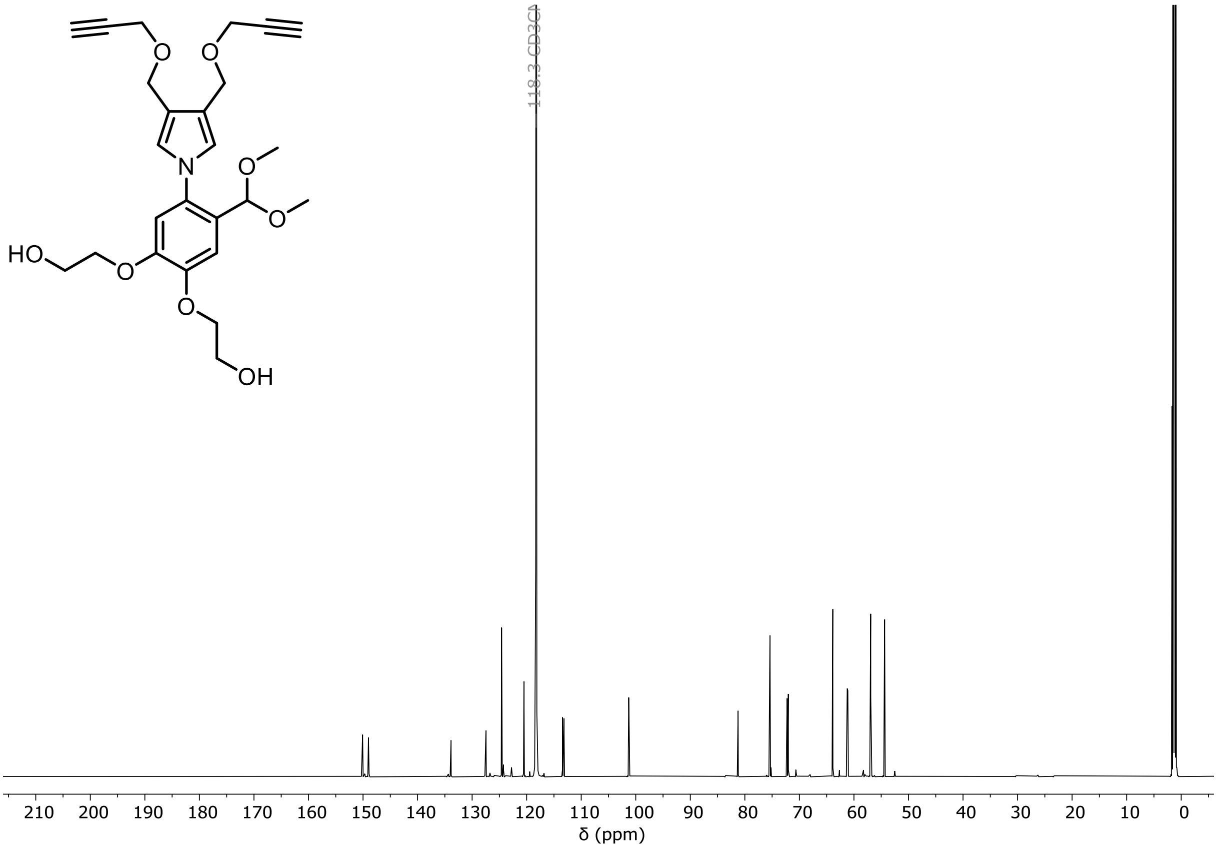

Spectrum S13: ^{13}C NMR spectrum (151 MHz, CD_3CN) of **S11**.

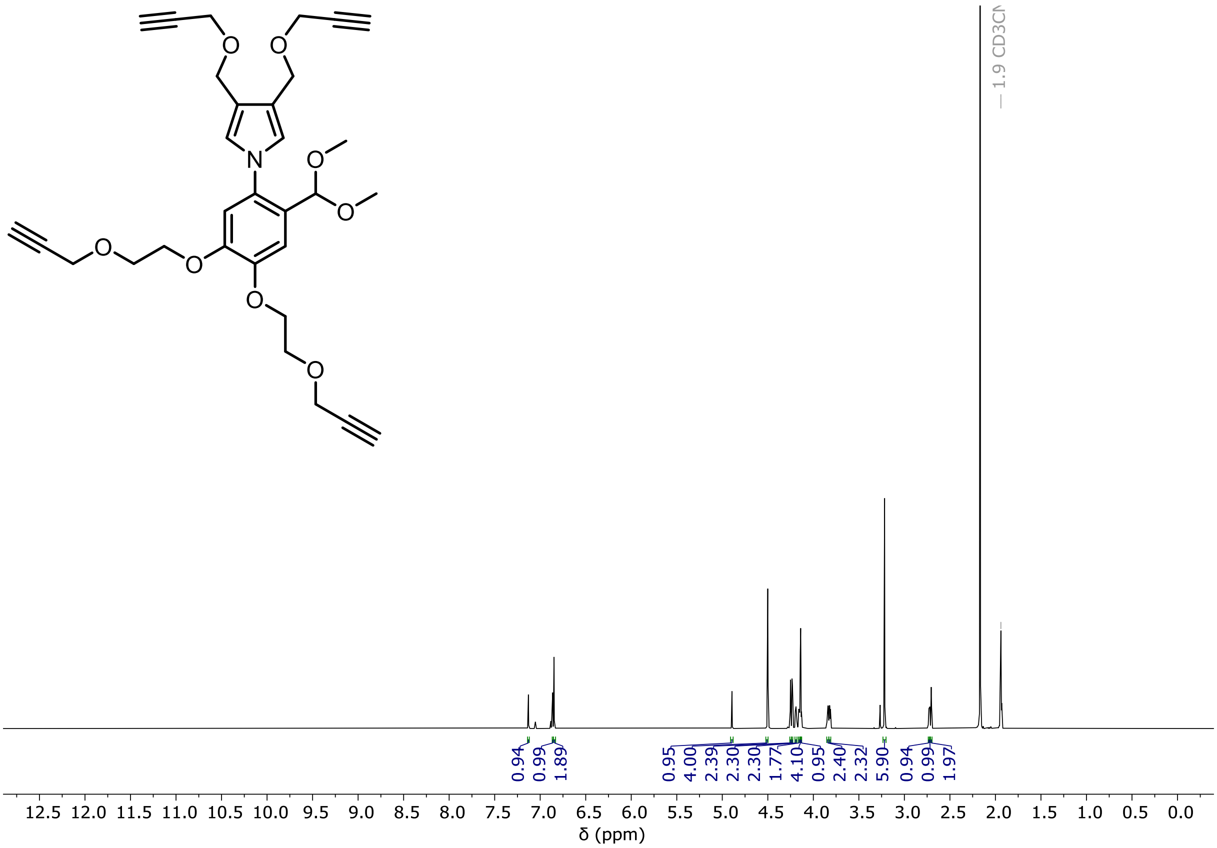

Spectrum S14: ^{1}H NMR spectrum (600 MHz, CD_3CN) of **S12**.

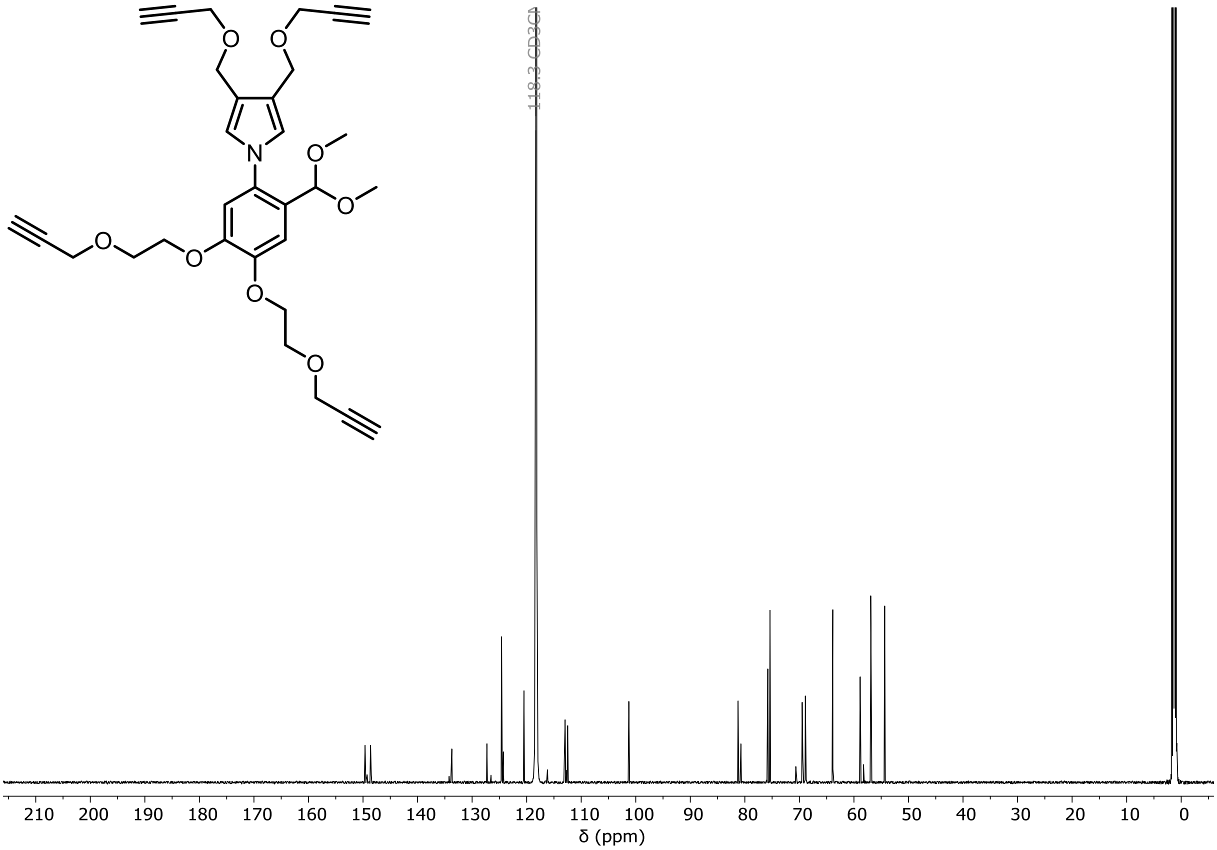

Spectrum S15: ^{13}C NMR spectrum (151 MHz, CD_3CN) of **S12**.

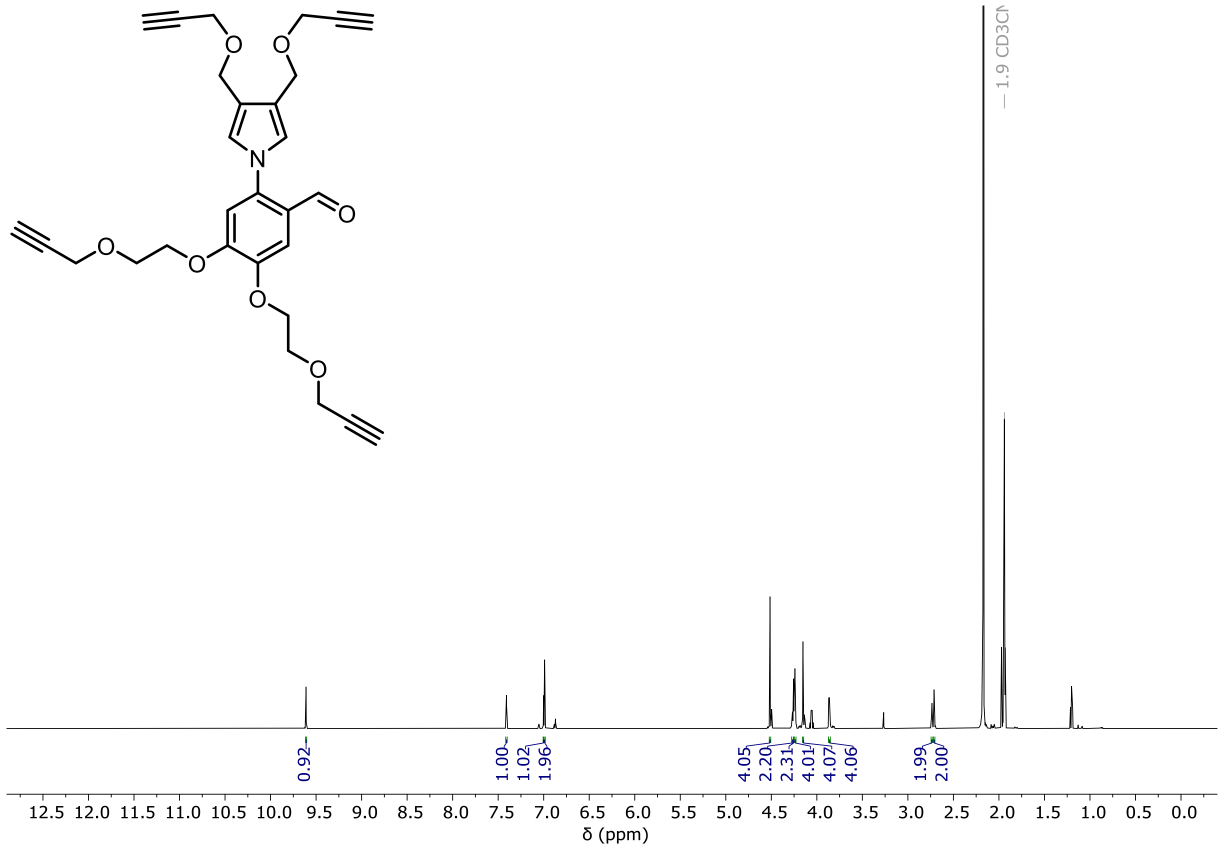

Spectrum S16: ^{1}H NMR spectrum (600 MHz, CD_3CN) of **S13**.

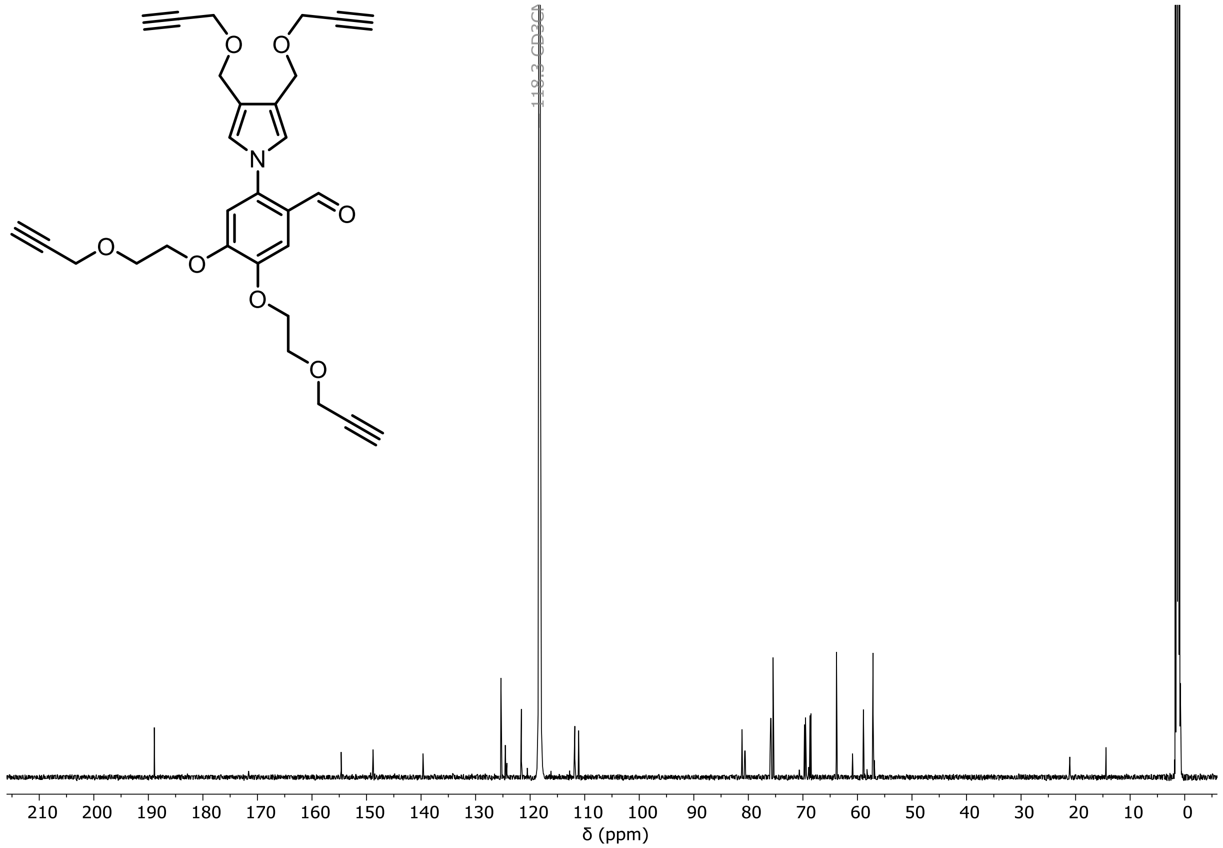

Spectrum S17: ^{13}C NMR spectrum (151 MHz, CD_3CN) of **S13**.

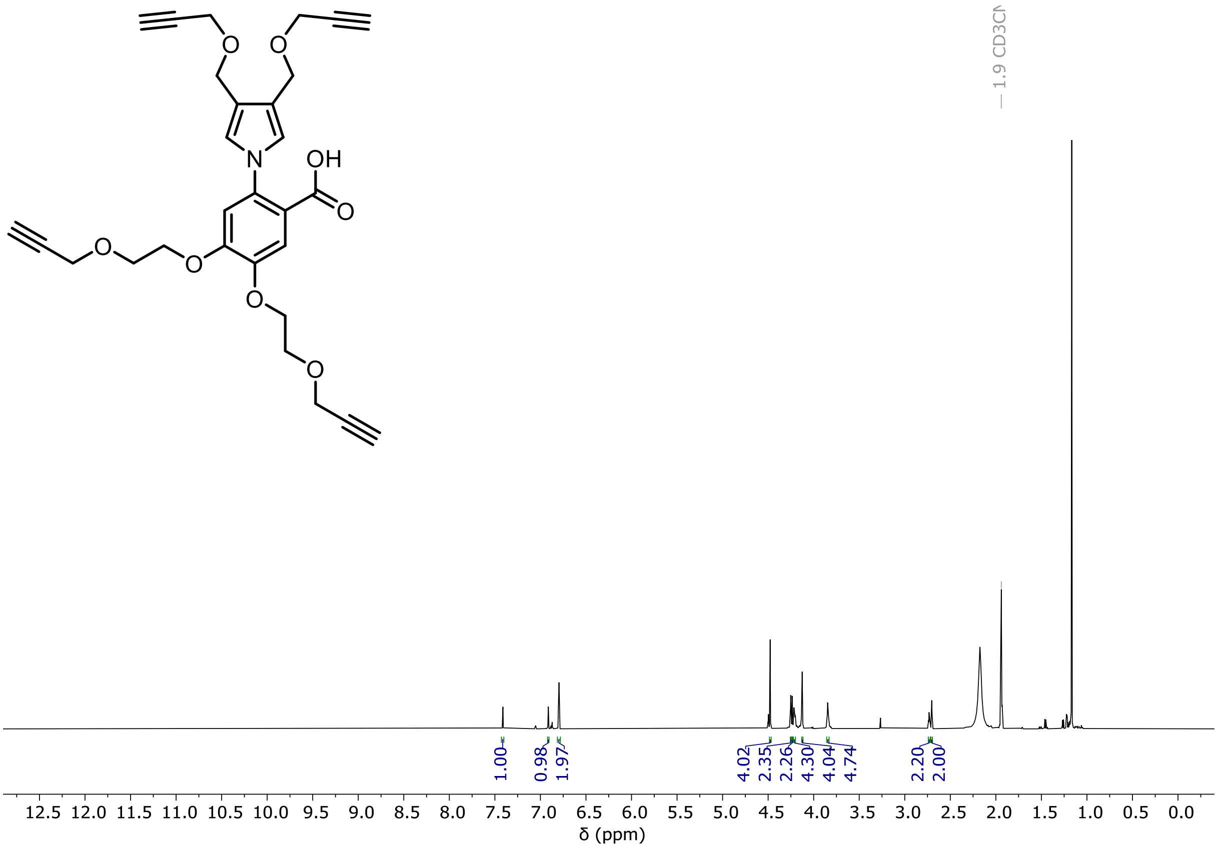

Spectrum S18: ^{1}H NMR spectrum (600 MHz, CD_3CN) of **S14**.

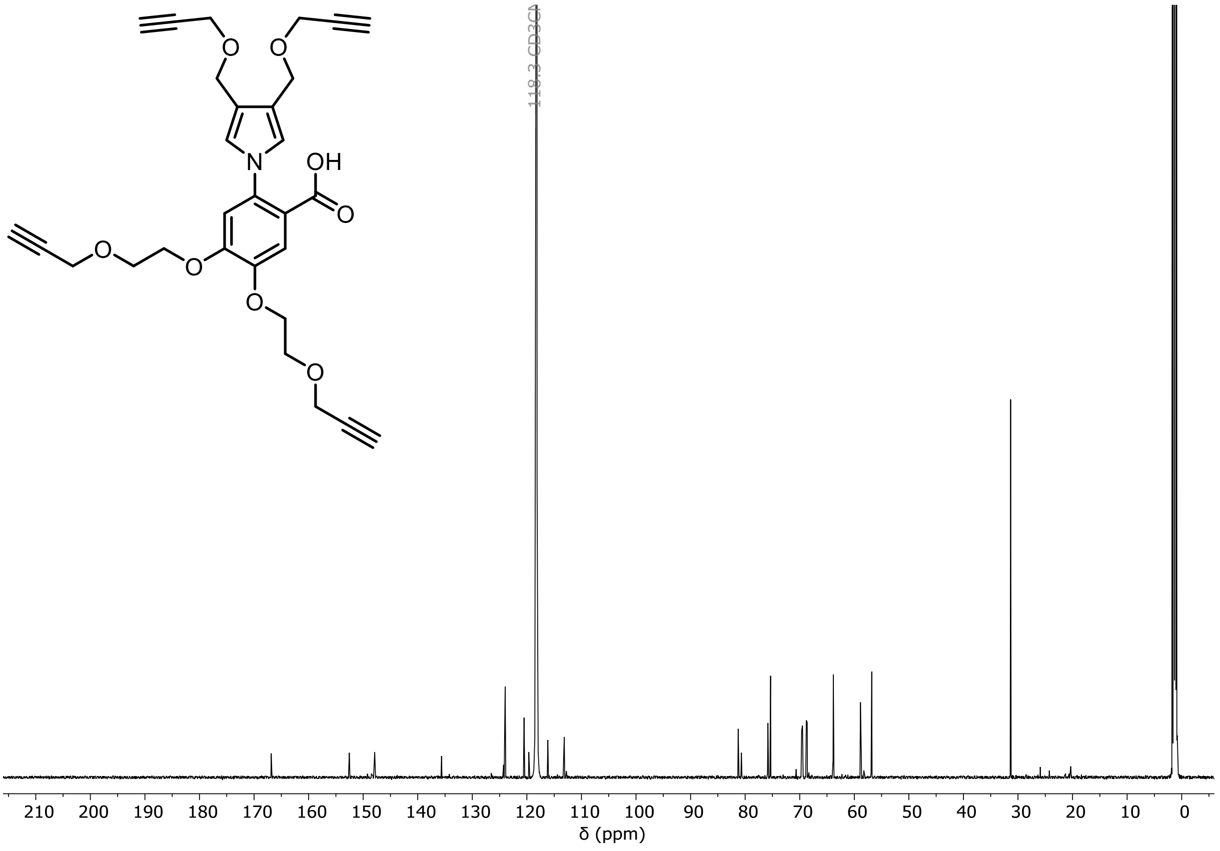

Spectrum S19: ^{13}C NMR spectrum (151 MHz, CD_3CN) of **S14**.

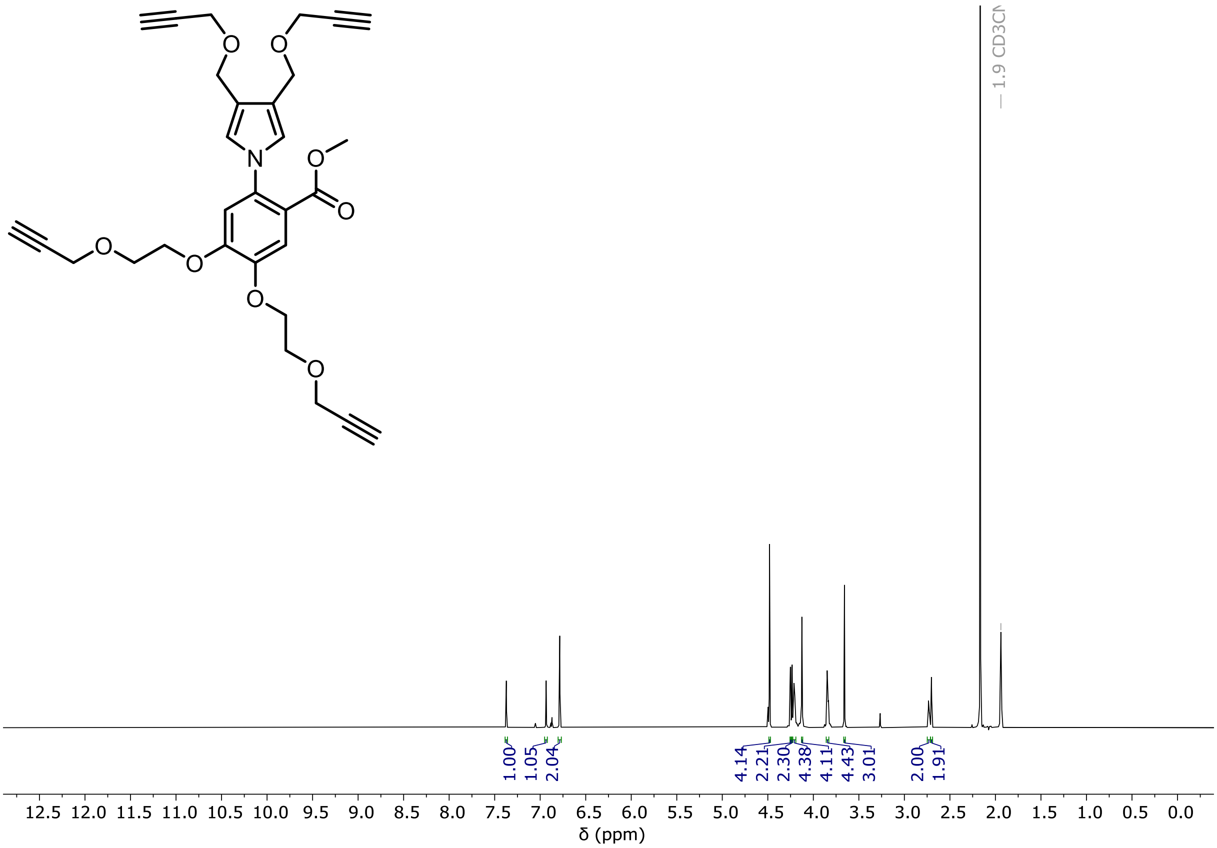

Spectrum S20: ^{1}H NMR spectrum (600 MHz, CD_3CN) of **S15**.

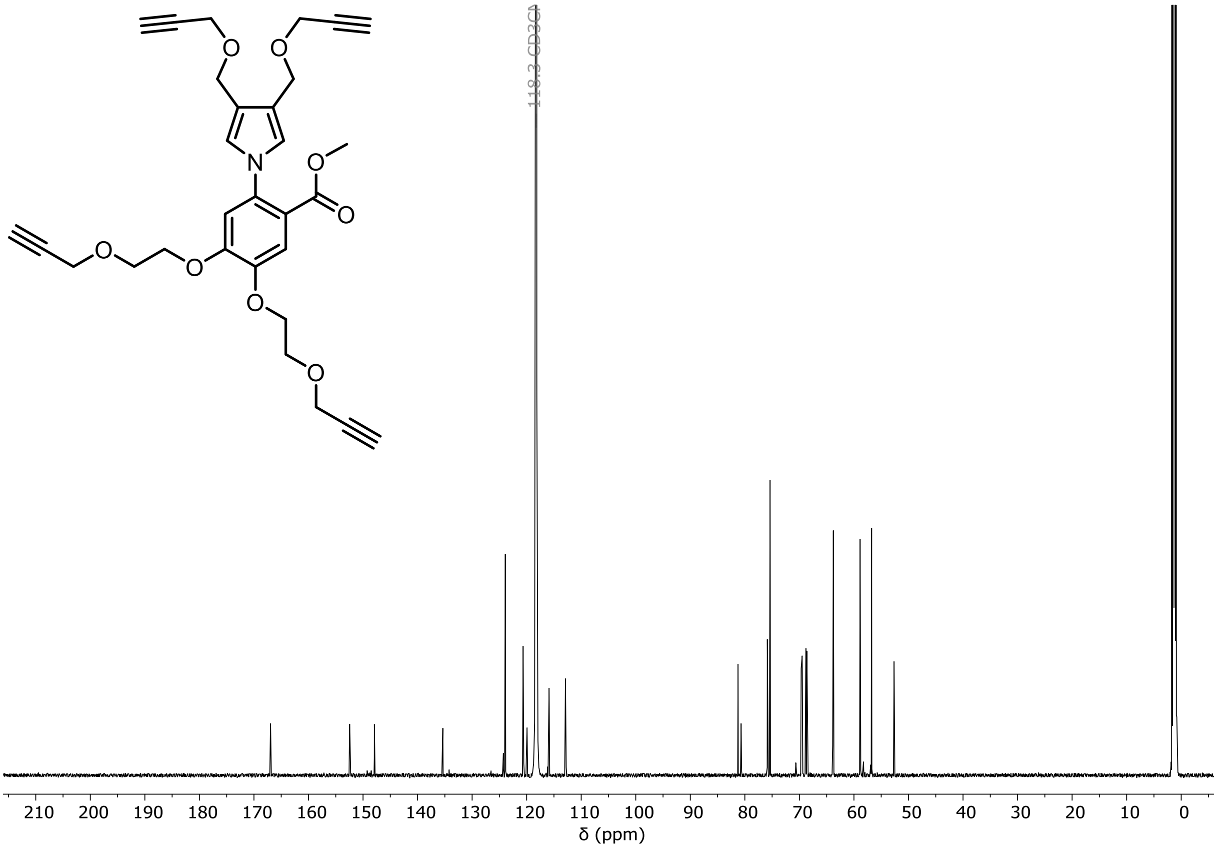

Spectrum S21: ^{13}C NMR spectrum (151 MHz, CD_3CN) of **S15**.

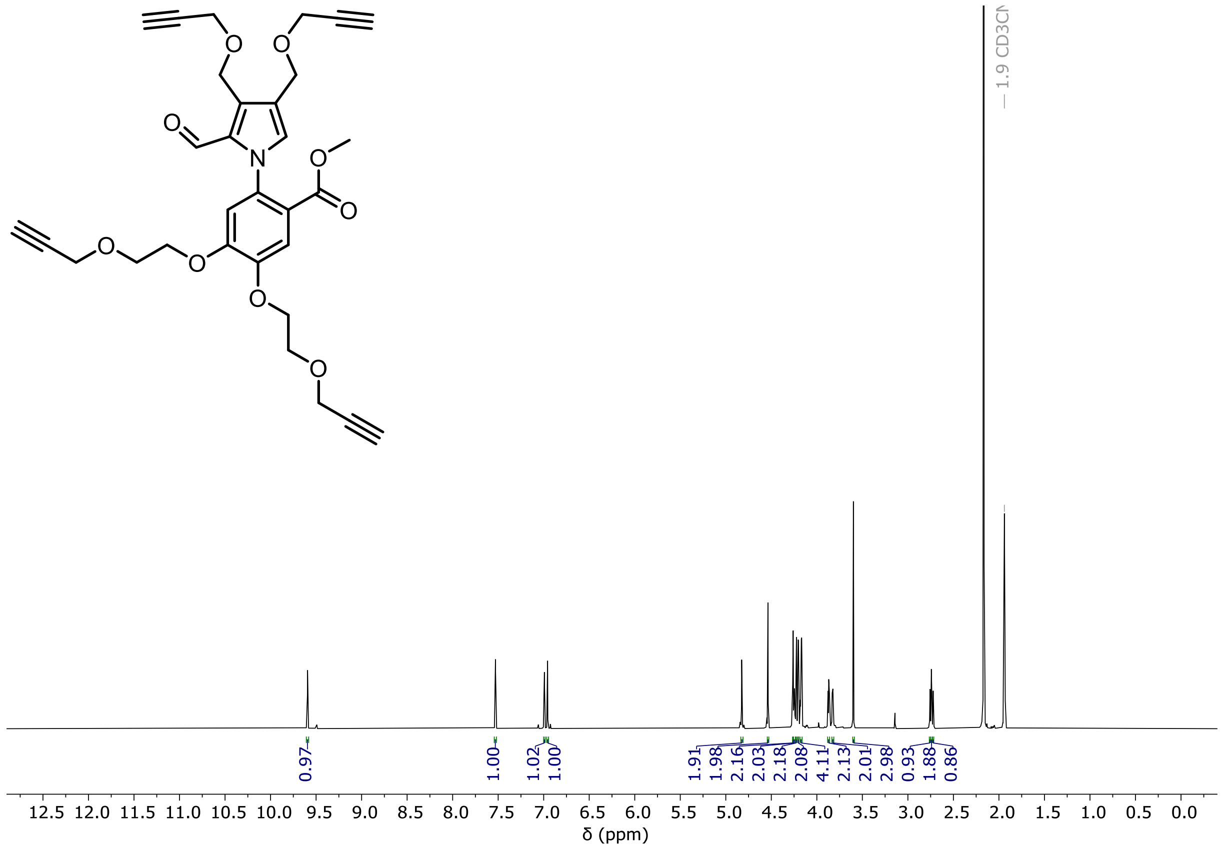

Spectrum S22: ^{1}H NMR spectrum (600 MHz, CD_3CN) of **S16**.

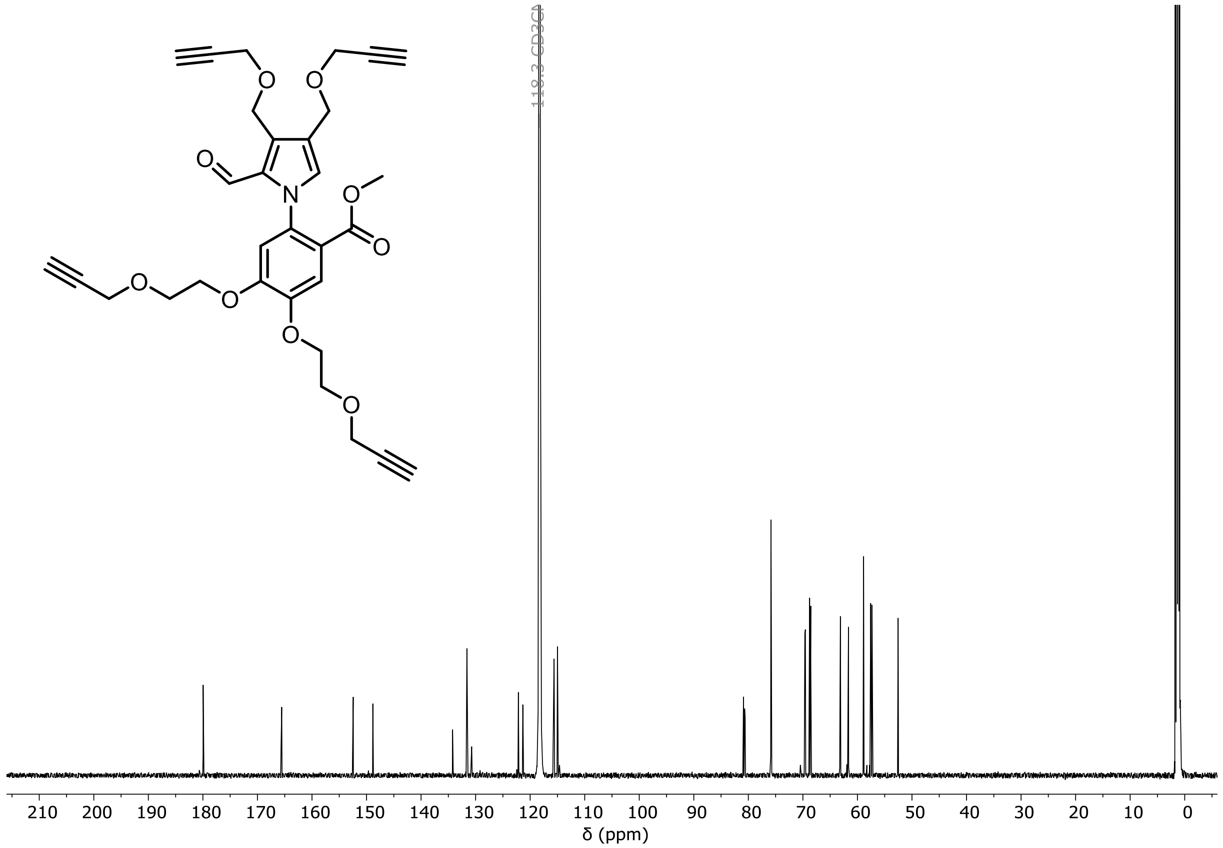

Spectrum S23: ^{13}C NMR spectrum (151 MHz, CD_3CN) of **S16**.

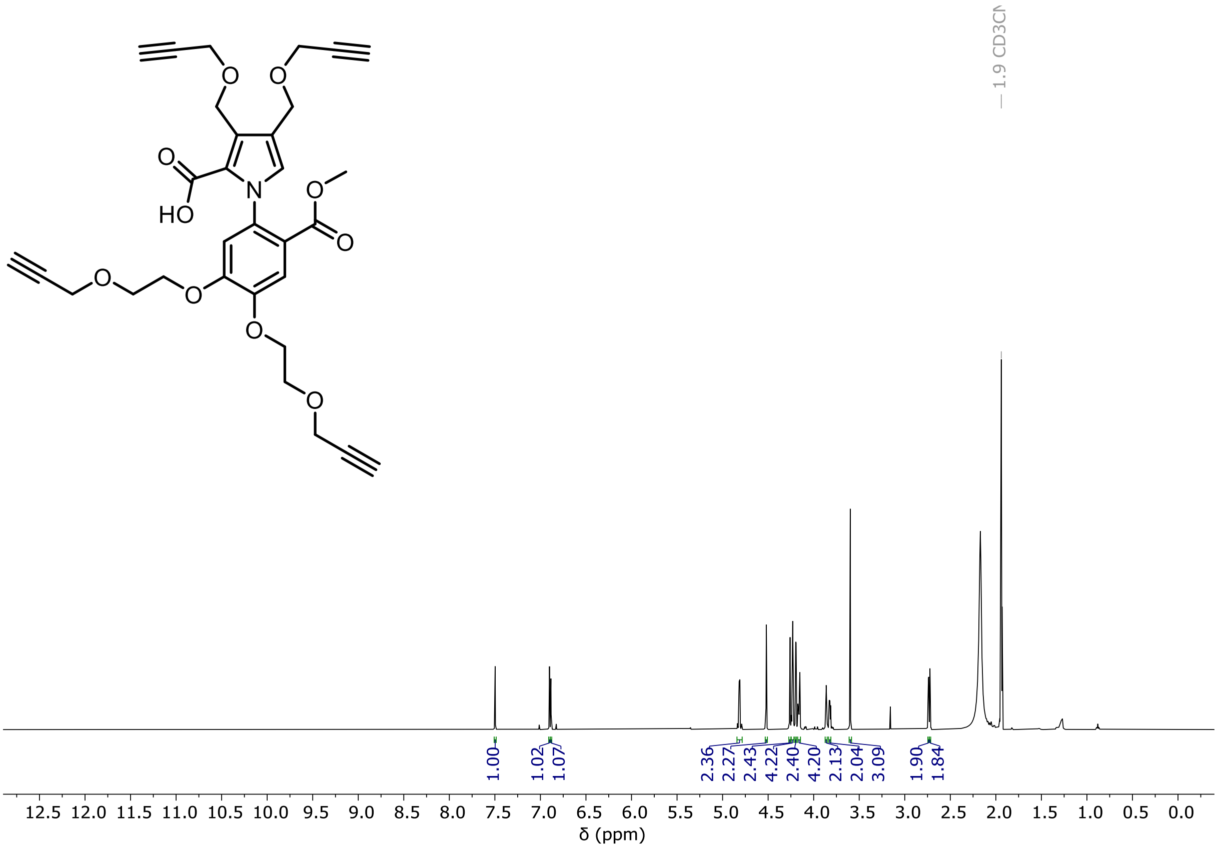

Spectrum S24: ^{1}H NMR spectrum (600 MHz, CD_3CN) of **S17**.

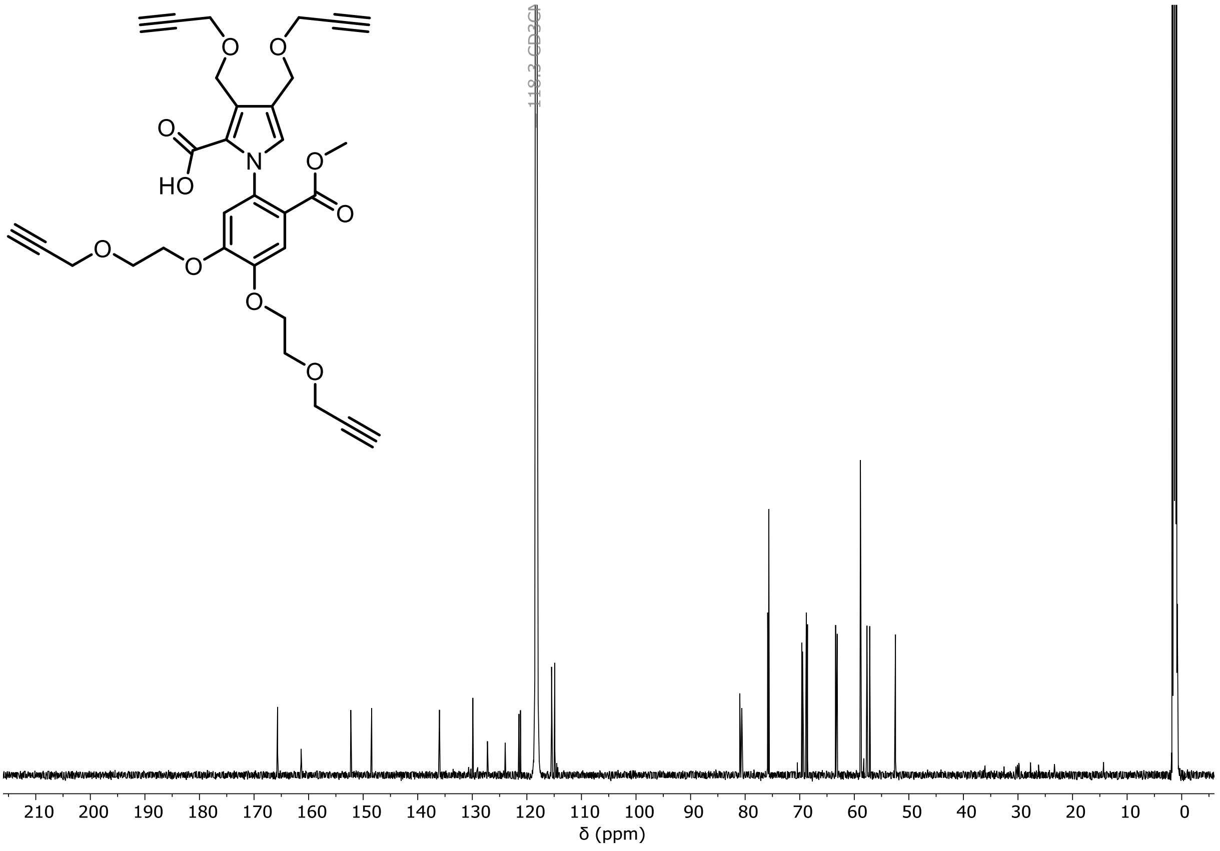

Spectrum S25: ^{13}C NMR spectrum (151 MHz, CD_3CN) of **S17**.

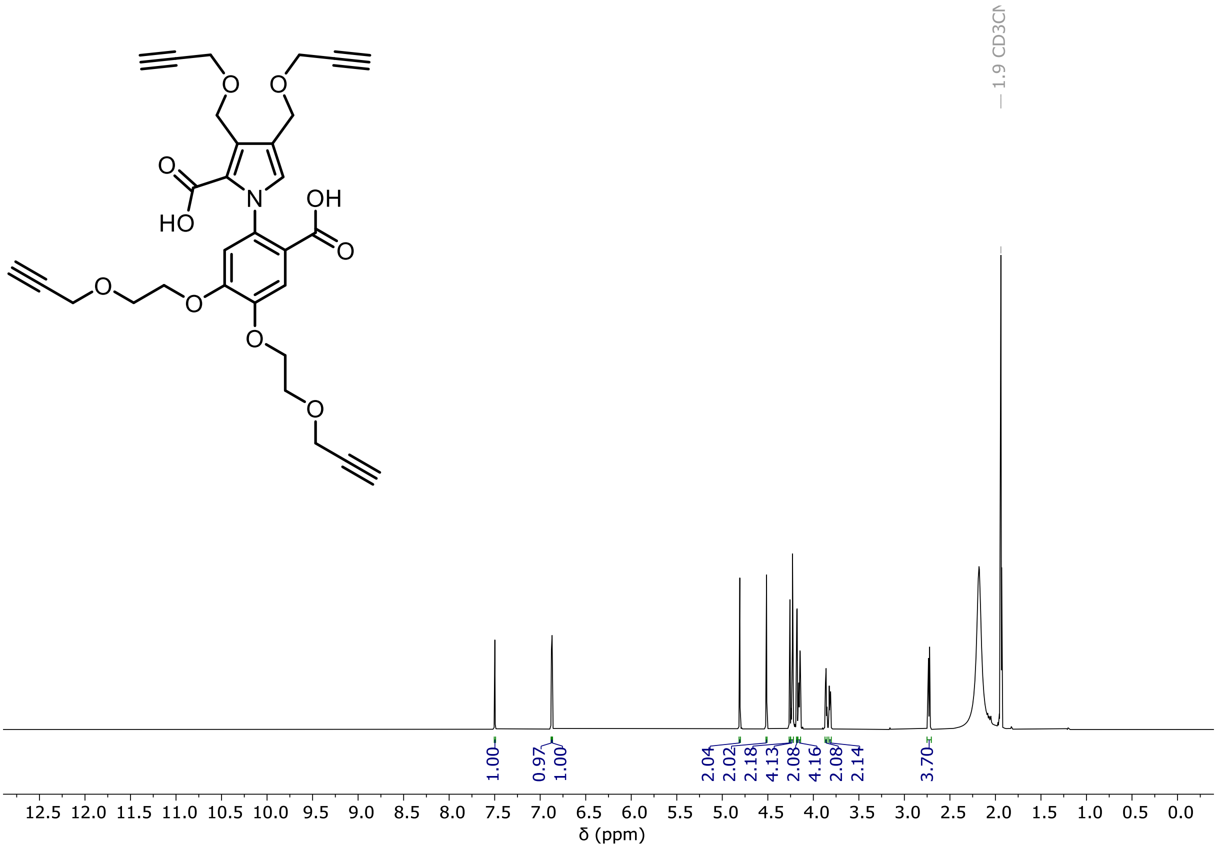

Spectrum S26: ^{1}H NMR spectrum (600 MHz, CD_3CN) of **1**.

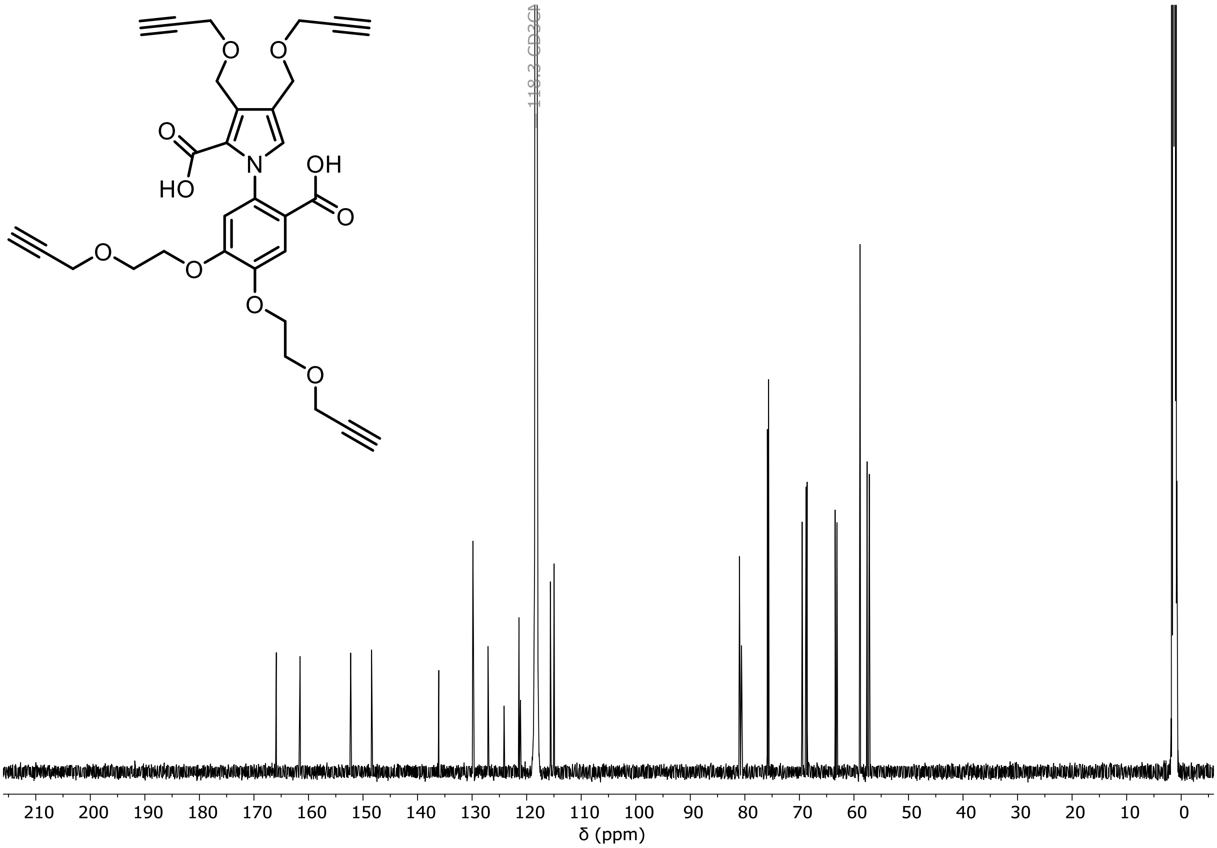

Spectrum S27: ^{13}C NMR spectrum (151 MHz, CD_3CN) of **1**.

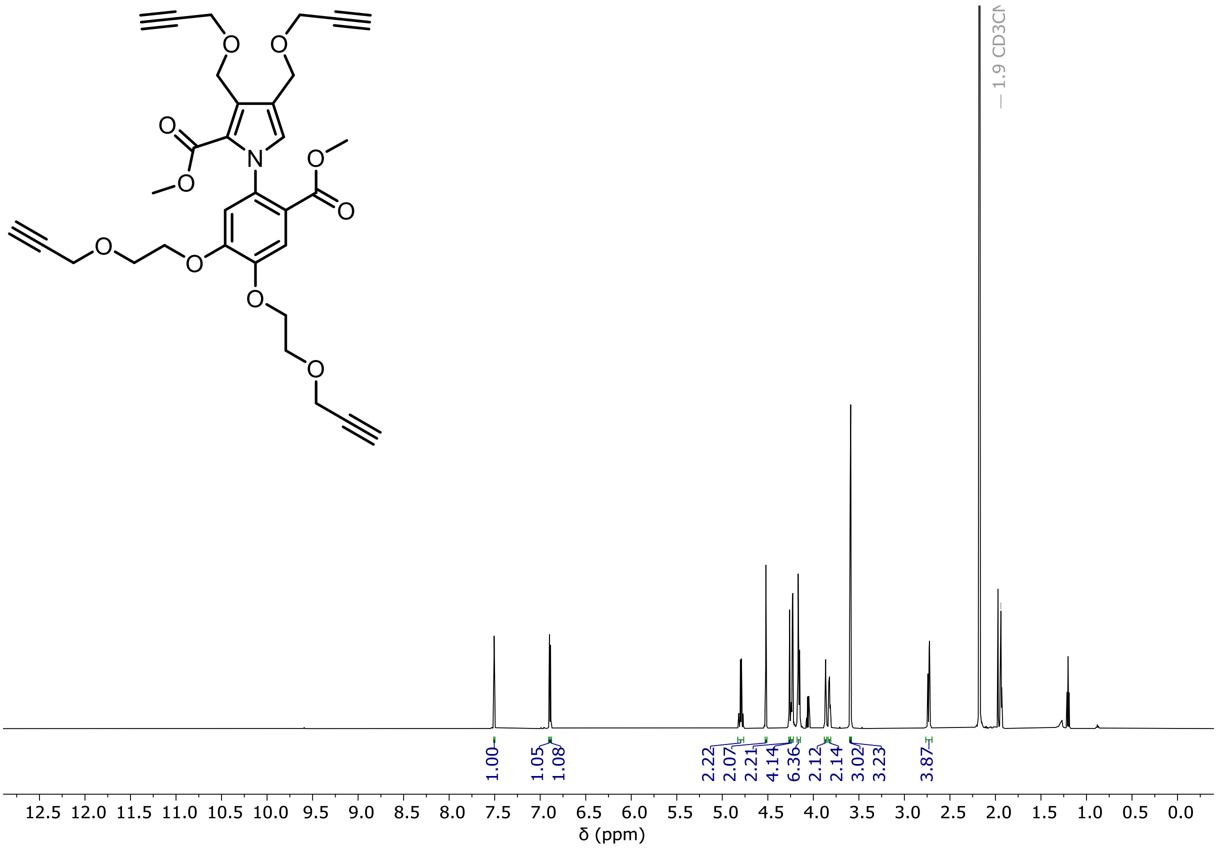

Spectrum S28: ^{1}H NMR spectrum (600 MHz, CD_3CN) of **1**-Me_2.

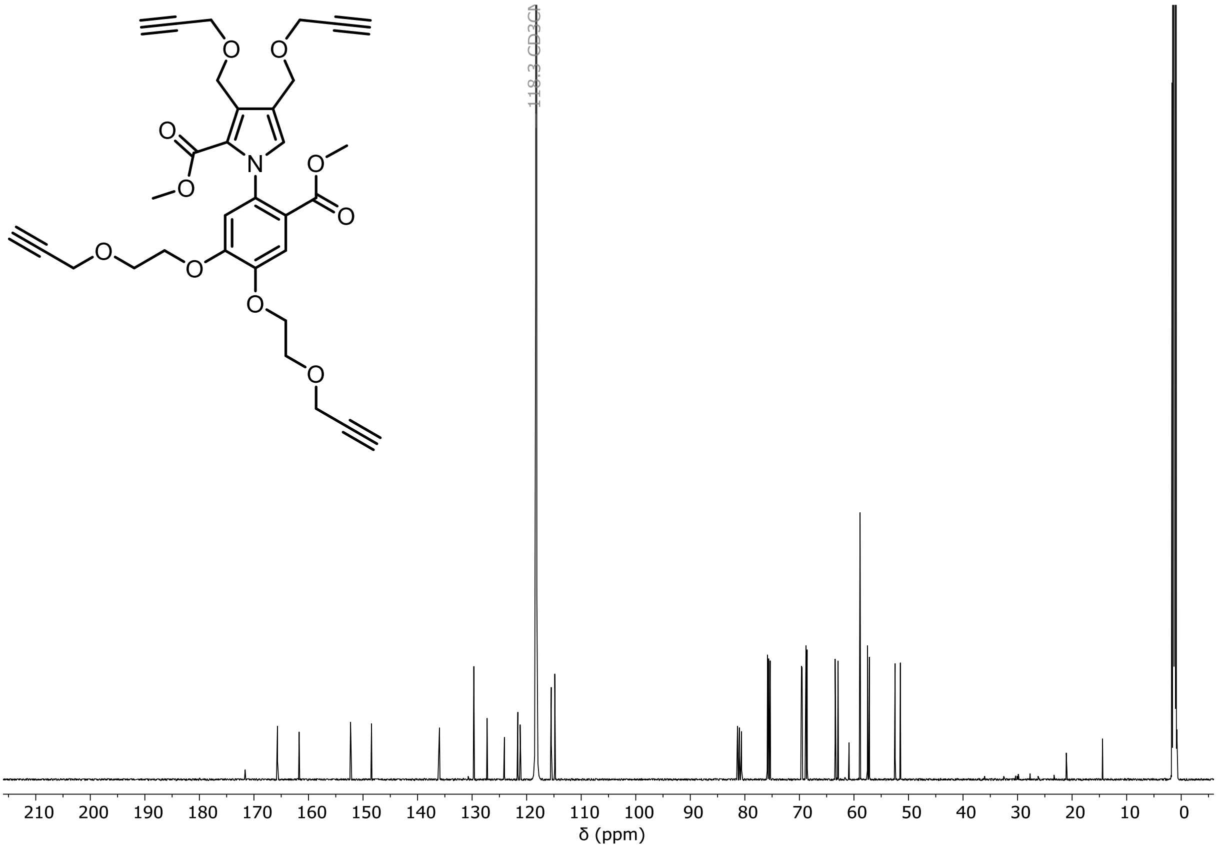

Spectrum S29: ^{13}C NMR spectrum (151 MHz, CD_3CN) of **1**-Me_2.

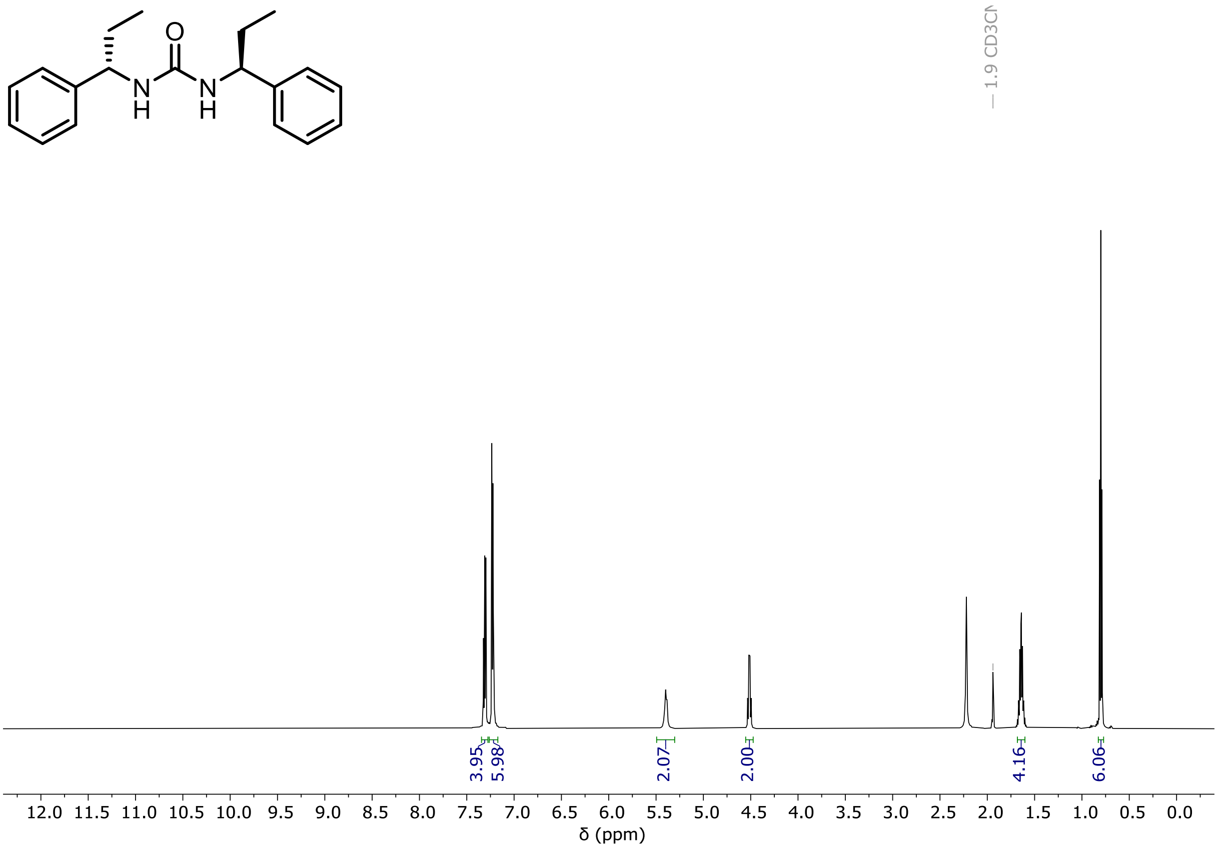

Spectrum S30: ^{1}H NMR spectrum (600 MHz, CD_3CN) of **S18**.

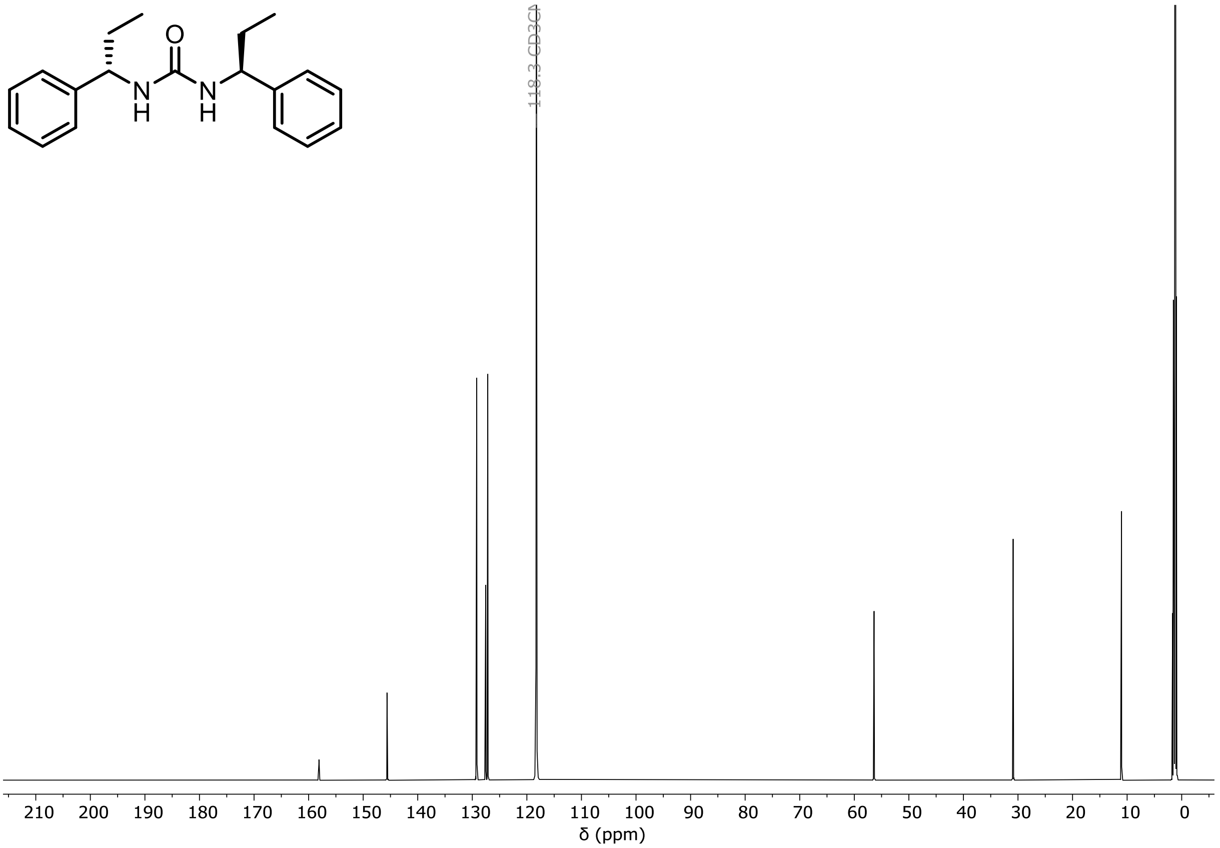

Spectrum S31: ^{13}C NMR spectrum (151 MHz, CD_3CN) of **S18**.

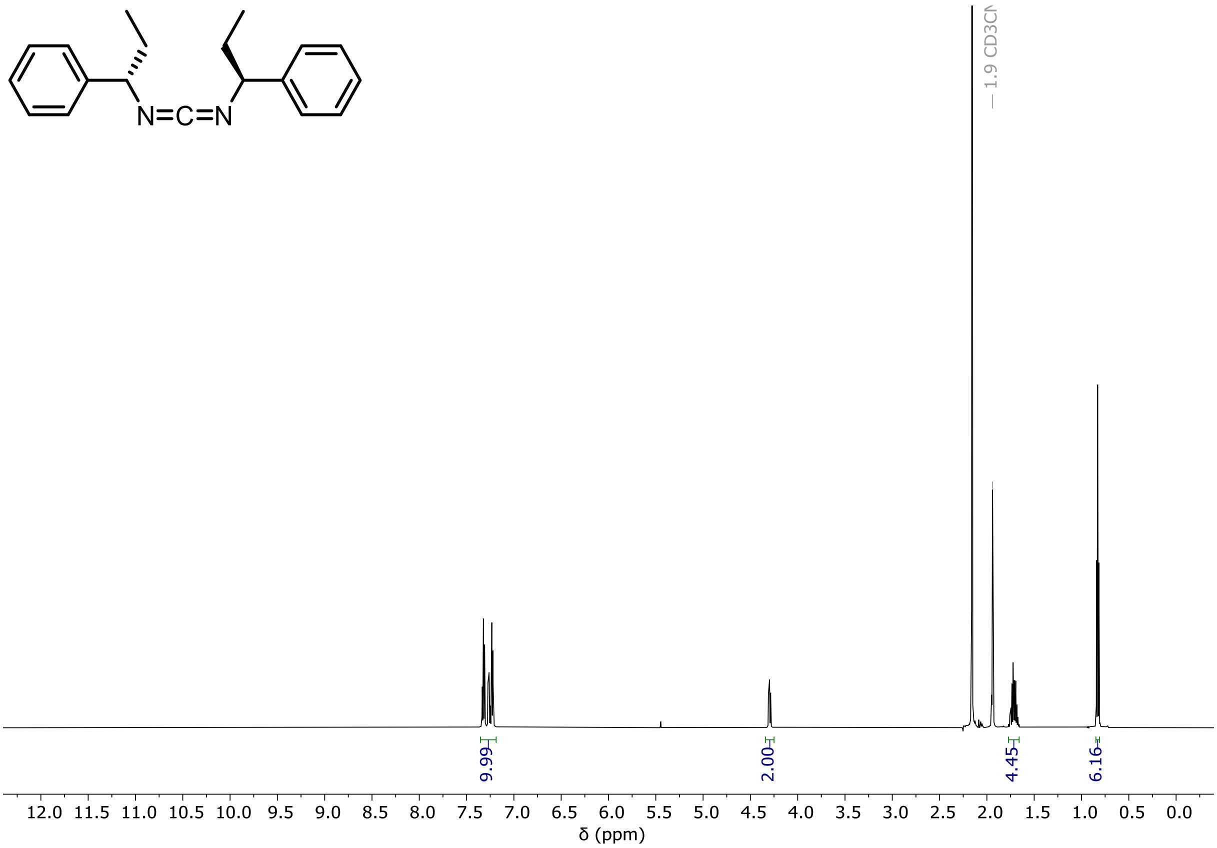

Spectrum S32: ^{1}H NMR spectrum (600 MHz, CD_3CN) of (*S*,*S*)-**2**.

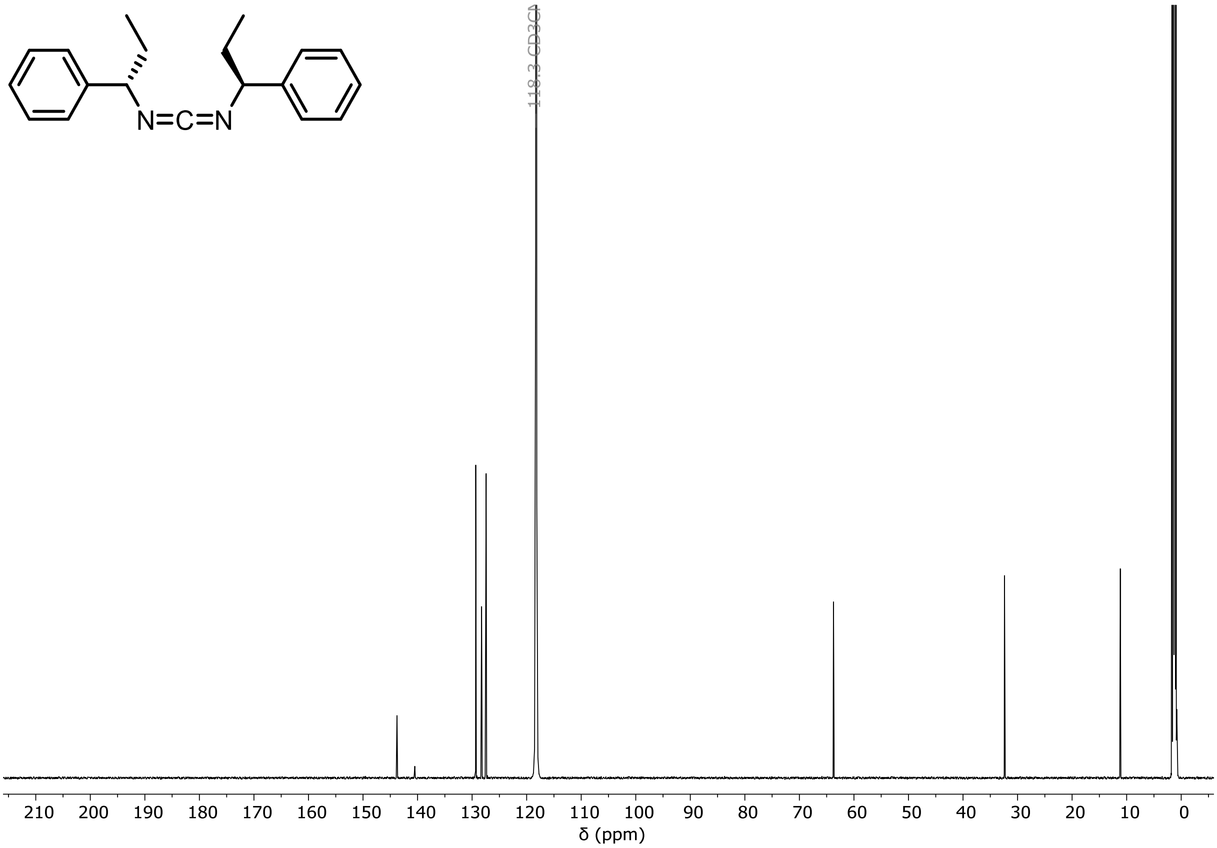

Spectrum S33: ^{13}C NMR spectrum (151 MHz, CD_3CN) of (*S*,*S*)-**2**.

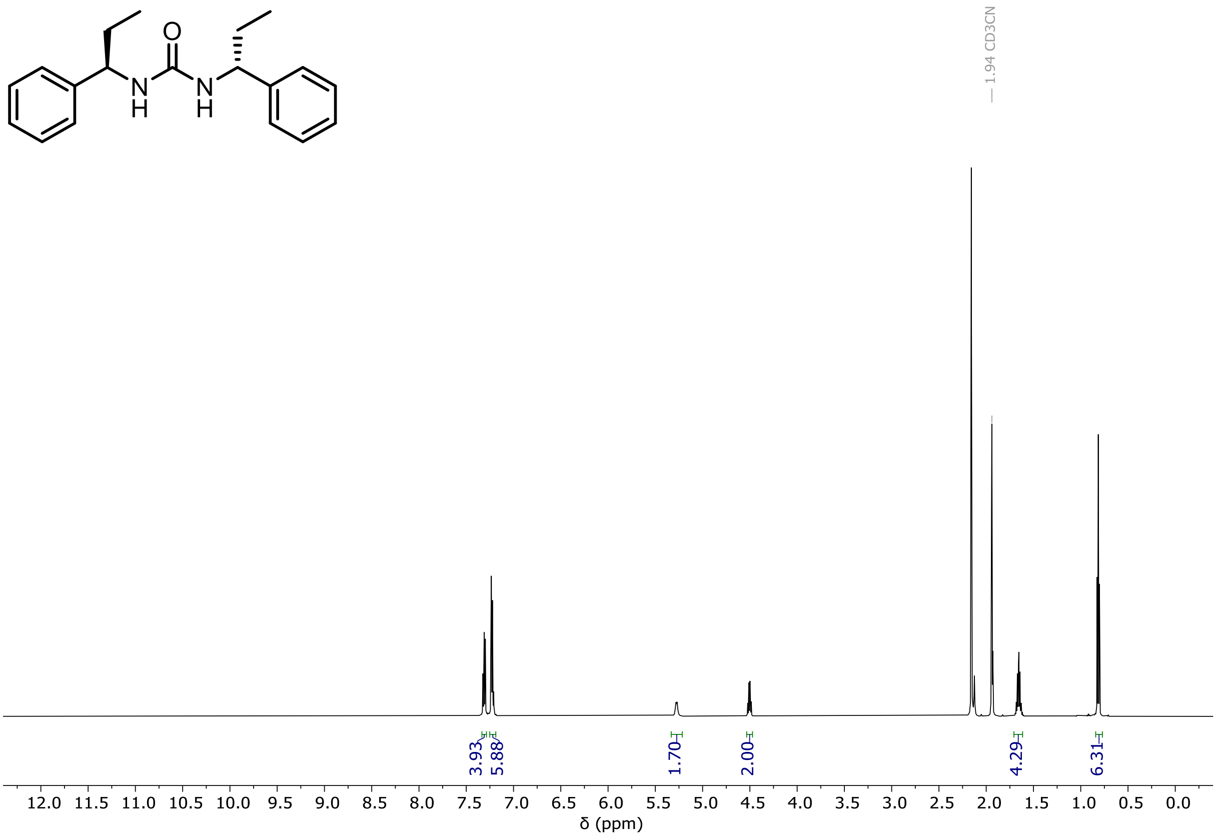

Spectrum S34: ^{1}H NMR spectrum (600 MHz, CD_3CN) of **S19**.

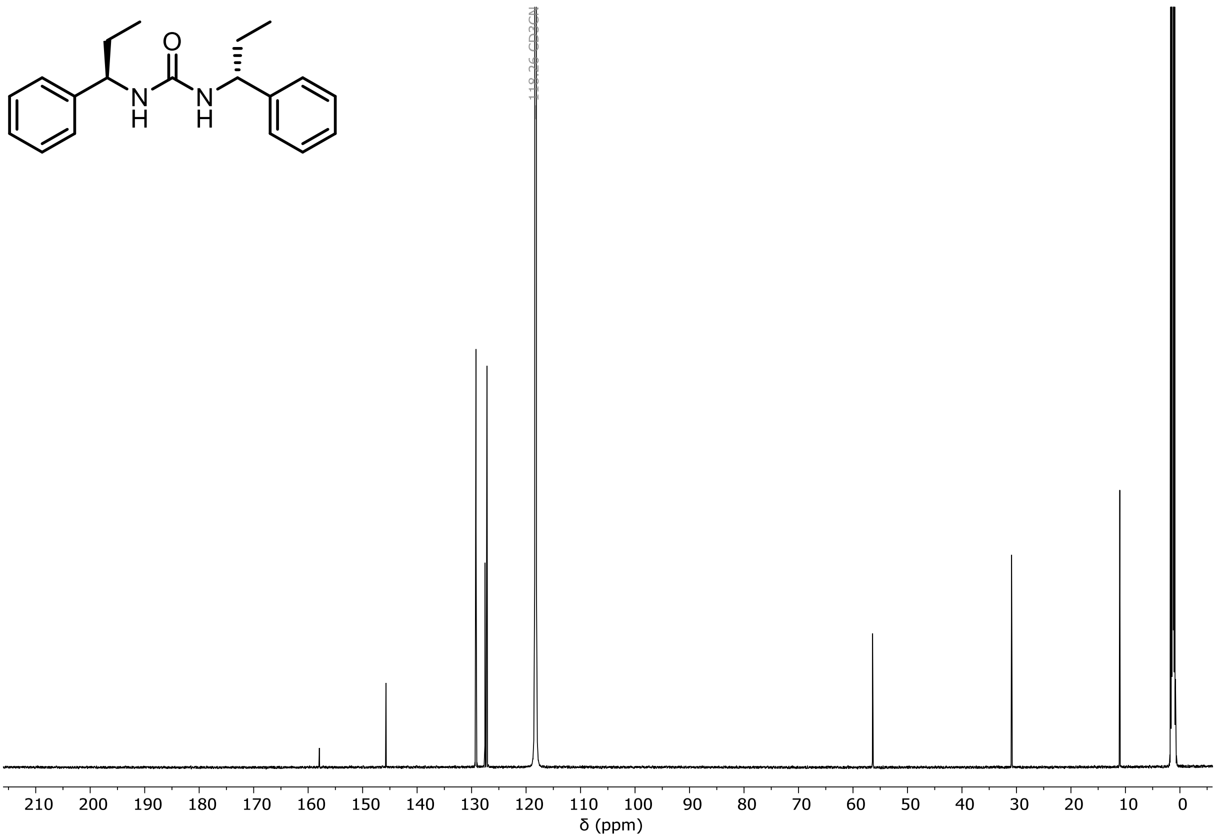

Spectrum S35: ^{13}C NMR spectrum (151 MHz, CD_3CN) of compound **S19**.

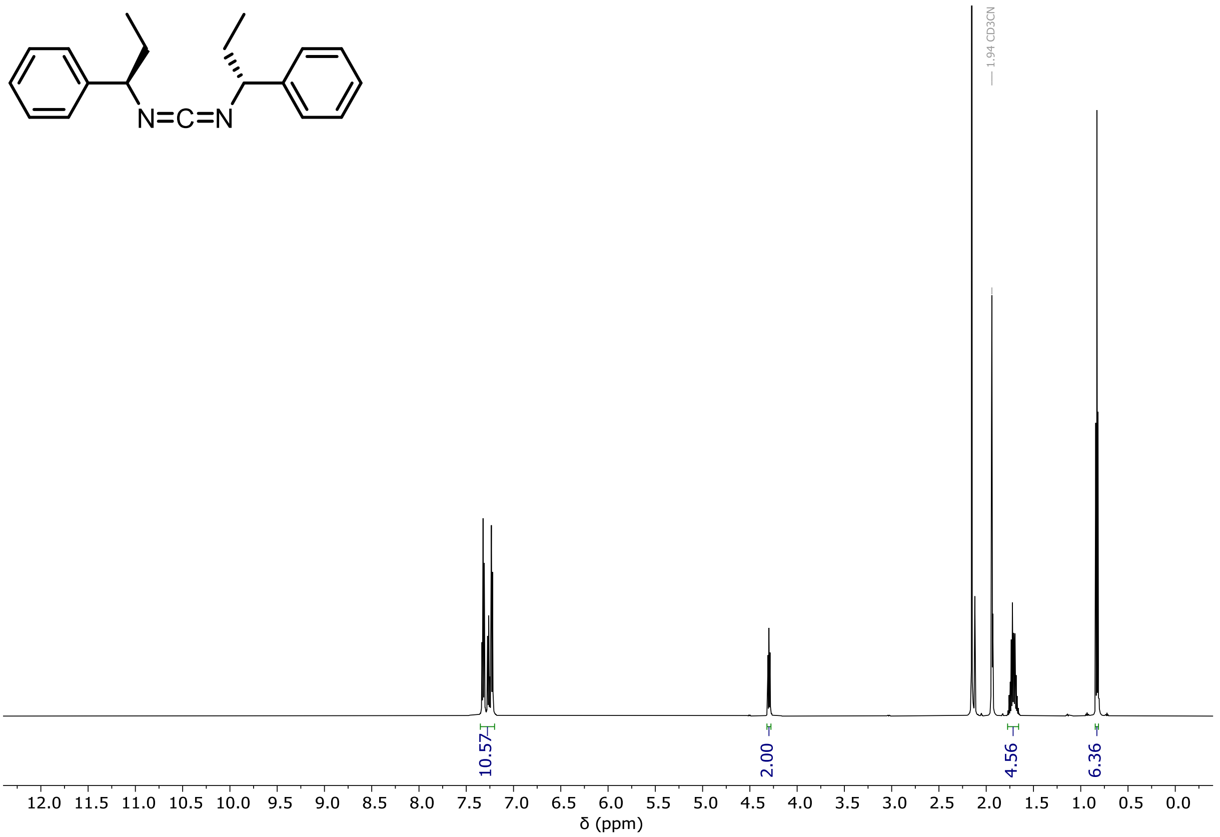

Spectrum S36: ^{1}H NMR spectrum (600 MHz, CD_3CN) of (*R*,*R*)-**2**.

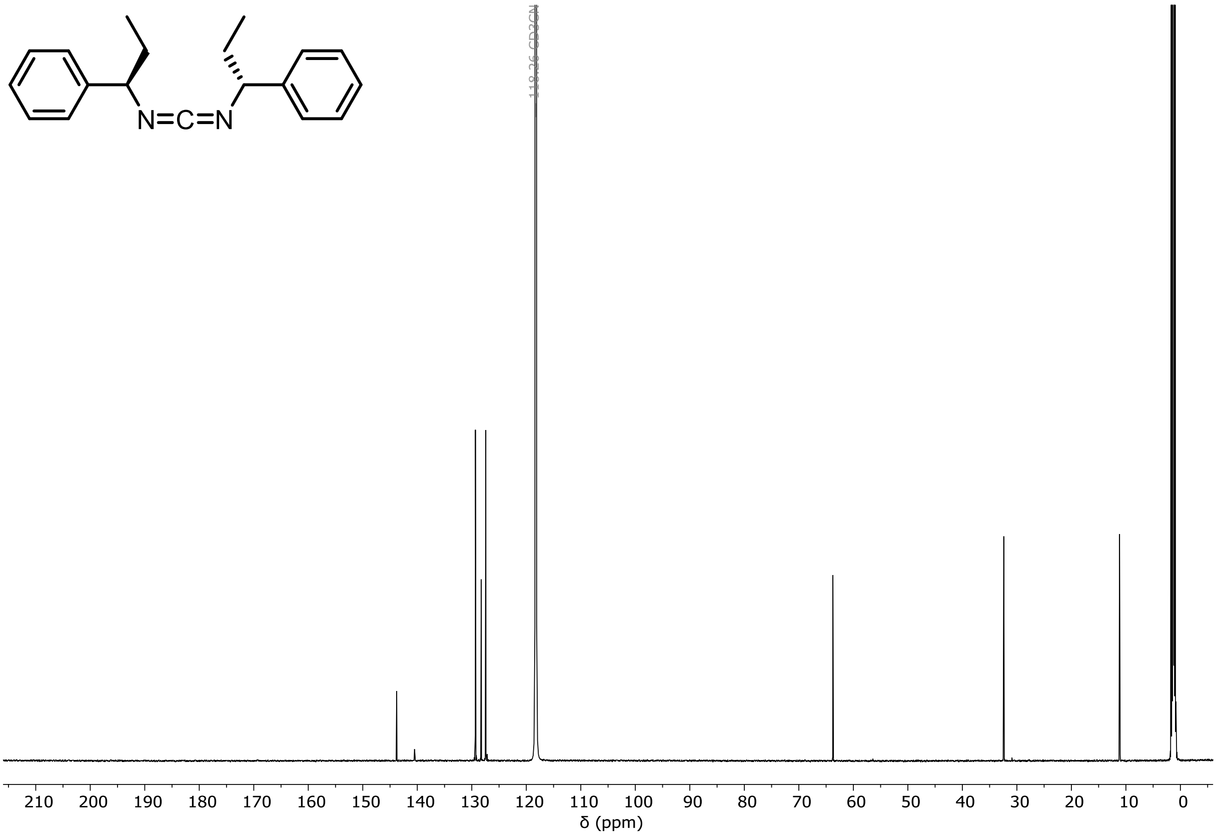

Spectrum S37: ^{13}C NMR spectrum (151 MHz, CD_3CN) of (*R*,*R*)-**2**.

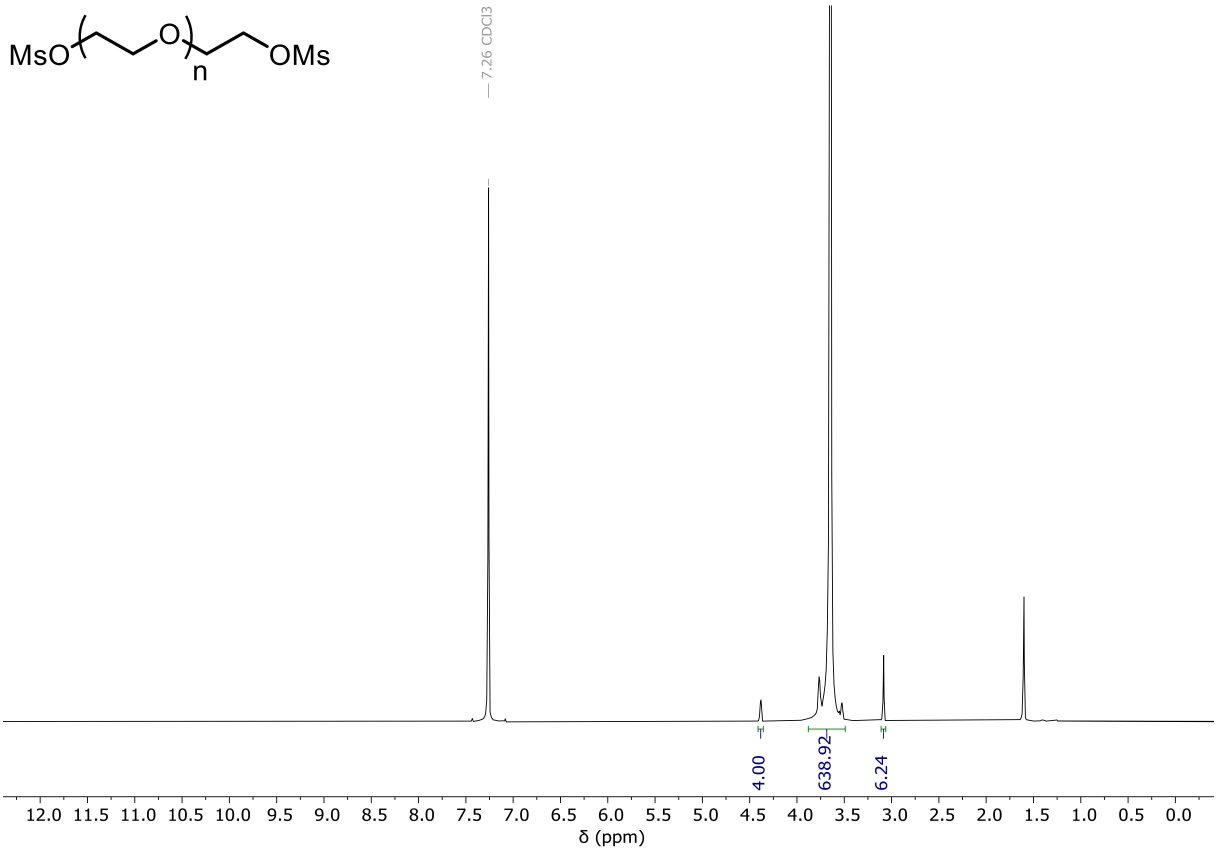

Spectrum S38: ^{1}H NMR spectrum (600 MHz, $CDCl_3$) of α,ω-dimesylate-poly(ethylene glycol) (average M_n 6,000).

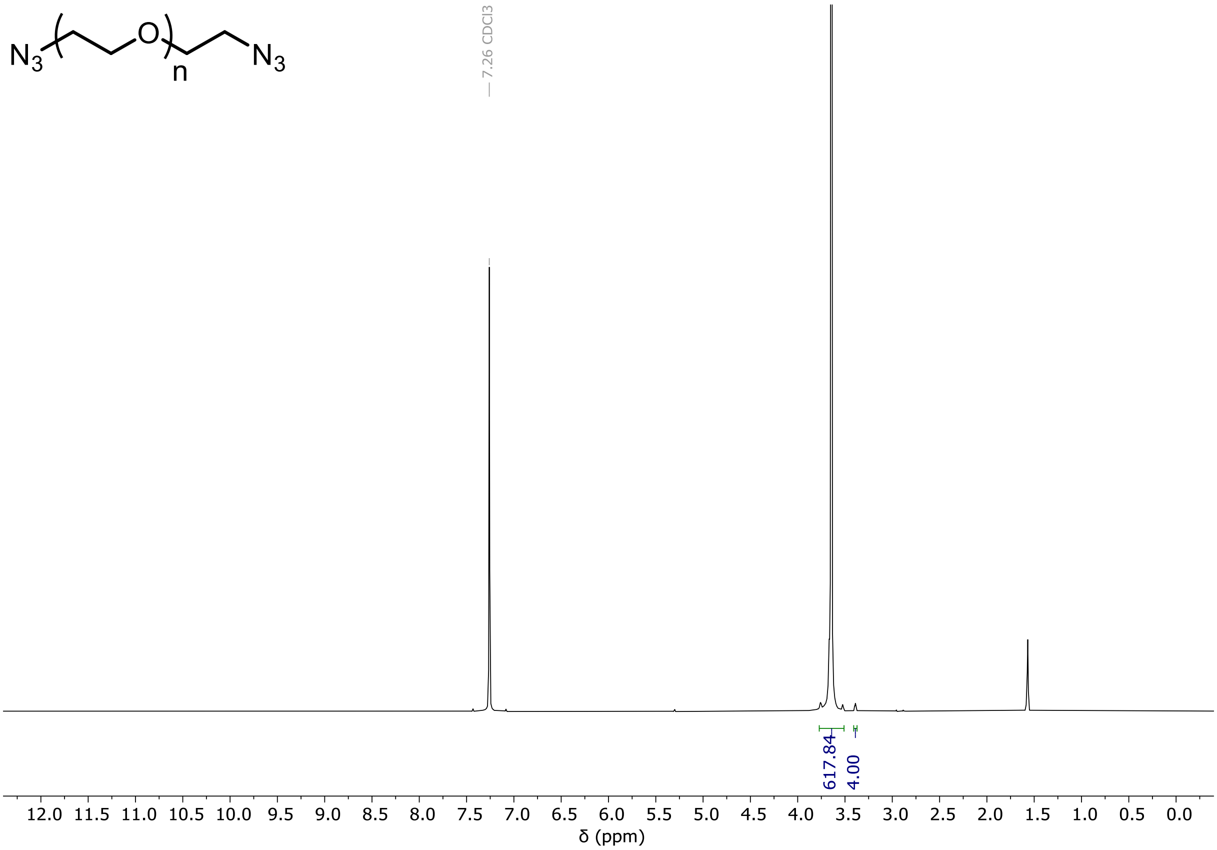

Spectrum S39: ^{1}H NMR spectrum (600 MHz, $CDCl_3$) of α,ω-diazido-poly(ethylene glycol) (average M_n 6,000).

S10. HPLC traces

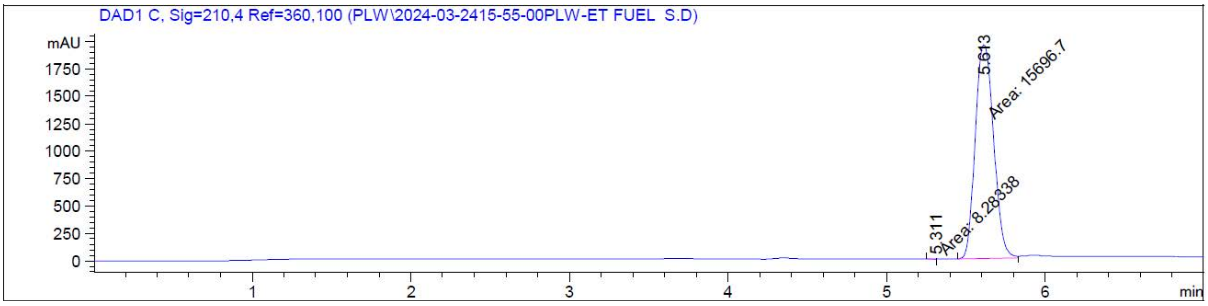

Signal 3: DAD1 C, Sig=210,4 Ref=360,100

Peak #	RetTime [min]	Type	Width [min]	Area [mAU*s]	Height [mAU]	Area %
1	5.311	MM	0.0374	8.28338	3.68702	0.0527
2	5.613	MM	0.1342	1.56967e4	1949.46448	99.9473
Totals :				1.57050e4	1953.15150	

Figure S16. HPLC trace of (*S*,*S*)-**2** (IA column, 25 °C, 1.0 mL/min, *n*-hexane/*i*-propanol = 99/1, *λ* = 210 nm).

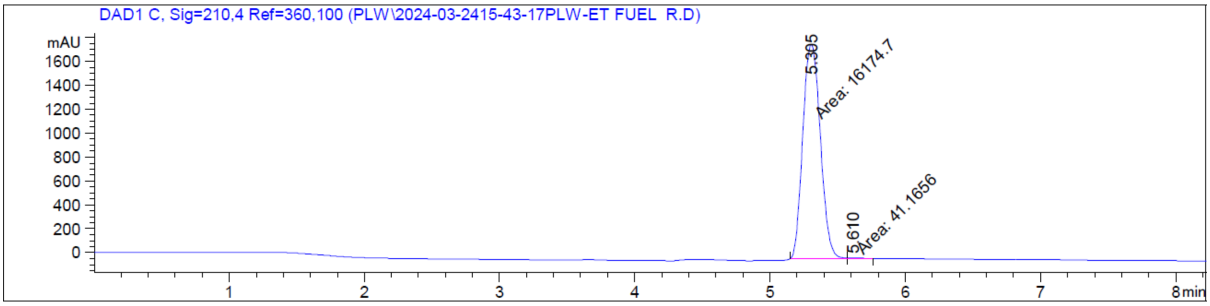

Signal 3: DAD1 C, Sig=210,4 Ref=360,100

Peak #	RetTime [min]	Type	Width [min]	Area [mAU*s]	Height [mAU]	Area %
1	5.305	MF	0.1504	1.61747e4	1792.58313	99.7461
2	5.610	FM	0.1169	41.16557	5.87098	0.2539
Totals :				1.62159e4	1798.45411	

Figure S17. HPLC trace of (*R*,*R*)-**2** (IA column, 25 °C, 1.0 mL/min, *n*-hexane/*i*-propanol = 99/1, *λ* = 210 nm).

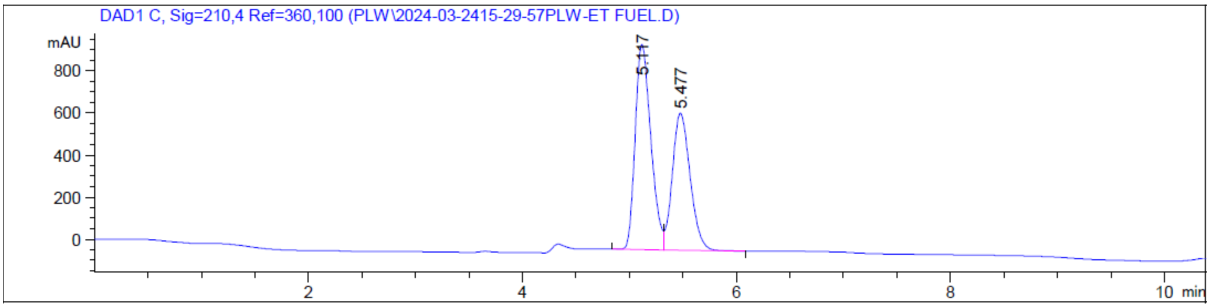

Signal 3: DAD1 C, Sig=210,4 Ref=360,100

Peak #	RetTime [min]	Type	Width [min]	Area [mAU*s]	Height [mAU]	Area %
1	5.117	BV	0.1567	9912.45313	974.40405	56.7545
2	5.477	VB	0.1794	7553.04346	650.76587	43.2455
Totals :				1.74655e4	1625.16992	

Figure S18. HPLC trace of (*R*,*R*)-**2** and (*S*,*S*)-**2** (IA column, 25 °C, 1.0 mL/min, *n*-hexane/*i*-propanol = 99/1, λ = 210 nm).

S11. Mass spectra

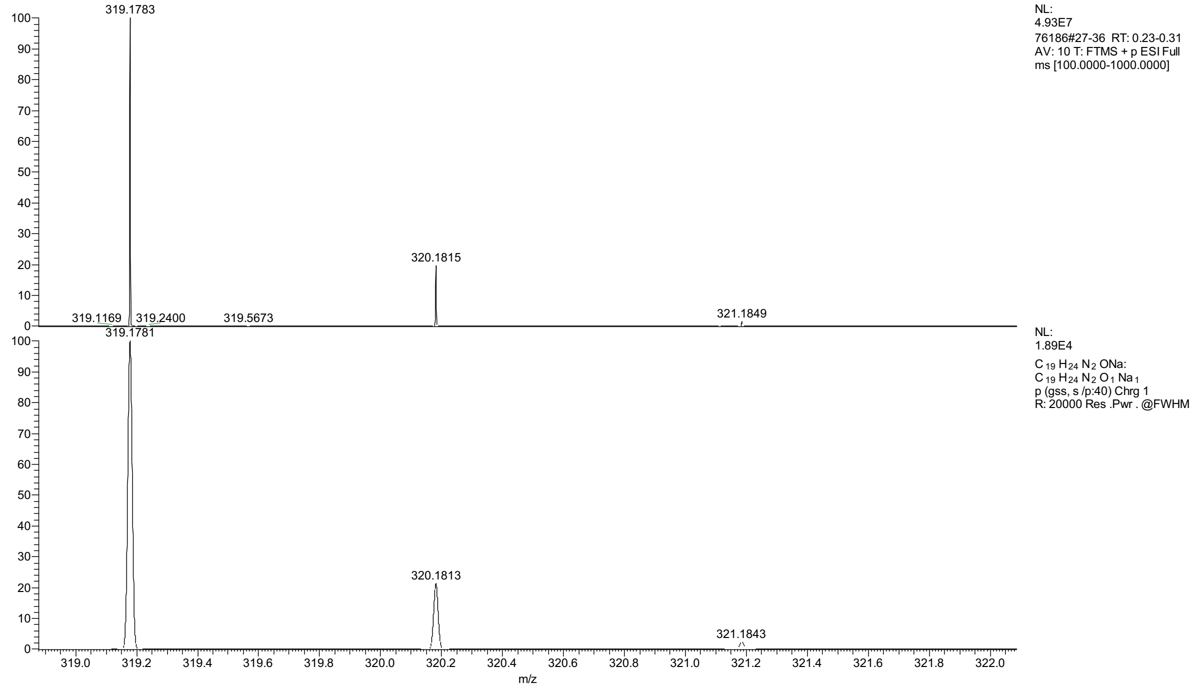

Figure S19. High-resolution ESI(+)-MS of compound **1**.

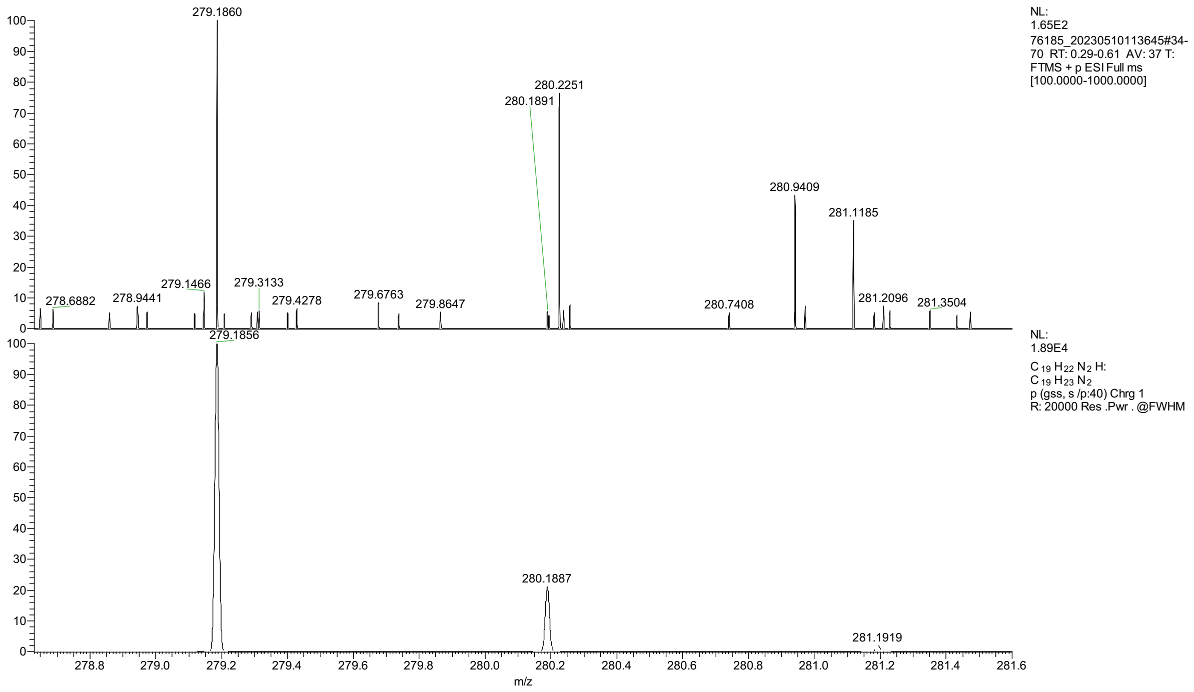

Figure S20. High-resolution ESI(+)-MS of compound (*S*,*S*)-**2.**

S12. References

S1. Amanullah, S. & Dey, A. The role of porphyrin peripheral substituents in determining the reactivities of ferrous nitrosyl species. *Chem. Sci.* **11**, 5909–5921 (2020).

S2. Reitz, A., Avery, M. A., Verlander, M. S. & Goodman, M. Synthesis of ring-alkylated isoproterenol derivatives. *J. Org. Chem.* **46**, 4859–4863 (1981).

S3. Cudaj, J. & Podlech, J. Total synthesis of altenusin and alterlactone. *Synlett* **3**, 371–374 (2012).

S4. Ó Dálaigh, C. & Connon, S. J. Nonenzymatic acylative kinetic resolution of Baylis−Hillman adducts. *J. Org. Chem.* **72**, 7066–7069 (2007).

S5. Borsley, S., Kreidt, E., Leigh, D. A. & Roberts, B. M. W. Autonomous fuelled directional rotation about a covalent single bond. *Nature* **604**, 80–85 (2022).

S6. Perrot, A., Wang, W.-Z., Buhler, E., Moulin, E. & Giuseppone, N. Bending actuation of hydrogels through rotation of light-driven molecular motors. *Angew. Chem. Int. Ed.* **62**, e20230026 (2023).

S7. Yang, X., Dargaville, B. L. & Hutmacher, D. W. Elucidating the molecular mechanisms for the interaction of water with polyethylene glycol-based hydrogels: influence of ionic strength and gel network structure. *Polymers* **13**, 845 (2021).

S8. Yavari, N. & Azizian, S. Mixed diffusion and relaxation kinetics model for hydrogels swelling. *J. Mol. Liq.* **363**, 119861 (2022).

S9. Sakai, T. et al. Design and fabrication of a high-strength hydrogel with ideally homogeneous network structure from tetrahedron-like macromonomers. *Macromolecules* **41**, 5379–5384 (2008).

S10. Sukumaran, S. K. & Beaucage, G. A structural model for equilibrium swollen networks. *Europhys. Lett.* **59**, 714–720 (2002).

S11. Obukhov, S. P., Rubinstein, M. & Colby, R. H. Network modulus and superelasticity. *Macromolecules* **27**, 3191–3198 (1994).

S12. Binks, L. et al. The role of kinetic asymmetry and power strokes in an information ratchet. *Chem* **9**, 2902–2917 (2023).
